# The causal role of circulating inflammatory markers in osteoporosis: a bidirectional Mendelian randomized study

**DOI:** 10.3389/fimmu.2024.1412298

**Published:** 2024-07-18

**Authors:** Qiu Dong, Jiayang Wu, Huaguo Zhang, Liangping Luo, Wenrui Wu

**Affiliations:** ^1^ Department of Bone and Joint Surgery, First Affiliated Hospital of Jinan University, Guangzhou, Guangdong, China; ^2^ Medical Imaging Centre, First Affiliated Hospital of Jinan University, Guangzhou, Guangdong, China; ^3^ Department of Ultrasonography, First Affiliated Hospital of Jinan University, Guangzhou, Guangdong, China; ^4^ Medical Imaging Center, The Fifth Affiliated Hospital of Jinan University, Heyuan, Guangdong, China; ^5^ Department of Orthopedics, Chaoshan Hospital, The First Affiliated Hospital of Jinan University, Chaozhou, Guangdong, China

**Keywords:** Mendelian randomization, circulating inflammatory protein, osteoporosis, Bayesian colocalization analysis, artemin (ARTN)

## Abstract

**Background:**

Osteoporosis (OP) associated with aging exerts substantial clinical and fiscal strains on societal structures. An increasing number of research studies have suggested a bidirectional relationship between circulating inflammatory markers (CIMs) and OP. However, observational studies are susceptible to perturbations in confounding variables. In contrast, Mendelian randomization (MR) offers a robust methodological framework to circumvent such confounders, facilitating a more accurate assessment of causality. Our study aimed to evaluate the causal relationships between CIMs and OP, identifying new approaches and strategies for the prevention, diagnosis and treatment of OP.

**Methods:**

We analyzed publicly available GWAS summary statistics to investigate the causal relationships between CIMs and OP. Causal estimates were calculated via a systematic analytical framework, including bidirectional MR analysis and Bayesian colocalization analysis.

**Results:**

Genetically determined levels of CXCL11 (OR = 0.91, 95% CI = 0.85–0.98, P = 0.008, P_FDR_ = 0.119), IL-18 (OR = 0.88, 95% CI = 0.83–0.94, P = 8.66×10–5, P_FDR_ = 0.008), and LIF (OR = 0.86, 95% CI = 0.76–0.96, P = 0.008, P_FDR_ = 0.119) were linked to a reduced risk of OP. Conversely, higher levels of ARTN (OR = 1.11, 95% CI = 1.02–1.20, P = 0.012, P_FDR_ = 0.119) and IFNG (OR = 1.16, 95% CI = 1.03–1.30, P = 0.013, P_FDR_ = 0.119) were associated with an increased risk of OP. Bayesian colocalization analysis revealed no evidence of shared causal variants.

**Conclusion:**

Despite finding no overall association between CIMs and OP, five CIMs demonstrated a potentially significant association with OP. These findings could pave the way for future mechanistic studies aimed at discovering new treatments for this disease. Additionally, we are the first to suggest a unidirectional causal relationship between ARTN and OP. This novel insight introduces new avenues for research into diagnostic and therapeutic strategies for OP.

## 1 Introduction

Osteoporosis (OP) is a severe skeletal disorder characterized by a high incidence and mortality rate. It is characterized by decreased bone strength, increased fragility, and a propensity for fractures, which can lead to cardiovascular diseases and even premature mortality (1–7). Currently, OP affects approximately 18.3% of the global population, and its incidence is increasing due to environmental pollution and an aging population, highlighting the need for vigilance (8). However, regrettably, the current approach to OP treatment primarily focuses on prevention, utilizing pharmacological interventions to slow bone loss while ensuring adequate nutrition. Nevertheless, the clinical diagnosis of OP relies heavily on dual-energy X-ray absorptiometry (DEXA), and a convenient method for widespread use is not available. This may explain the higher prevalence of OP in underdeveloped regions. Therefore, in-depth research on the pathogenesis of OP is urgently needed for the early prevention and accurate diagnosis of OP and to develop effective treatment strategies.

Circulating inflammatory markers (CIMs) are garnering increasing interest in medical research because of their potential to offer critical insights into early disease diagnosis, prognosis evaluation, and therapeutic strategies. These small molecule proteins, which are produced by immune cells, play pivotal roles in regulating and controlling immune and inflammatory responses. Through intercellular signal transduction, CIMs regulate and coordinate the body’s responses to infections, injuries, or other stimuli. These CIMs are widely present within the body and can interact with various immune cells, including macrophages, lymphocytes, and granulocytes. As crucial modulators of the inflammatory process, they participate in key biological processes, including cell proliferation, differentiation, migration, and apoptosis. Common CIMs include tumor necrosis factor (TNF), interleukin (IL) family members, interferon (IFN) family members, and chemokines. These CIMs can be synthesized and released by activated immune cells, thereby regulating and promoting the inflammatory process. They play essential roles in the immune system, modulating cell proliferation, mediating inflammation, and clearing pathogens, among other physiological and pathological processes. Nevertheless, excessive or abnormal production of CIMs may lead to chronic inflammatory diseases, such as rheumatoid arthritis, inflammatory bowel disease, and autoimmune diseases (9–13). Consequently, further research on CIMs is paramount for understanding the mechanisms of inflammation and developing treatment methods for related diseases. Various inflammatory mediators, including IL-1, IL-6, IL-8, TNF-α, and IL-12, are known to be involved in the onset and progression of osteoporosis (14). These mediators interact with proteins related to bone resorption, impairing the function of osteoblasts and ultimately leading to osteoporosis.

However, the relationship between the levels of CIMs and OP is not yet clear and may be related to the relatively high concentrations of these proteins in the body. Currently, some scholars believe that there is an association between CIMs and OP (9, 13), but the mechanisms underlying this association remain unclear. In recent years, Mendelian randomization (MR) has emerged as an innovative statistical method and has gained widespread attention in the fields of medicine and biology. This method uses genetic variations as instrumental variables (IVs) to assess the causal relationships between exposures and outcomes (15), thus offering new insights into many diseases that have traditionally been difficult to elucidate.

In this study, we performed bidirectional MR analysis to investigate the causal effects of CIMs on OP and vice versa. This may provide a theoretical basis for further elucidation of the causal relationship between them.

## 2 Materials and methods

### 2.1 Study design

**Figure 1:**
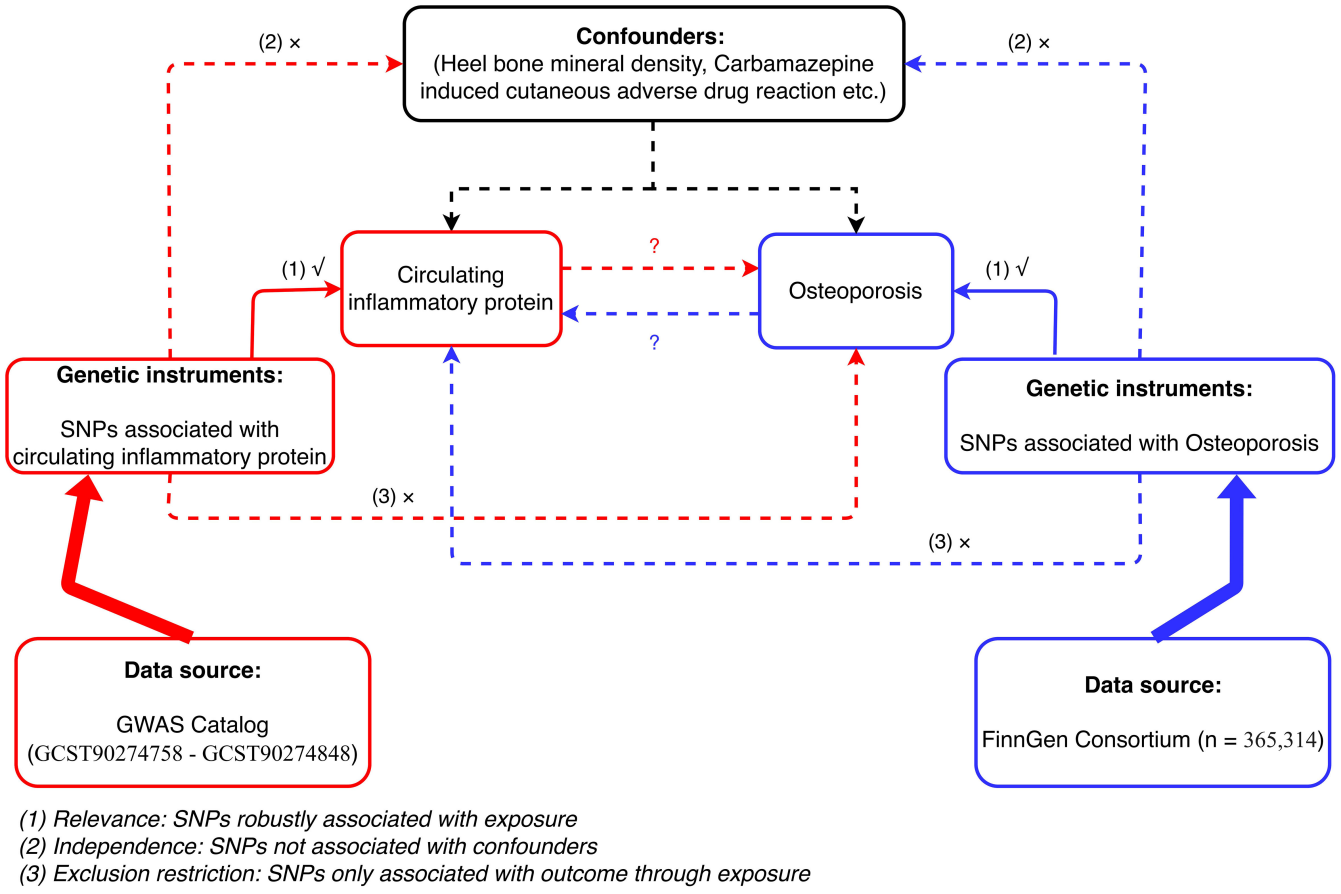
Flowchart of bidirectional MR analysis between CIMs and OP.

The MR analysis in our study was based on three assumptions: (1) the genetic instrumental variables (IVs) are strongly associated with exposure; (2) the selected genetic IVs are not associated with potential confounders; and (3) the genetic IVs can only affect the risk of outcome dependently through exposure (16). This bidirectional MR analysis was performed in two steps. First, CIMs were investigated as exposures, and OPs were investigated as outcomes in the first step. In the second step, this was reversed. **Figure 1** shows an overview of the three assumptions and study design. The confounders are listed in **Supplementary Table 1**.

### 2.2 Data sources

The summary-level data used in this study are deidentified public data and are available for download. Each GWAS involved in this study was approved by the ethics committee of the respective institution.

OP-related GWAS summary statistics were extracted from the FinnGen consortium, including data from 365,314 individuals of European ancestry and 7300 individuals with OP. More details for phenotype and modeling, genotype quality control, and related association analyses can be found on the FinnGen website (https://www.finngen.fi/en/) (17). Associations were tested after adjusting for covariates, including heel bone mineral density, heel bone mineral density left, heel bone mineral density right, bone density, lumbar spine bone mineral density, self-reported osteoporosis, and carbamazepine-induced cutaneous adverse drug reactions.

Summary statistics for CIMs were publicly available from the GWAS Catalog (accession numbers from GCST90274758 to GCST90274848) (http://ftp.ebi.ac.uk/pub/databases/gwas/summary_statistics/GCST90274001-GCST90275000). A total of 91 phenotypes were included. The original GWAS of immune traits was performed using data from European individuals, and there were no overlapping cohorts. The corresponding information for these 91 CIMs can be found in **Supplementary Table 2**.

### 2.3 Genetic instrumental variable (IV) selection

1. The IVs selected for analysis are highly related to the corresponding exposures, and we chose significant single nucleotide polymorphisms (SNPs) based on a loose cutoff of *p* < 1 × 10^-5^ to ensure sufficient IVs for screening.
2. The IVs are mutually independent and avoid the offset caused by linkage disequilibrium (LD) between the SNPs (r2 < 0.1, LD distance > 500 kb).
3. We eliminated IVs with an F-statistic <10 to minimize potential weak instrument bias F = R2 (n-k-1)/k (1-R2) (n is the sample size, k is the number of included IVs, and R2 is the exposure variance explained by the selected SNPs).

### 2.4 MR analysis

The inverse variance weighted (IVW), weighted median, weighted mode, simple mode, and MR−Egger methods were used to evaluate the bidirectional relationships between CIMs and OP as the main statistical approach (https://mrcieu.github.io/TwoSampleMR/). The IVW method was considered the most accurate method for estimating causal relationships if there was no clear evidence for the presence of directional pleiotropy (*p* for MR−Egger intercept > 0.05) (16, 18). When there was insufficient evidence of heterogeneity (*p* for MR heterogeneity > 0.05) in these selected genetic IVs, a random-effects model was used; otherwise, a fixed-effects model was used. A weighted median method was also used, which can generate effective causal estimates when at least 50% of the selected genetic IVs are valid (18). MR-PRESSO was used to test for pleiotropy and detect outliers. Considering multiple testing of OP and CIMs, we applied a moderate approach (false discovery rate, FDR) by adjusting the *p* values separately to correct for multiple hypothesis testing (19, 20). FDR *q*-values less than 0.3 were considered to indicate statistical significance.

### 2.5 Bayesian colocalization analysis

Bayesian colocalization analysis can evaluate the shared local genetic architecture between two traits and is valuable for further identifying MR associations caused by LD confounding (21). In this study, the “COLOC” package was used to perform Bayesian colocalization analysis (22). This package incorporates sophisticated algorithms and models to estimate the probability of colocalization between genes associated with the two traits under investigation. To determine whether colocalization occurred, a threshold was set based on previous studies. Specifically, PPH4 represents the probability that both traits share the same causal variant. If the posterior probability of hypothesis 4 (PPH4) exceeded 80%, it was considered significant evidence supporting the colocalization of genes (23).

### 2.6 Statistical analysis

To assess the causal relationship between OP and 91 CIMs, the “Mendelian-Randomization” package (version 0.4.3) (24) was primarily used to carry out the IVW (25), weighted median-based (18), and mode-based methods (26). To test for heterogeneity among certain IVs, Cochran’s Q statistic and related p values were used. Random effects IVW, as opposed to fixed effects IVW, was employed in the event that the null hypothesis was rejected (25). MR−Egger, a widely used approach that assumes the presence of horizontal multiplicity if its intercept term is large, was employed to eliminate the effect of horizontal pleiotropy (27). Moreover, a potent technique known as the MR pleiotropy residual sum and outlier (MR-PRESSO) method was applied to exclude any potential horizontal pleiotropic outliers that might have a significant impact on the estimation outcomes within the MR-PRESSO package (28). Furthermore, funnel plots and scatter plots were generated. Scatter plots demonstrated that outliers had no effect on the results. Funnel plots showed that there was no heterogeneity, and the correlation was robust. The bidirectional MR effect between each CIM and OP and its corresponding SNPs can be found in the **Supplementary Material** named dat.csv. The positions of the valid SNPs for OP and each CIM in the context of the annotation can be accessed in the sheet corresponding to the CIM accession ID, with the suffix csv-dat.xlsx.

## 3 Results

### 3.1 Exploration of the causal effect of CIMs on OP onset

To explore the causal impact of CIMs on OP, two-sample MR analysis was performed. The threshold for statistical significance was set as an FDR below 0.3, and the quantity of SNPs for each metric was considered to augment the robustness of the genetic IVs. All these genetic IVs met the requirements of linkage disequilibrium (LD)-independent (r2 < 0.1) and achieved a genome-wide significance level (*p* < 1 × 10^−5^).

**Figure 2:**
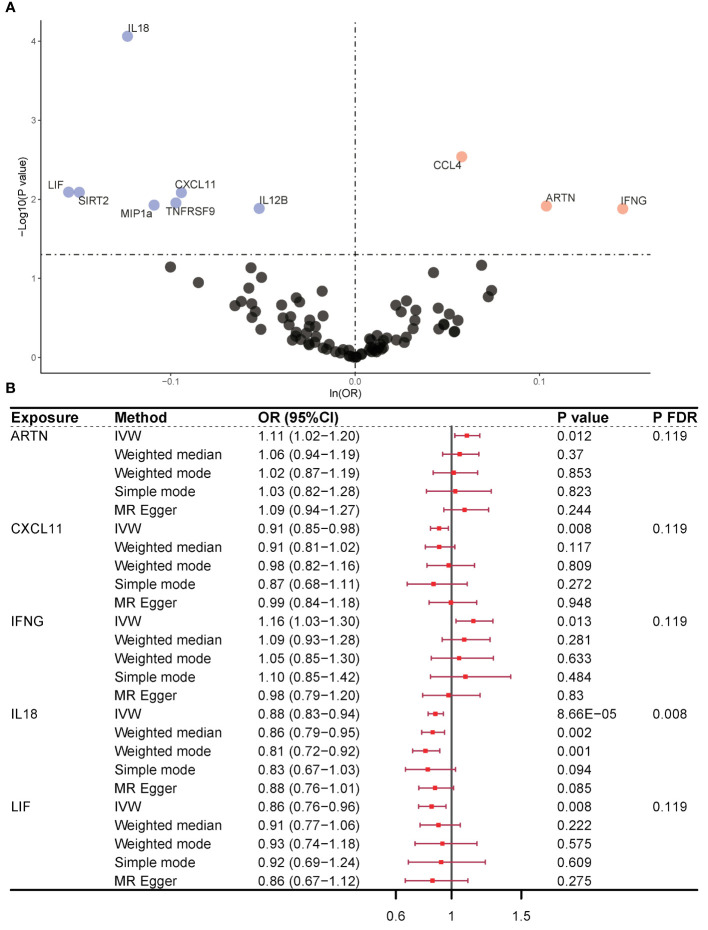
Causality outcomes for CIMs and OP. **(A)** Volcano plot showing the association between various CIMs and OP risk. Significant CIMs are labeled. **(B)** Forest plot presenting ORs with 95% CIs for selected CIMs using different MR methods. Five CIMs were identified as being associated with OP after MR analysis. ARTN and IFNG were risk factors for OP. CXCL11, IL-18, and LIF were identified as protective factors against OP. CIM, circulating inflammatory marker; OP, osteoporosis; MR, Mendelian randomization; OR, odds ratio; FDR, false discovery rate; nsnp, number of single nucleotide polymorphisms; ARTN, Artemin; IFNG, interferon gamma; CXCL11, chemokine (C-X-C motif) ligand 11; IL-18, interleukin 18; LIF, leukemia inhibitory factor.

In our examination of CIMs as an exposure variable, we employed a multitude of SNPs as instrumental variables to strengthen our analysis. The IVW approach indicated a statistically significant association between 10 CIMs and the occurrence of OP, and the number of CIMs was reduced to five after eliminating horizontal pleiotropy of variables by MR-PRESSO. Only three CIMs were found to play a protective role in OP pathogenesis, including 11 SNPs for chemokine (C-X-C motif) ligand 11 (CXCL11) (IVW, OR = 0.91, 95% CI = 0.85–0.98, P = 0.008, P_FDR_ = 0.119), 58 SNPs for interleukin 18 (IL-18) (IVW, OR = 0.88, 95% CI = 0.83–0.94, P = 8.66×10^-5^, P_FDR_ = 0.008), and 24 SNPs for leukemia inhibitory factor (LIF) (IVW, OR = 0.86, 95% CI = 0.76–0.96, P = 0.008, P_FDR_ = 0.119). Two CIMs were found to be risk factors for the pathogenesis of OP, including 34 SNPs in artemisinin (ARTN) (IVW, OR = 1.11, 95% CI = 1.02–1.20, P = 0.012, P_FDR_ = 0.119) and 18 SNPs in interferon gamma (IFNG) (IVW, OR = 1.16, 95% CI = 1.03–1.30, P = 0.013, P_FDR_ = 0.119) (**Figure 2**). The causal effects of all five CIMs on OP are listed in **Supplementary Table 3**.

### 3.2 Exploration of the causal effect of OP onset on CIMs

**Figure 3:**
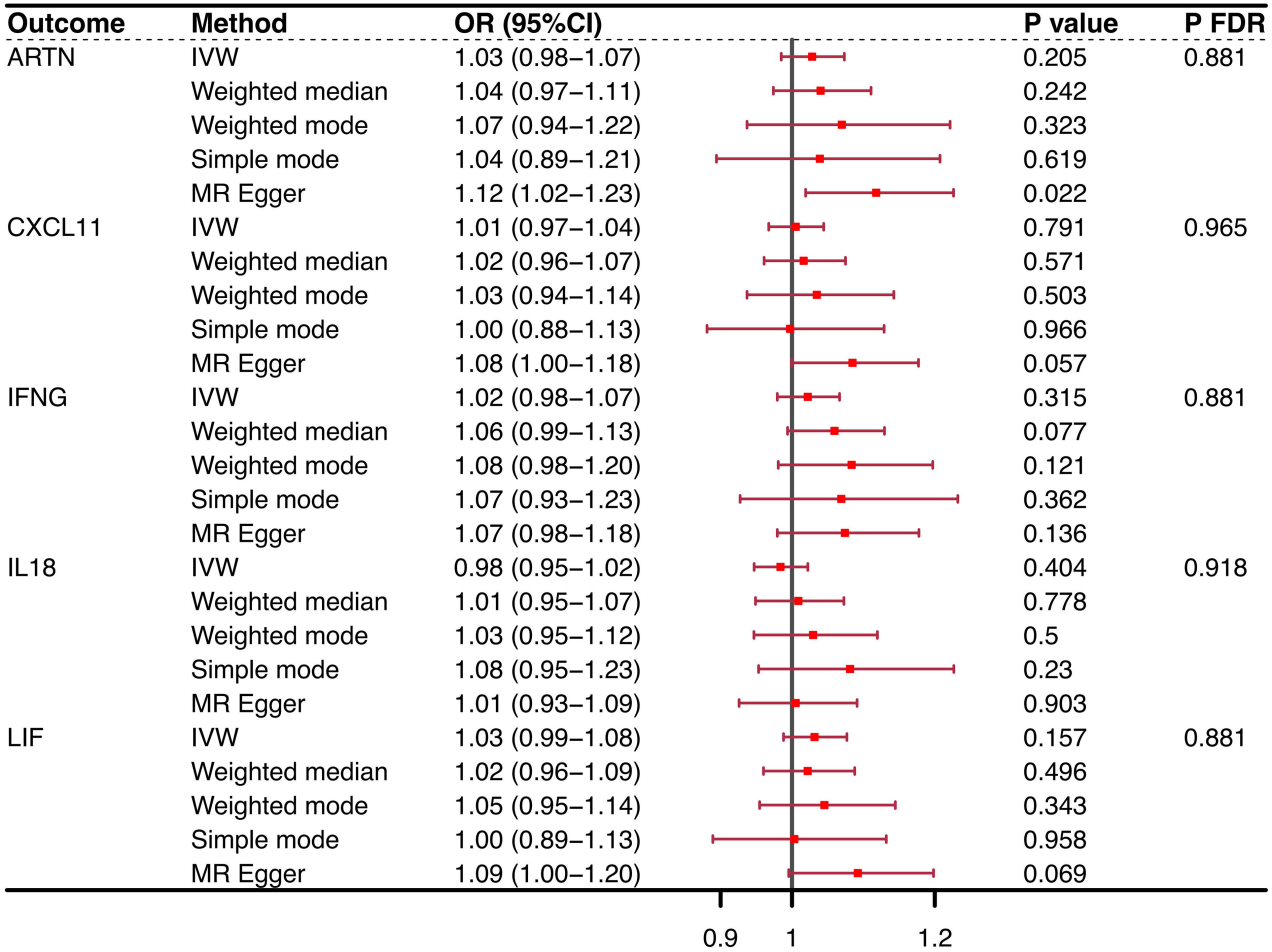
Reverse causality outcomes for CIMs and OP. After MR analysis, no CIM achieved statistical prominence when OP was the exposure variable. None of the PFDRs were less than 0.3. CIM, circulating inflammatory marker; OP, osteoporosis; MR, Mendelian randomization; OR, odds ratio; FDR, false discovery rate; nsnp, number of single nucleotide polymorphisms; ARTN, Artemin; IFNG, interferon gamma; CXCL11, chemokine (C-X-C motif) ligand 11; IL-18, interleukin 18; LIF, leukemia inhibitory factor.

Conversely, when considering OP as the exposure variable, no CIM achieved statistical prominence in the conventional purview [ARTN (IVW, OR = 1.03, 95% CI = 0.98–1.07, P = 0.205, P_FDR_ = 0.881), CXCL11 (IVW, OR = 1.01, 95% CI = 0.97–1.04, P = 0.791, P_FDR_ = 0.965), IFNG (IVW, OR = 1.02, 95% CI = 0.98–1.07, P = 0.315, P_FDR_ = 0.881), IL-18 (IVW, OR = 0.98, 95% CI = 0.95–1.02, P = 0.404, P_FDR_ = 0.918), LIF (IVW, OR = 1.03, 95% CI = 0.99–1.08, P = 0.157, P_FDR_ = 0.881)] (**Figure 3**). However, significant linkages might not be precluded under the more accommodating FDR benchmarks of the present study. Notably, the MR−Egger intercept and the global test from MR-PRESSO discounted the possibility of horizontal pleiotropy, thereby strengthening the validity of our results. The consistency of these findings was also supported by scatter plots and funnel plots, which indicated the reliability of our conclusions regarding the relationship between CIMs and OP. The results of the reverse causal effect of OP on all CIMs are listed in **Supplementary Table 4**.

### 3.3 Bayesian colocalization analysis of CIMs and OP onset

Using Bayesian colocalization analysis, we examined the associations between several CIMs (ARTN, CCL11, IFGN, IL18, and LIF) as exposure factors and OP as an outcome. We found no evidence to support the presence of shared causal variants using COLOC analysis in the training and testing cohorts (ARTN: PPH4  = 0.030; CCXL11: PPH4  = 0.052; IFGN: PPH4  = 8.23e×10–6; IL-18: PPH4  =0.042; LIF: PPH4  =0.005) for the association between CIMs and OP (rs17490485, ARTN; rs4859679, CCXL11; rs438211, IFNG; rs735622, IL-18; rs116967415, LIF). The regional colocalization plots for these associations and the detailed results of the shared genetic IVs and located genes are listed in **Supplementary Table 5**; **Supplementary Figure 1**.

## 4 Discussion

In our study, we performed bidirectional Mendelian randomization to investigate the potential causal associations between OP and 91 CIMs. Based on relatively large publicly available GWAS meta-analyses, we found positive unidirectional causal associations between CIMs and OP. Five CIMs (ARTN, CXCL11, IL-18, LIF, and IFNG) demonstrated a potential association with OP, and OP occurrence was not associated with an alteration in CIMs. These unidirectional associations were consistent with the sensitivity analyses but not supported by the colocalization analyses.

After MR analysis, we identified three protective factors for OP: CXCL11, LIF and IL-18.

CXCL11 is a small cytokine of the CXC chemokine family and has been shown to inhibit osteoclast differentiation of CD14+ monocytes (29). In our study, CXCL11 was shown to play a protective role in OP pathogenesis. The potent inhibition of osteoclastogenesis by IFN-β is partly mediated by the chemokine CXCL11. Similarly, our study revealed that CXCL11 plays a protective role in the pathogenesis of OP.

Several studies have shown a role for LIF in stimulating bone formation *in vivo* (30, 31). The expression level of LIF mRNA is increased upon osteogenic differentiation, resulting in LIF production by osteoblasts (32). Bozec et al. showed a 40% decrease in bone volume in newborn LIF-mutant animals. These animals did not have altered osteoblasts but demonstrated significant increases in osteoclast number and size, relative osteoclast surface area, and bone resorption (33). These findings suggest that LIF prevents OP by controlling osteoclast survival and size and explains why LIF was a protective factor against OP in our study.

IL-18 demonstrated a protective effect against osteoporosis in our study, possibly because IL-18 promotes osteogenic differentiation of hBMSCs through the SLC7A5/c-MYC pathway. SLC7A5 and c-MYC play important roles in the IL-18-induced expression of osteogenic markers in hBMSCs. IL-18 upregulates the expression of SLC7A5 and c-MYC at the early stage of hBMSC osteogenic differentiation, and SLC7A5 and c-MYC inhibition blocks the osteogenic differentiation induced by IL-18 (34). The bone density of elderly patients with osteoporosis increases significantly after anti-osteoporosis treatment. This may be related to the ability of IL-18 to inhibit osteoclast activity, induce the proliferation and differentiation of bone marrow-derived lymphoid progenitor cells, and promote NK cell proliferation and cytotoxicity (35, 36).

We also found two risk factors associated with OP by MR analysis: ARTN and IFNG.

As a member of the glial cell-derived neurotrophic factor (GDNF) ligand family, the major function of ARTN is to drive the molecule to induce sympathetic neuron migration and axon projection (37), and ARTN has been implicated in pain signaling, including that derived from the joint and bone (38–40). Some studies suggest that ARTN/GFRα3 signaling is involved in the pathogenesis of bone pain, and inhibition of this process could be used to treat pain in osteoarthritis (OA) when pathological features are present in the subchondral bone (38, 39). Other studies have suggested that ARTN is regulated by estrogen and mediates estrogen resistance in breast cancer (41, 42). It has been well documented that estrogen is closely related to osteoporosis, especially postmenopausal osteoporosis in women (43–45). In bone, estrogen inhibits osteoclast formation and bone resorption activity by binding to the estrogen receptor, promoting osteoprotegerin (OPG) expression, and inhibiting the action of nuclear factor-κβ ligand (RANKL). However, to date, research on the relationship between ARTN and OP remains relatively limited, and there is currently no conclusive evidence of a direct association between ARTN and OP. Our study is the first to find that ARTN is positively correlated with the risk of OP from a genetic point of view.

INFG is a classic proinflammatory mediator that is overexpressed in the course of OP (46). Comprehensive studies have confirmed that circulating IFNG levels are significantly elevated in patients with OP and that their upregulation also correlates with a severe OP phenotype (47, 48). This finding is consistent with the results of our study, which revealed that IFNG is a risk factor for OP.

Previous studies have focused on disease-to-disease or symptom-to-disease relationships. To our knowledge, no MR analysis focused on the causal relationship between OP and multiple CIMs has yet been reported. Our study used several variants summarized from large-scale GWASs on OP and CIMs to increase the statistical power to detect causal associations. This approach extends the findings of previous studies. Moreover, this approach can be applied to identify potential novel molecular pathways relevant to the diagnosis and treatment of OP, as the pathophysiological mechanisms of this relationship have not yet been fully elucidated. Therefore, we performed a colocalization analysis to identify five causal variants and localize them to their respective genes. Current evidence that the five CIMs mentioned above (ARTN, CXCL11, IFGN, IL-18, and LIF) are involved in the pathogenesis of CIMs and OP is inconclusive. Thus, further in-depth studies are needed.

Our study has limitations. First, although we obtained positive sensitivity analysis results in MR analysis, colocalization analysis suggested that the association between CIMs and OP remained uncertain. Second, our MR analysis suggests a potential causal link between CIMs and OP risk, but this does not confirm direct causality. MR results rely on the assumption that the genetic variant is randomly associated with both CIMs and OP and is not influenced by confounders. Violations of these assumptions may bias the findings. Due to MR design limitations, we lack specific biomarker-level data. These results should be interpreted alongside other study evidence. Recognizing these limitations, our study provides a hypothesis that can guide future research investigating the specific levels and biological roles of these biomarkers in relation to OP risk. Third, because we used summary-level MR data, we were unable to perform subgroup-specific analysis.

## 5 Conclusion

In conclusion, we found unidirectional causal relationships between CIMs, including ARTN, CXCL11, IFGN, IL-18, and LIF, and OP in our MR sensitivity analysis, and a potential association between ARTN and OP was proposed for the first time. This association was not supported by colocalization analysis but still has great value for further in-depth study.

## Data availability statement

The datasets presented in this study can be found in online repositories. The names of the repository/repositories and accession number(s) can be found in the article/**Supplementary Material**.

## Author contributions

QD: Writing – review & editing, Writing – original draft, Visualization, Validation, Software, Resources, Project administration, Methodology, Formal analysis, Data curation, Conceptualization. JW: Writing – review & editing, Writing – original draft, Visualization, Validation, Methodology, Data curation, Conceptualization. HZ: Writing – review & editing, Visualization, Writing – original draft, Formal analysis, Data curation. LL: Writing – review & editing, Validation, Supervision, Resources, Project administration, Investigation, Formal analysis, Conceptualization. WW: Writing – review & editing, Validation, Supervision, Software, Resources, Project administration, Methodology, Investigation, Funding acquisition, Formal analysis, Conceptualization.
