# Supplementary figures and images for: The causal role of circulating inflammatory markers in osteoporosis: a bidirectional Mendelian randomized study
Source: Front Immunol. 2024 Jul 18;15:1412298. doi: 10.3389/fimmu.2024.1412298 (PMC11291241; doi:10.3389/fimmu.2024.1412298)

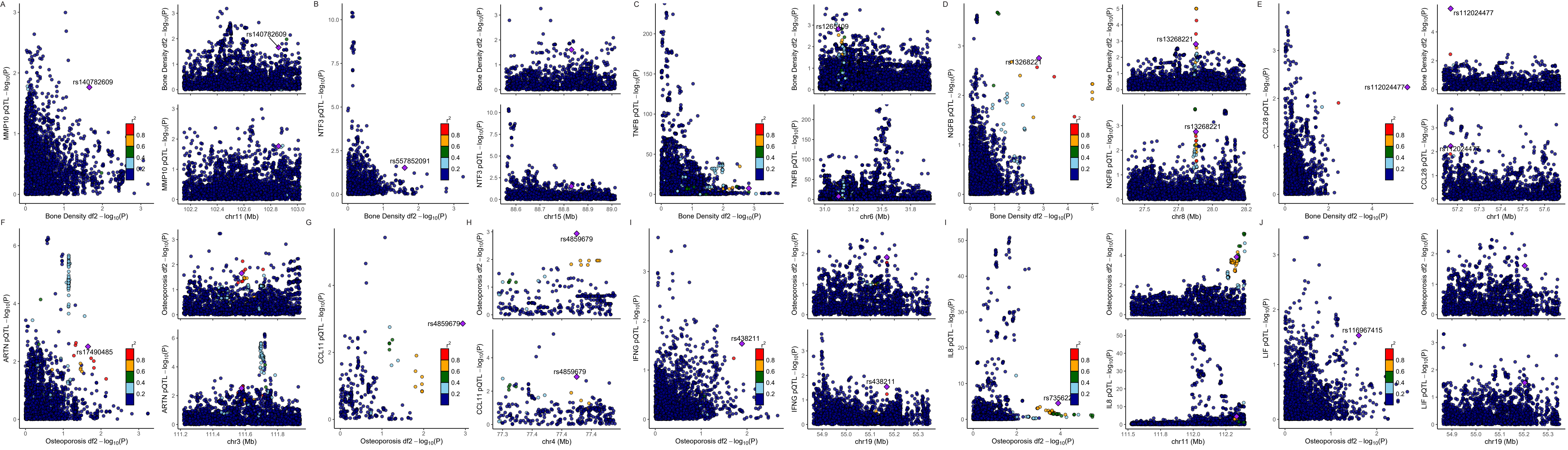

Supplement: Supplementary file 1 [file DataSheet_1.zip › supplementary material/Supplementary figure 1.pdf]

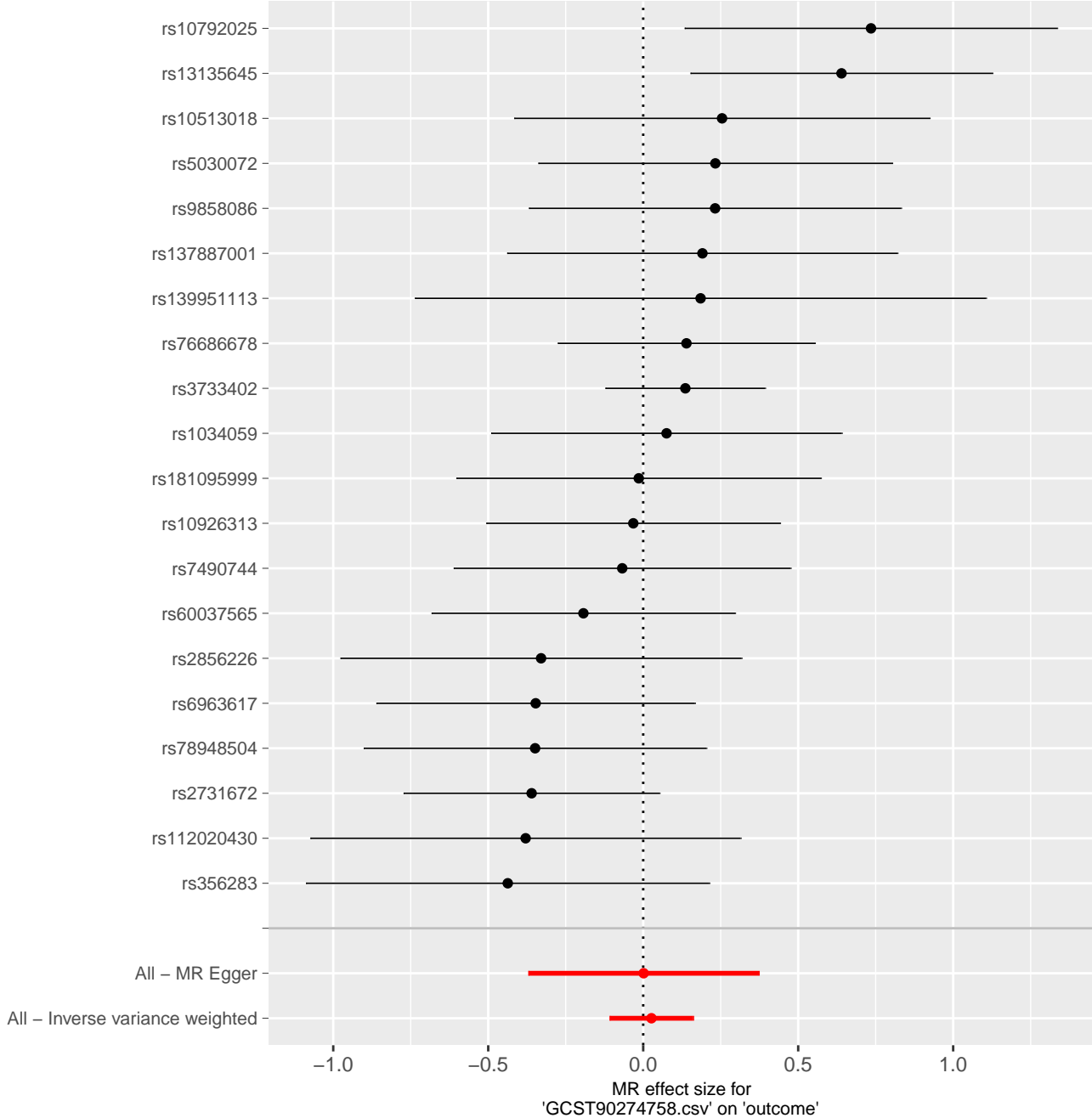

Supplement: Supplementary file 1 [file DataSheet_1.zip › supplementary material/CIM-OSTEOPOROSIS /GCST90274758.csv_forest.pdf]

# MR Method

- Inverse variance weighted
- MR Egger

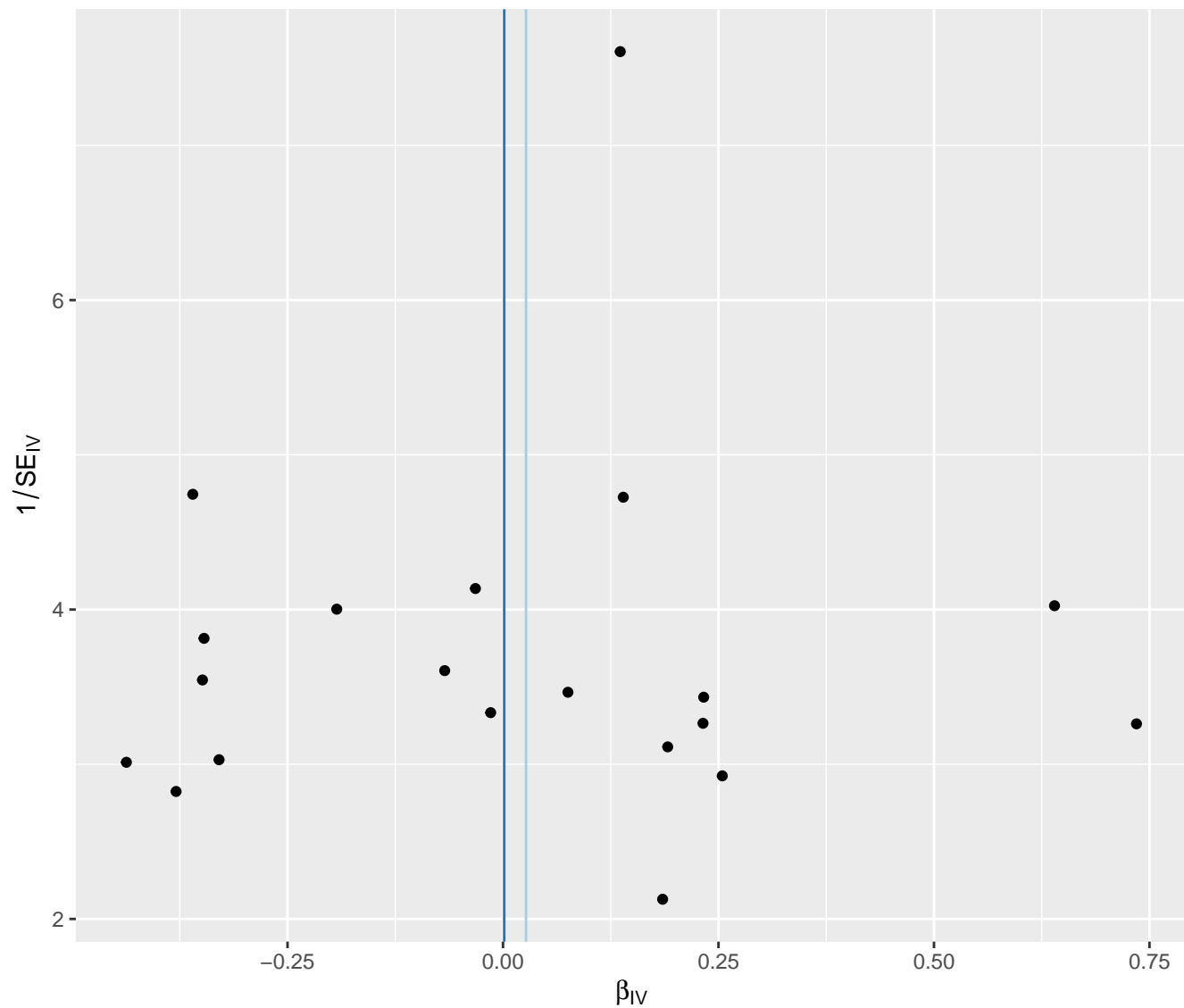

Supplement: Supplementary file 1 [file DataSheet_1.zip › supplementary material/CIM-OSTEOPOROSIS /GCST90274758.csv_funnel.pdf]

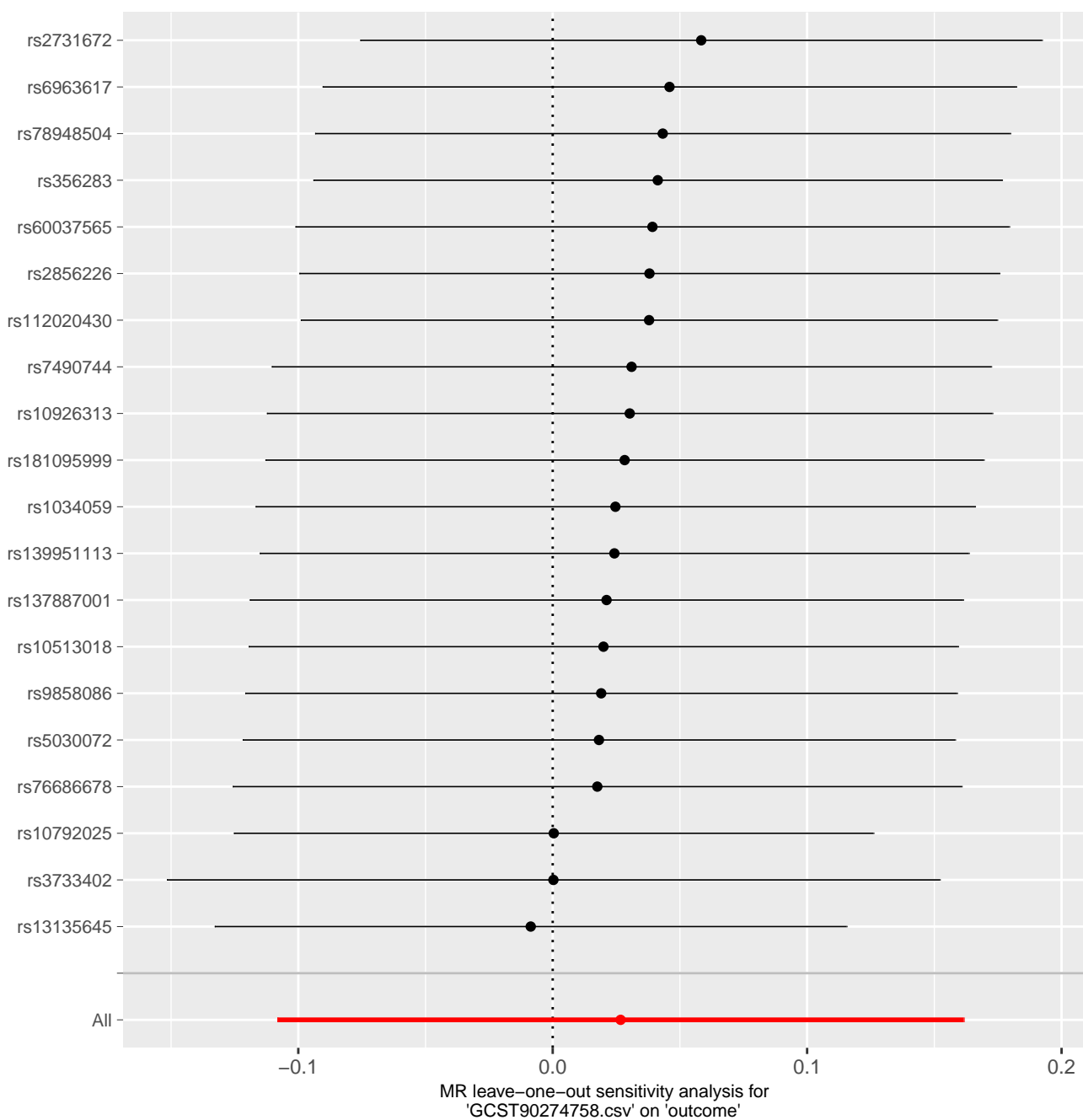

Supplement: Supplementary file 1 [file DataSheet_1.zip › supplementary material/CIM-OSTEOPOROSIS /GCST90274758.csv_leave_one_out.pdf]

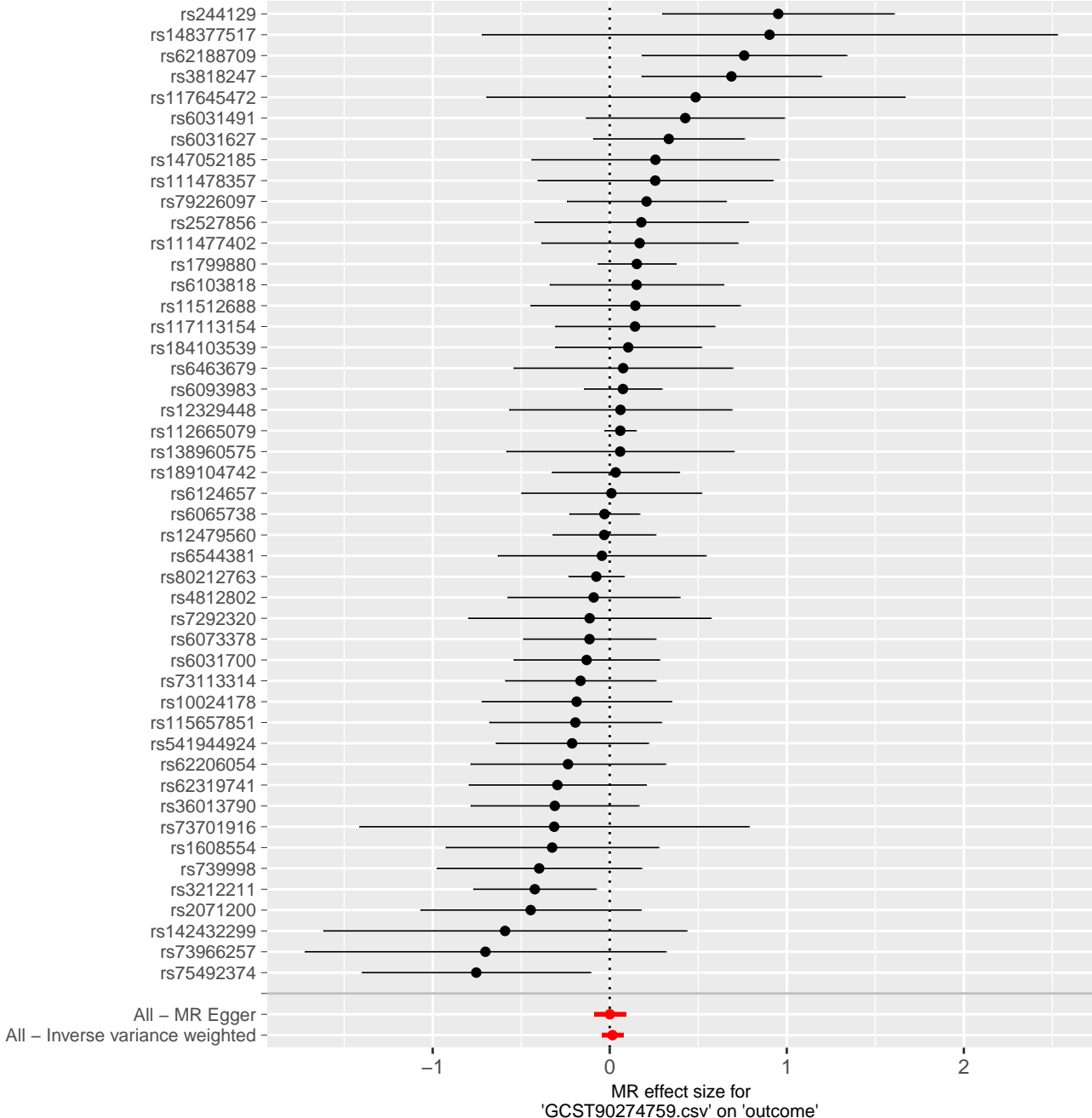

Supplement: Supplementary file 1 [file DataSheet_1.zip › supplementary material/CIM-OSTEOPOROSIS /GCST90274759.csv_forest.pdf]

# MR Method

- Inverse variance weighted
- MR Egger

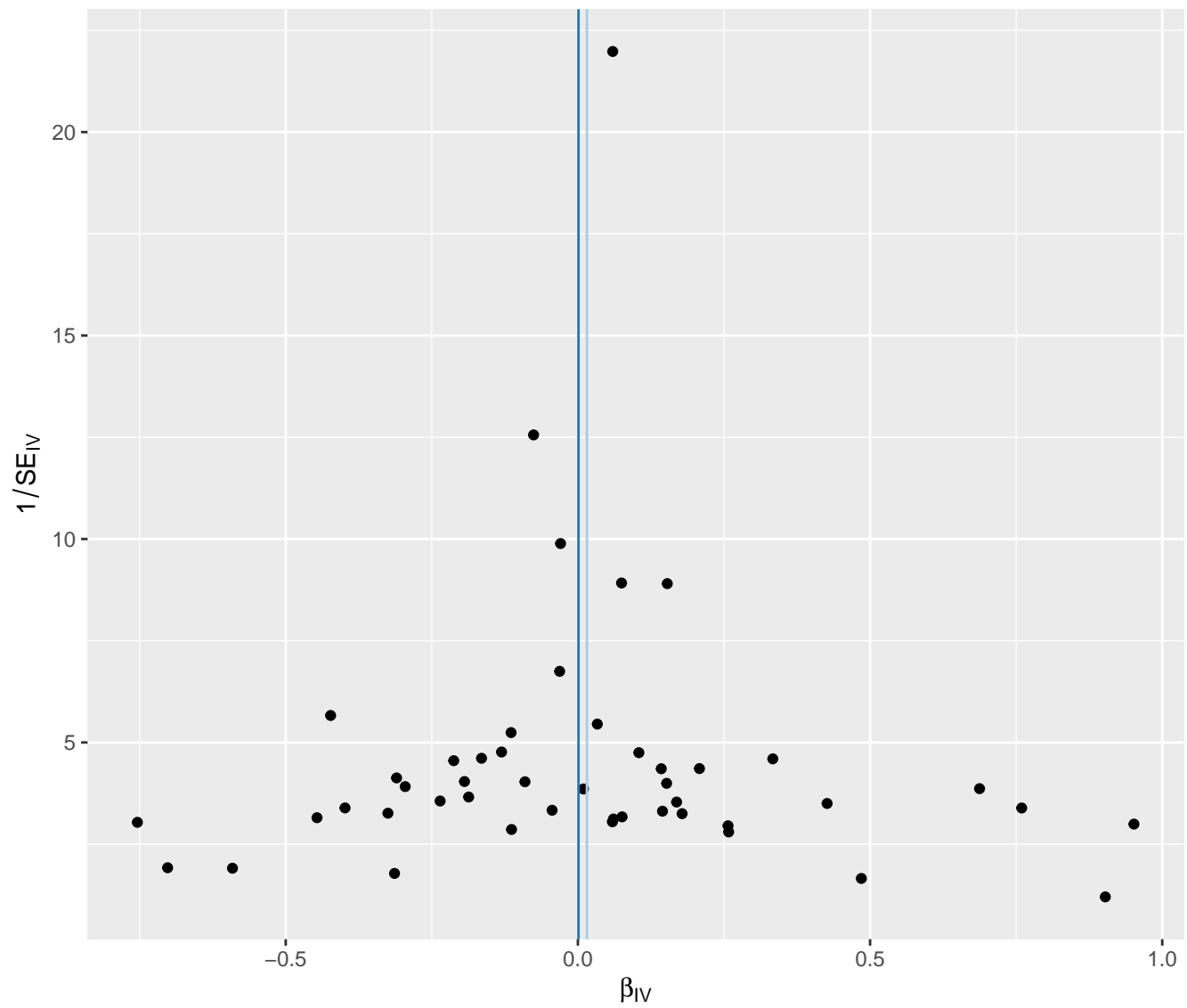

Supplement: Supplementary file 1 [file DataSheet_1.zip › supplementary material/CIM-OSTEOPOROSIS /GCST90274759.csv_funnel.pdf]

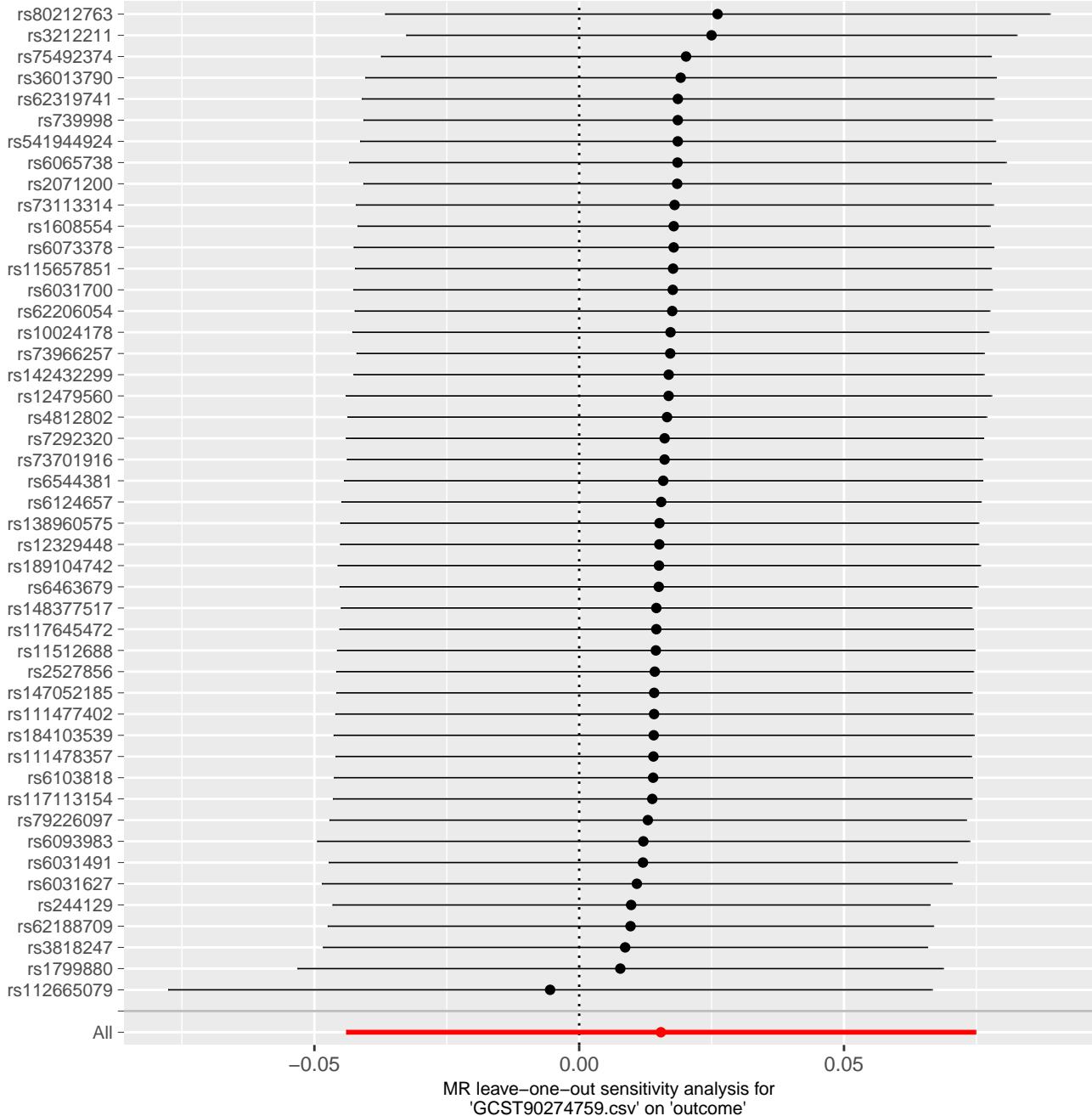

Supplement: Supplementary file 1 [file DataSheet_1.zip › supplementary material/CIM-OSTEOPOROSIS /GCST90274759.csv_leave_one_out.pdf]

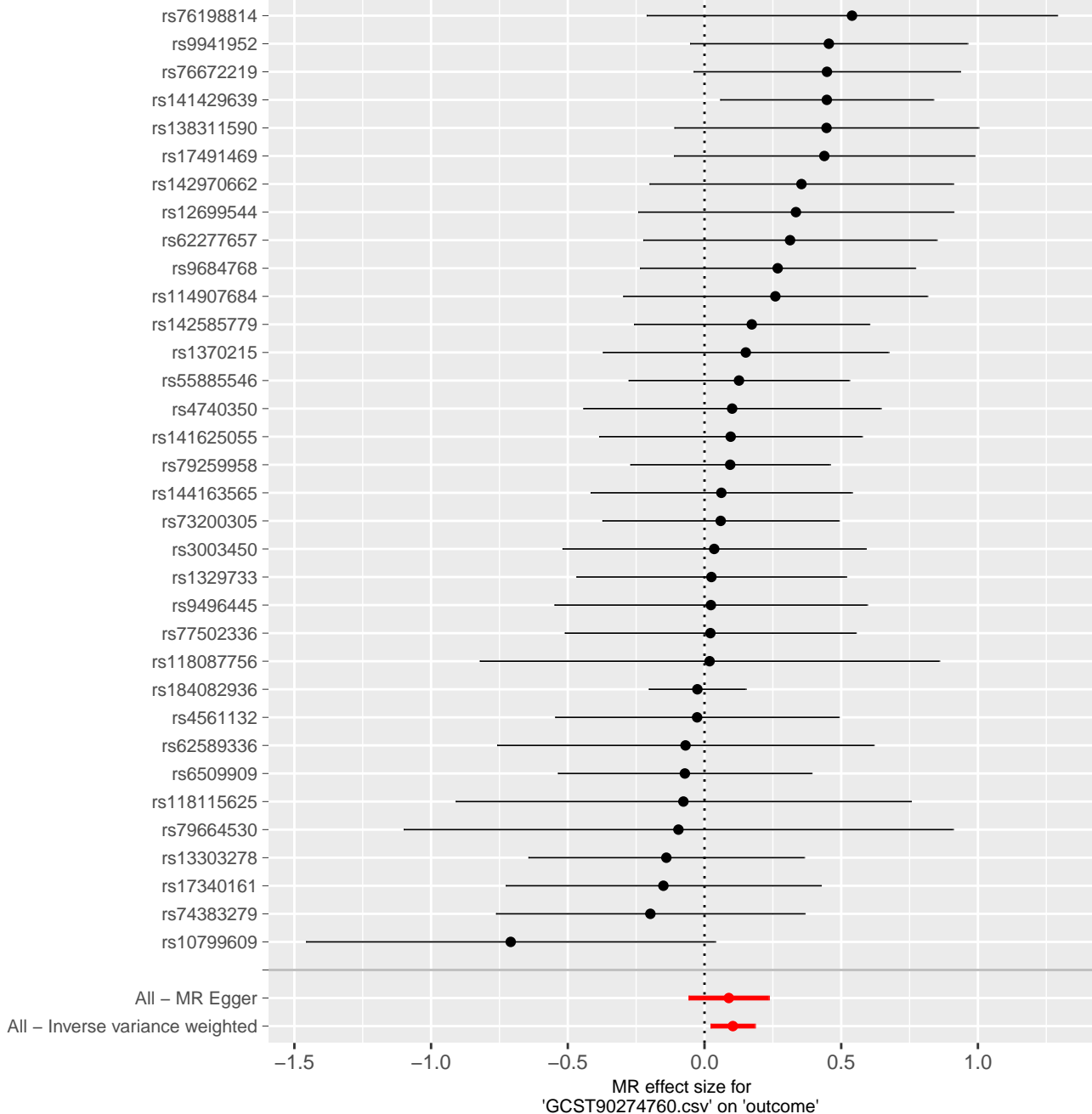

Supplement: Supplementary file 1 [file DataSheet_1.zip › supplementary material/CIM-OSTEOPOROSIS /GCST90274760.csv_forest.pdf]

# MR Method

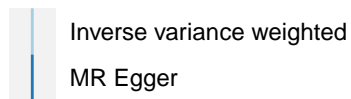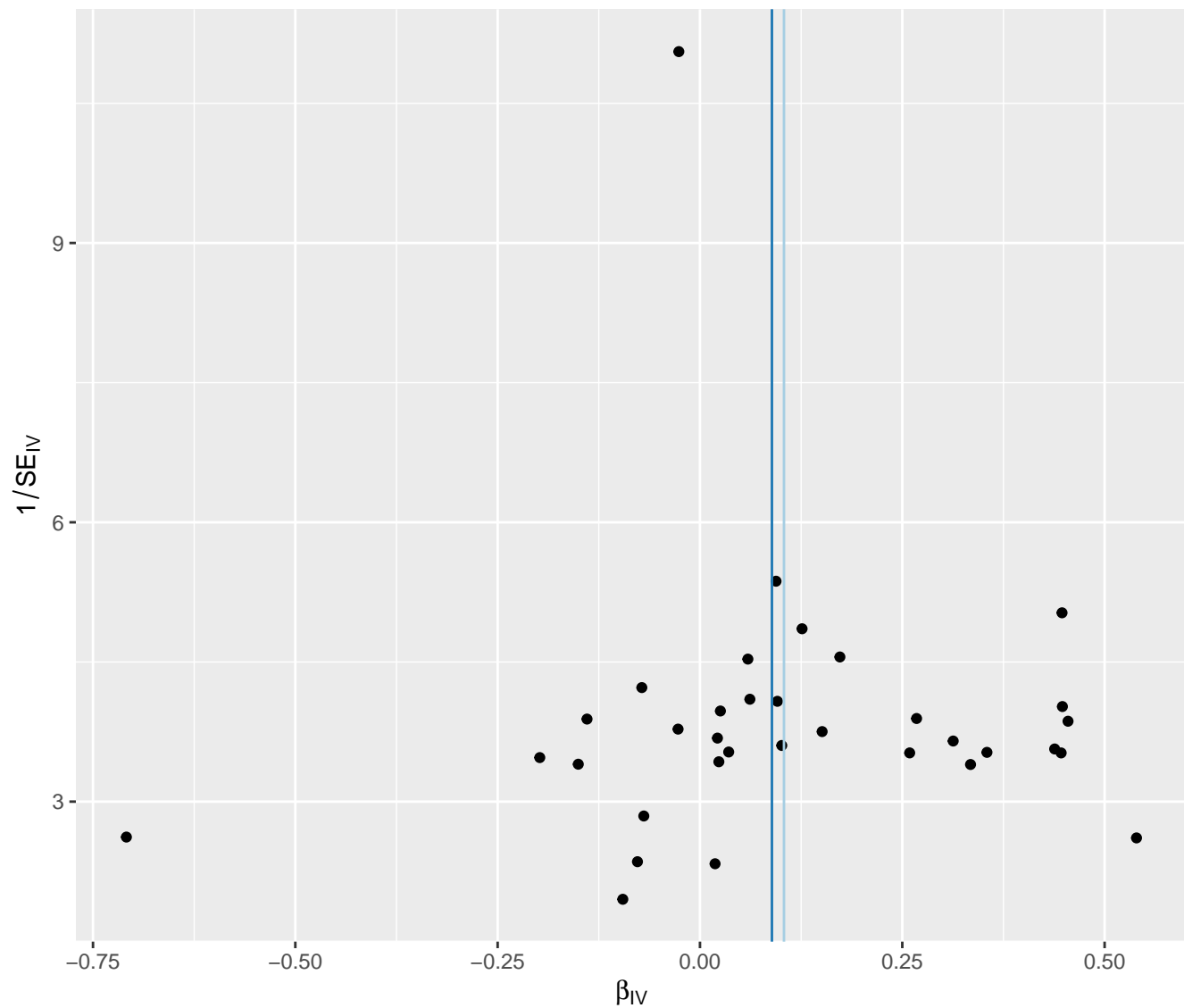

Supplement: Supplementary file 1 [file DataSheet_1.zip › supplementary material/CIM-OSTEOPOROSIS /GCST90274760.csv_funnel.pdf]

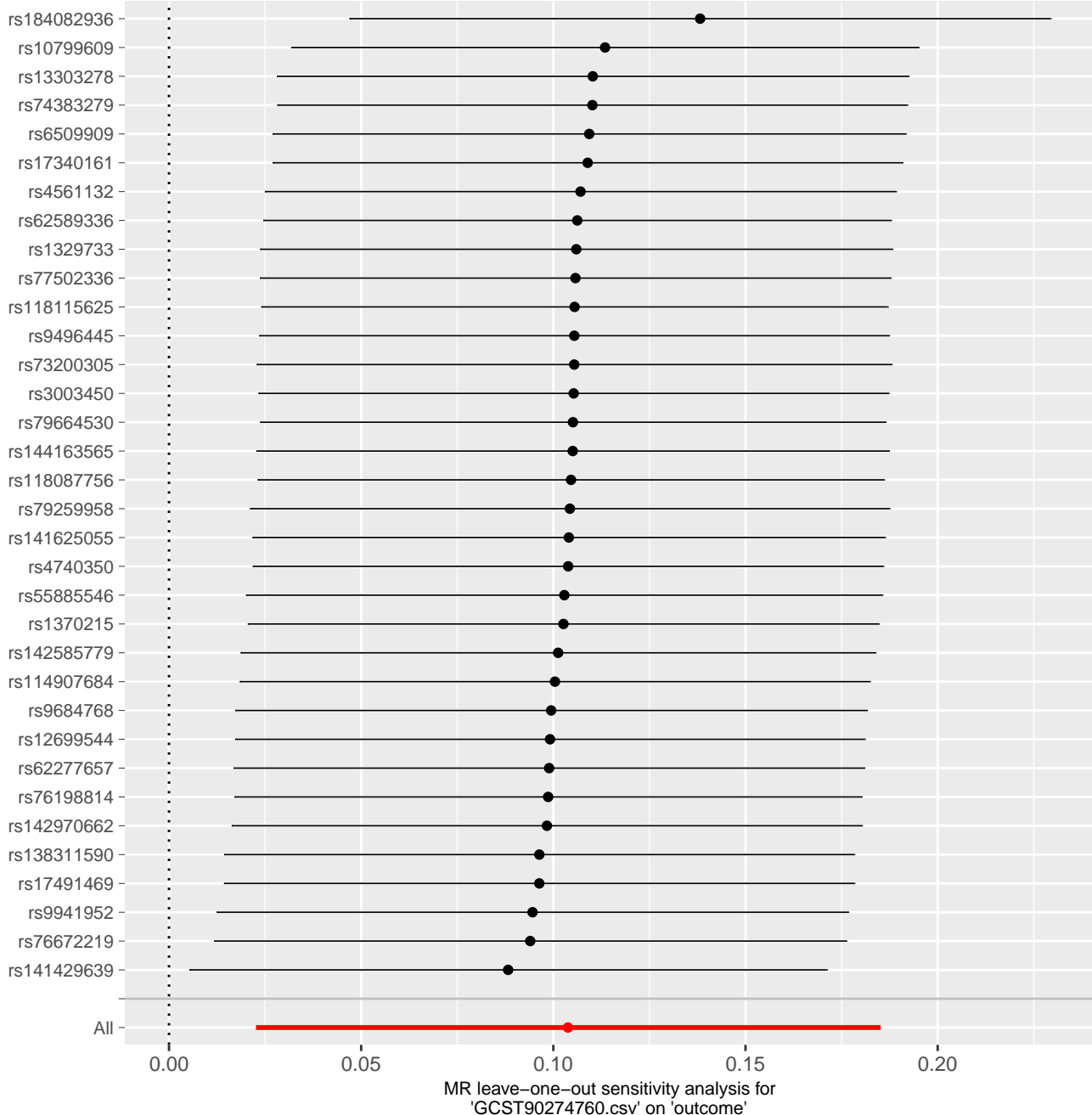

Supplement: Supplementary file 1 [file DataSheet_1.zip › supplementary material/CIM-OSTEOPOROSIS /GCST90274760.csv_leave_one_out.pdf]

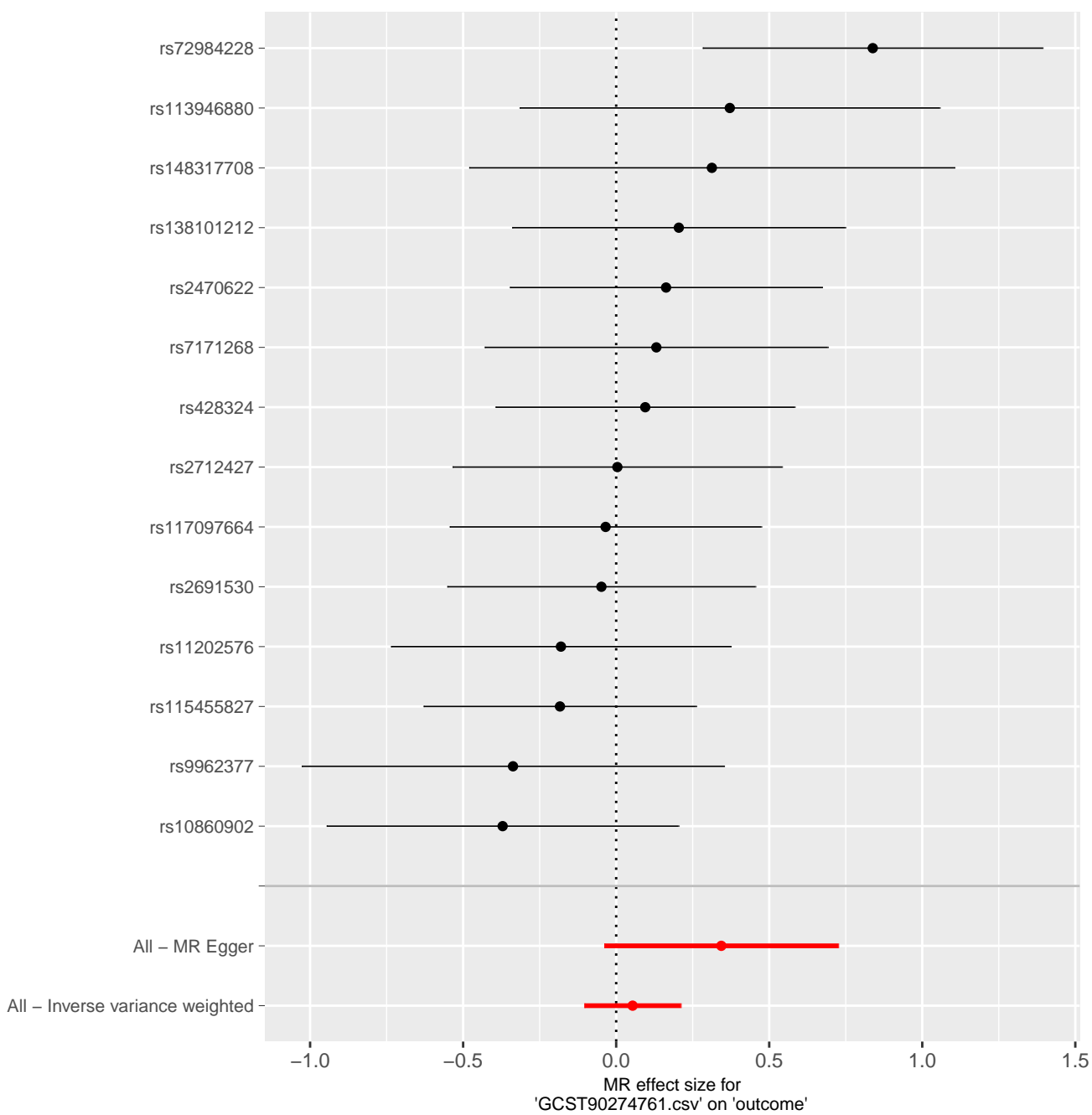

Supplement: Supplementary file 1 [file DataSheet_1.zip › supplementary material/CIM-OSTEOPOROSIS /GCST90274761.csv_forest.pdf]

# MR Method

- Inverse variance weighted
- MR Egger

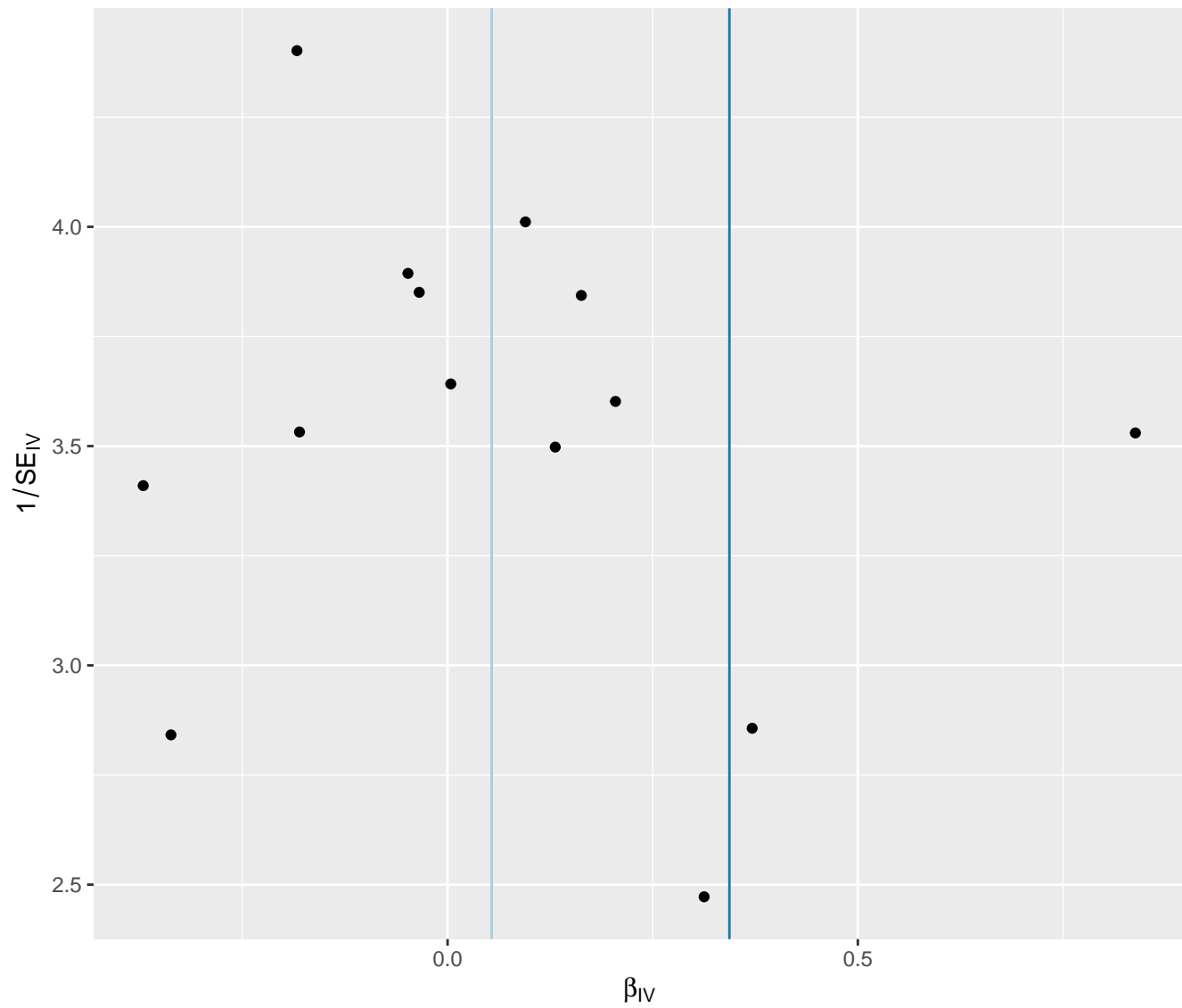

Supplement: Supplementary file 1 [file DataSheet_1.zip › supplementary material/CIM-OSTEOPOROSIS /GCST90274761.csv_funnel.pdf]

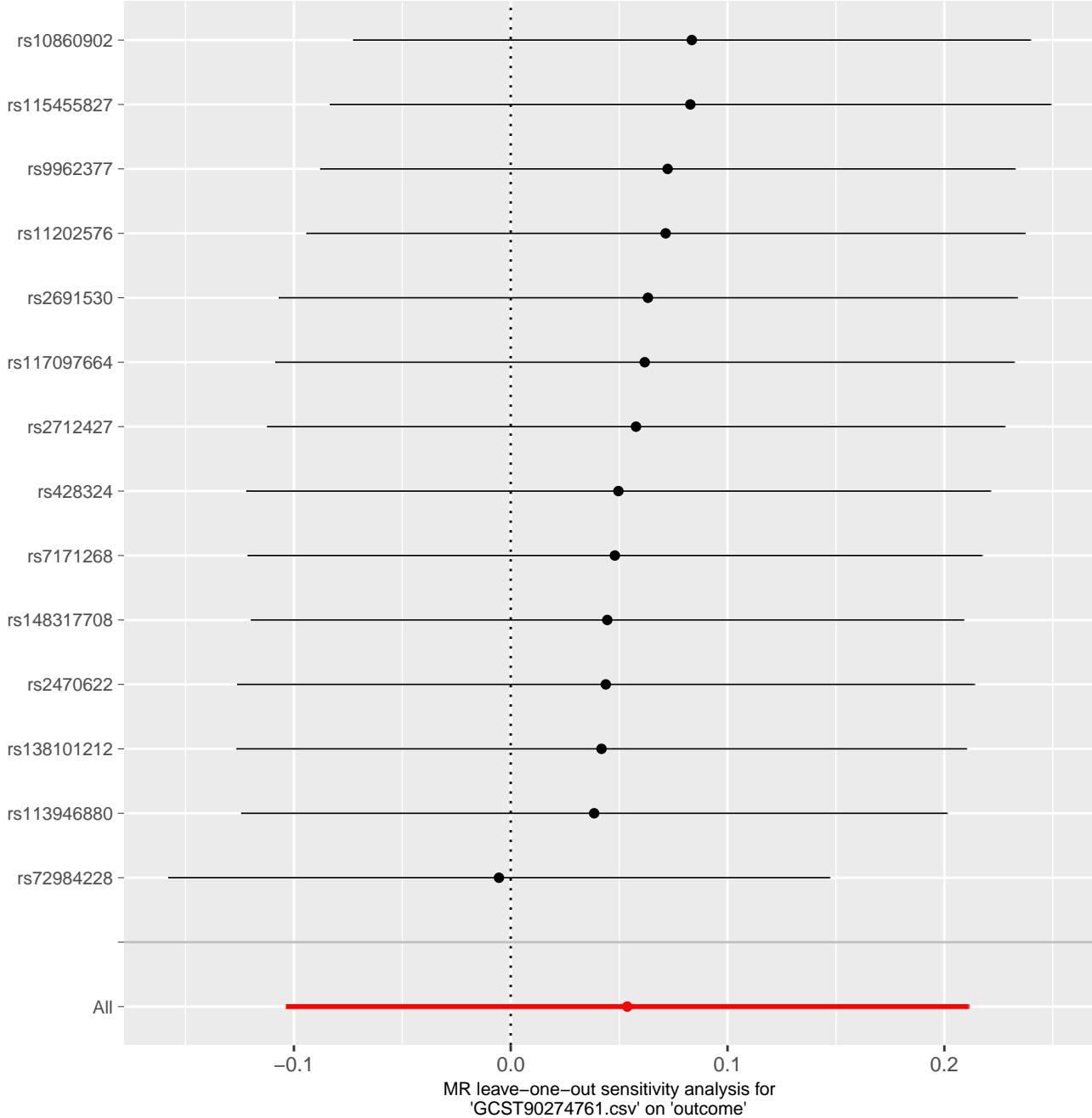

Supplement: Supplementary file 1 [file DataSheet_1.zip › supplementary material/CIM-OSTEOPOROSIS /GCST90274761.csv_leave_one_out.pdf]

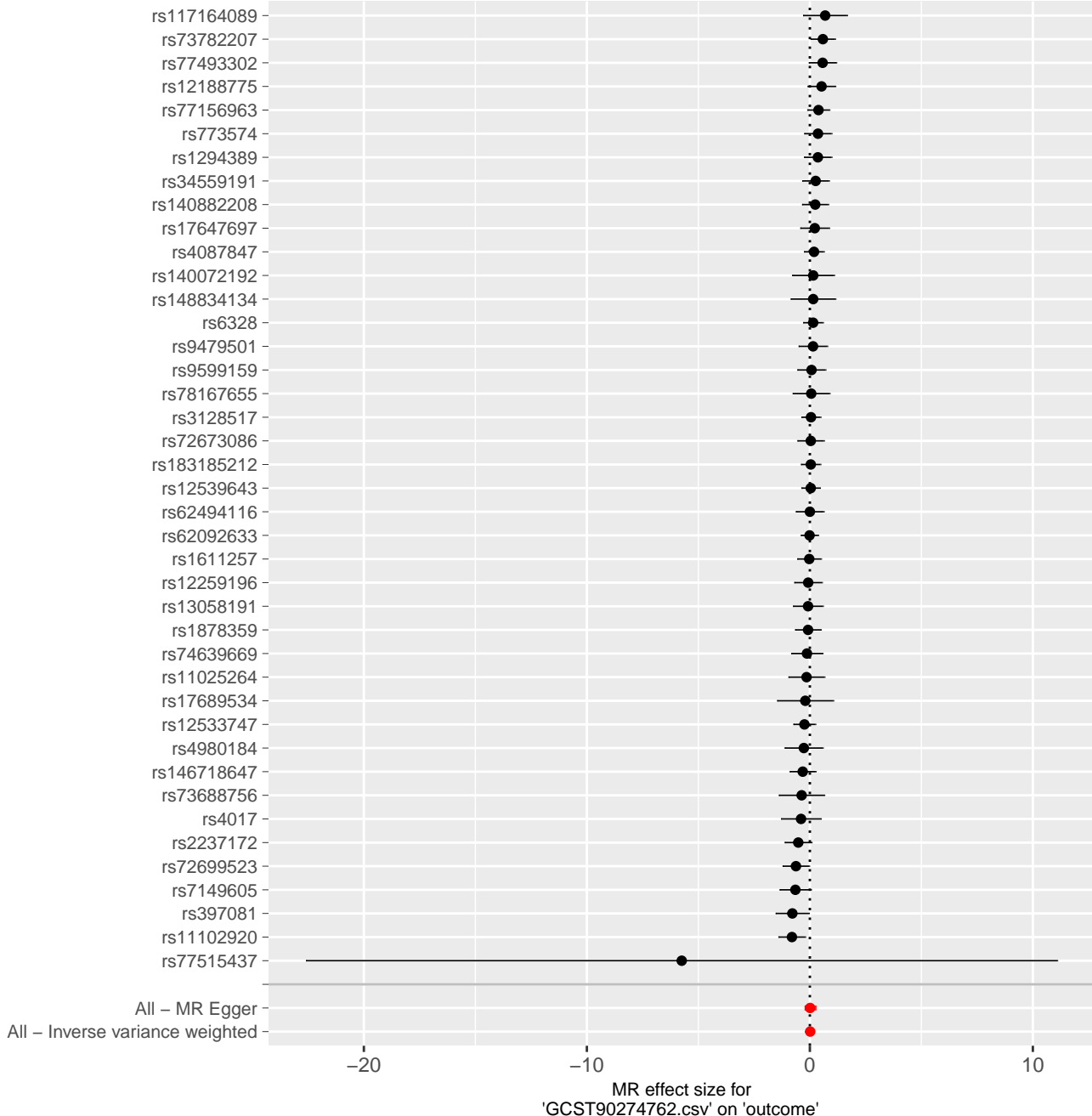

Supplement: Supplementary file 1 [file DataSheet_1.zip › supplementary material/CIM-OSTEOPOROSIS /GCST90274762.csv_forest.pdf]

# MR Method

- Inverse variance weighted
- MR Egger

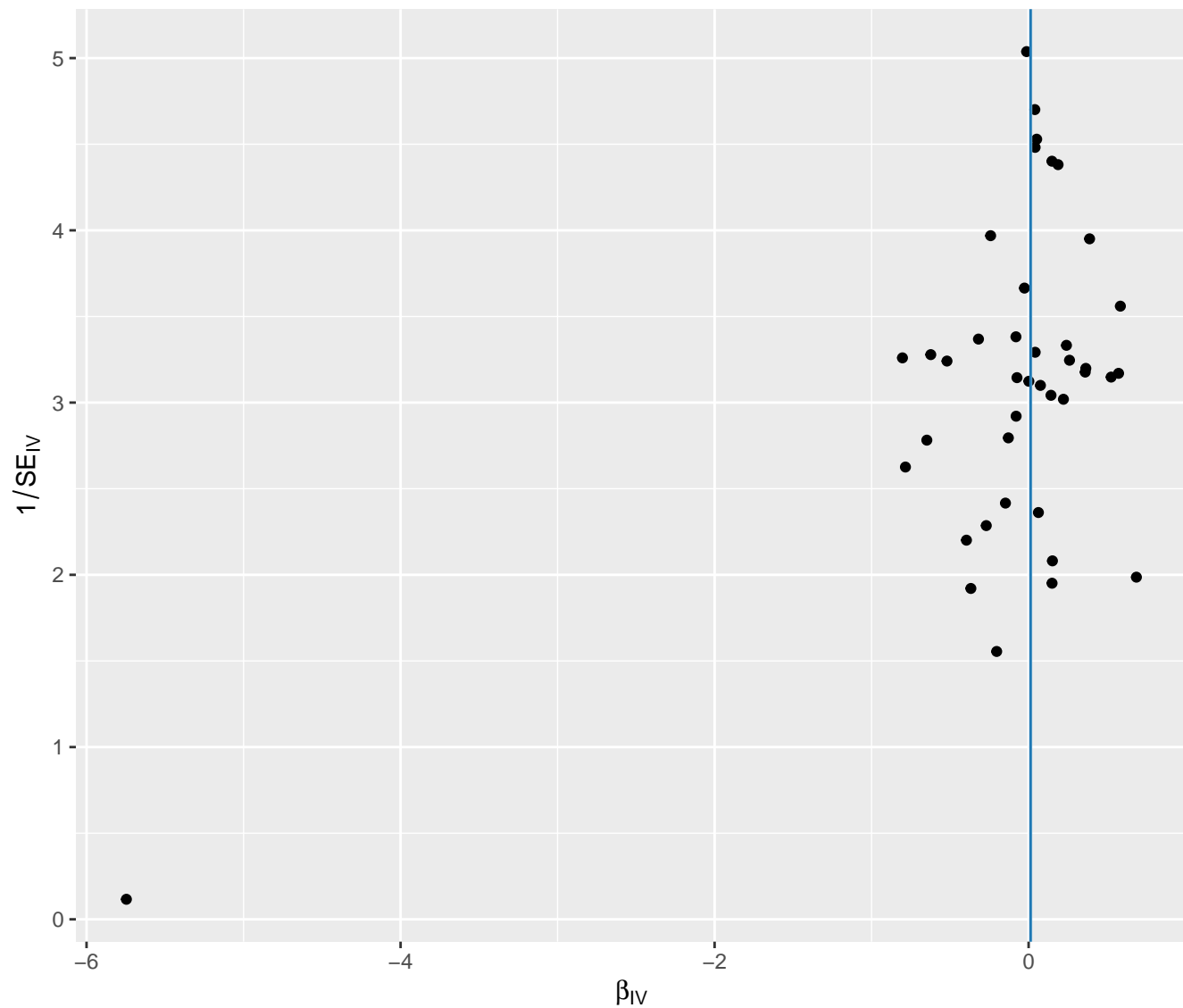

Supplement: Supplementary file 1 [file DataSheet_1.zip › supplementary material/CIM-OSTEOPOROSIS /GCST90274762.csv_funnel.pdf]

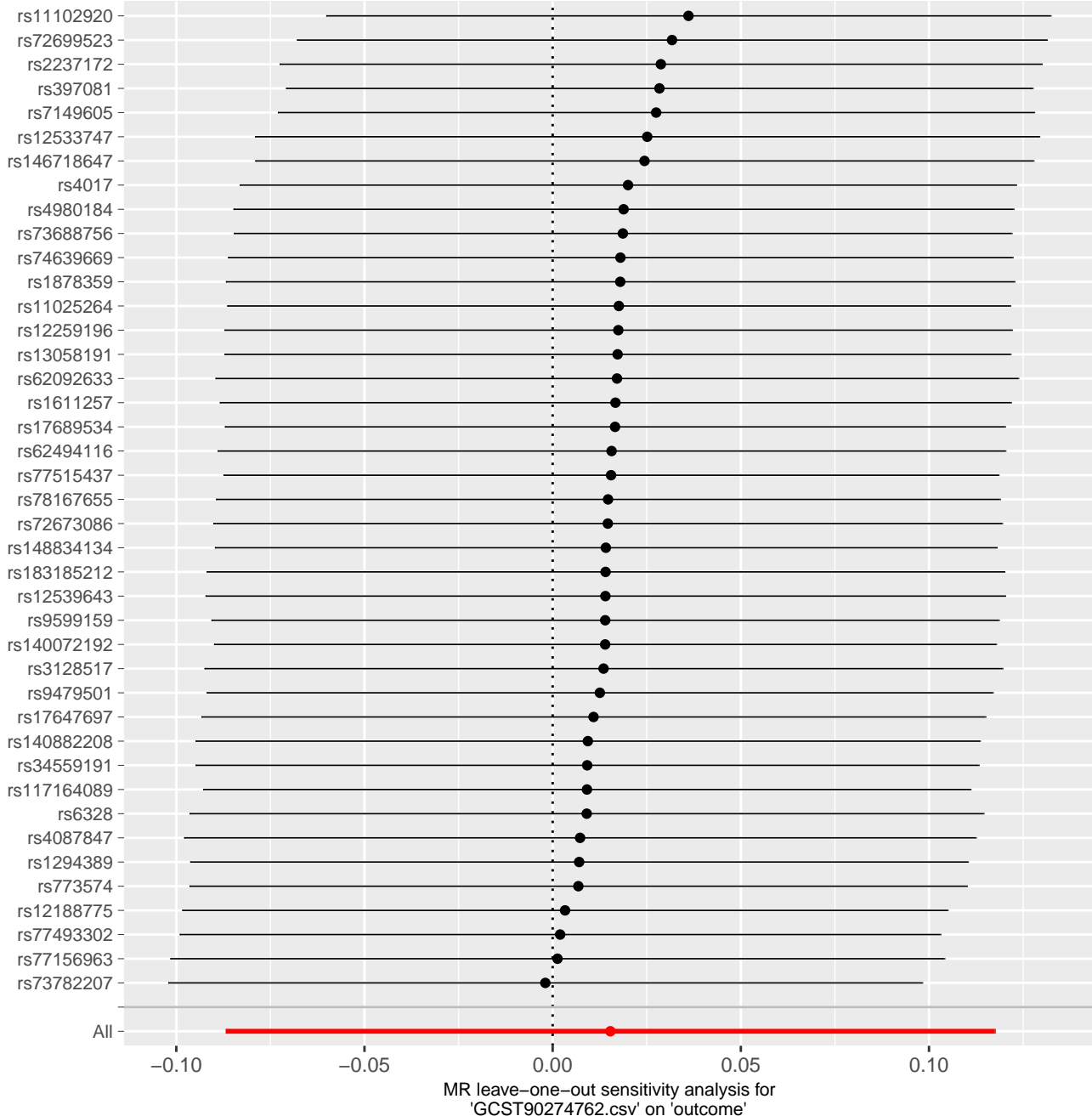

Supplement: Supplementary file 1 [file DataSheet_1.zip › supplementary material/CIM-OSTEOPOROSIS /GCST90274762.csv_leave_one_out.pdf]

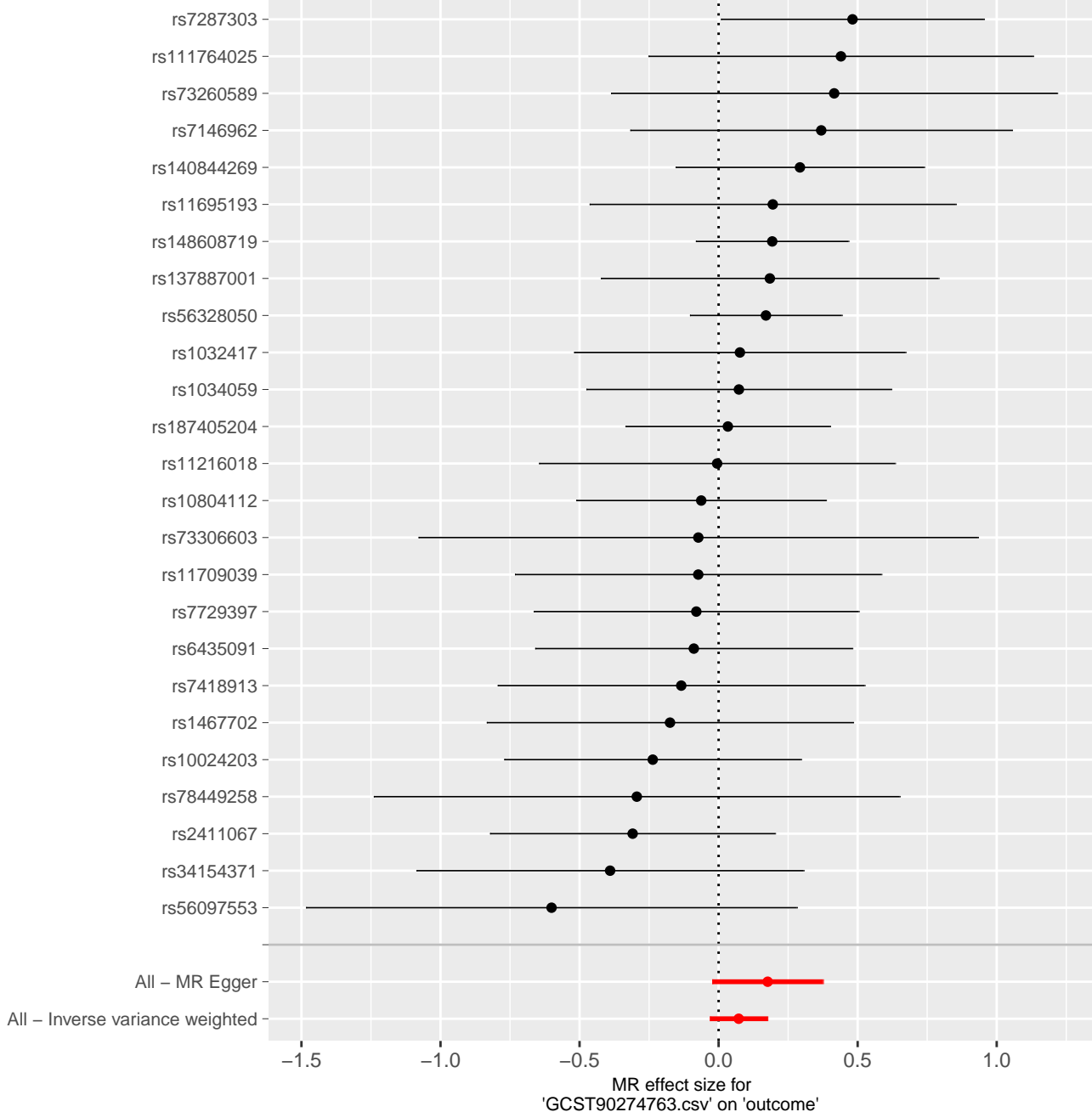

Supplement: Supplementary file 1 [file DataSheet_1.zip › supplementary material/CIM-OSTEOPOROSIS /GCST90274763.csv_forest.pdf]

# MR Method

- Inverse variance weighted
- MR Egger

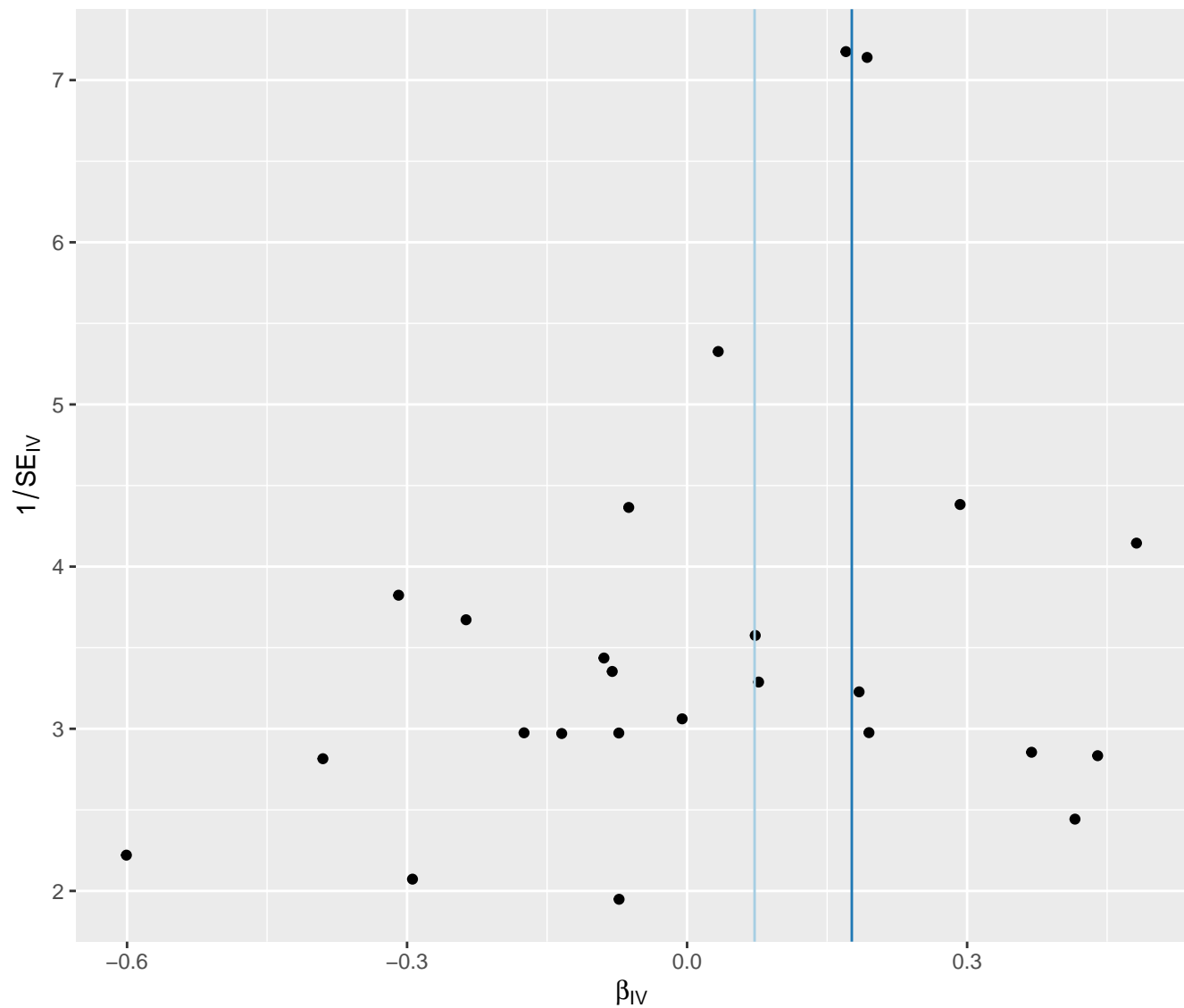

Supplement: Supplementary file 1 [file DataSheet_1.zip › supplementary material/CIM-OSTEOPOROSIS /GCST90274763.csv_funnel.pdf]

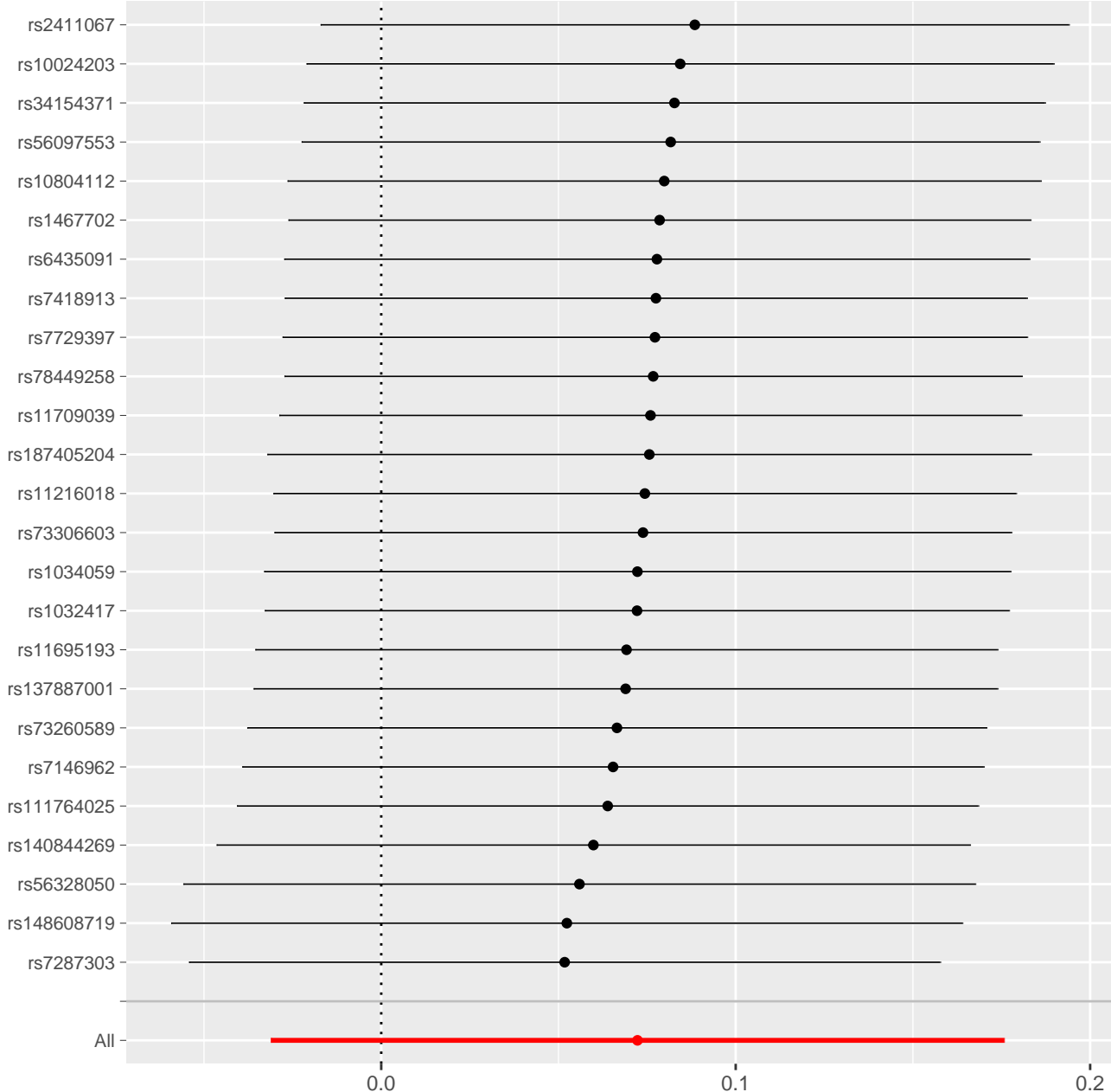

Supplement: Supplementary file 1 [file DataSheet_1.zip › supplementary material/CIM-OSTEOPOROSIS /GCST90274763.csv_leave_one_out.pdf]

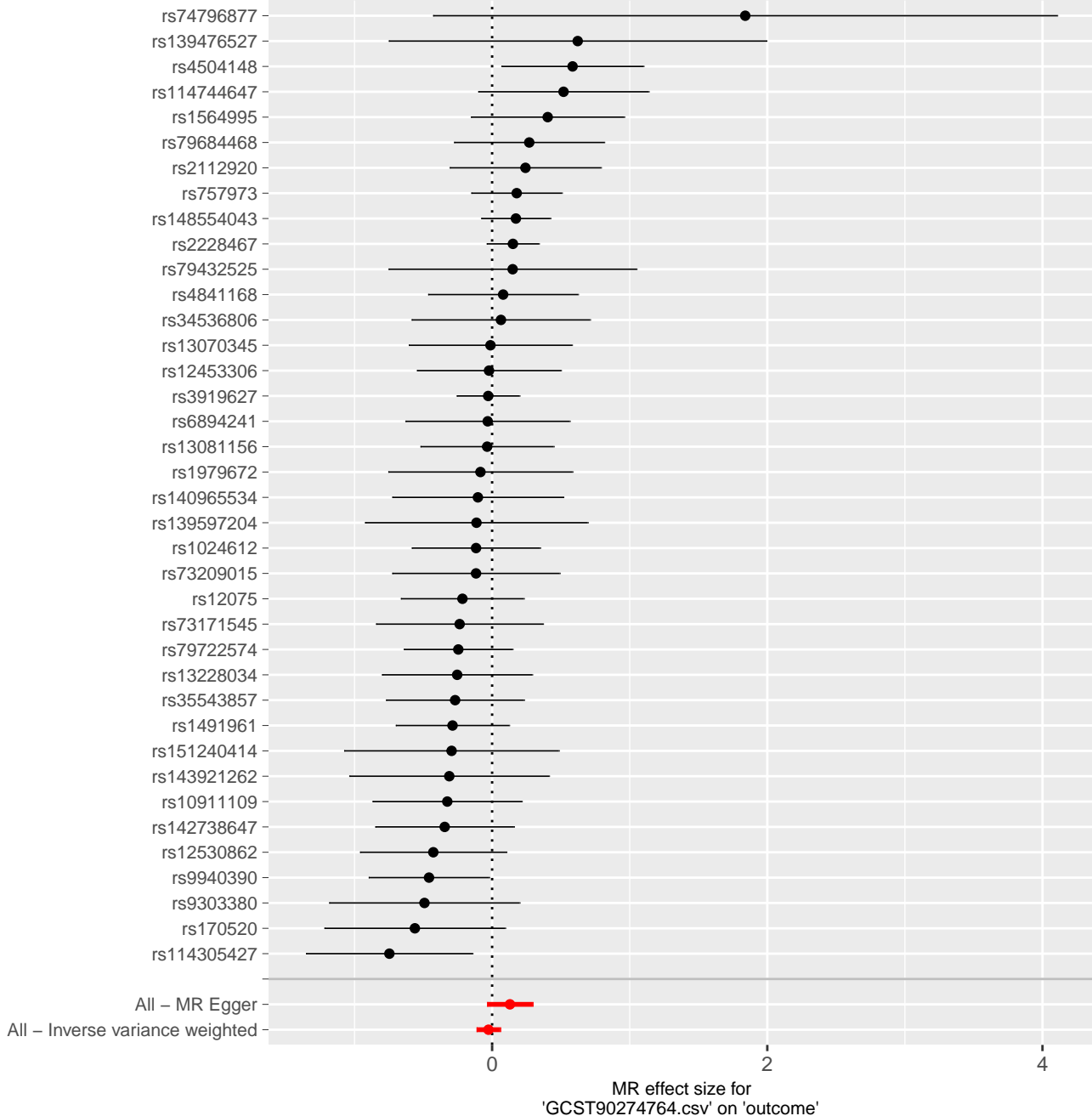

Supplement: Supplementary file 1 [file DataSheet_1.zip › supplementary material/CIM-OSTEOPOROSIS /GCST90274764.csv_forest.pdf]

# MR Method

- Inverse variance weighted
- MR Egger

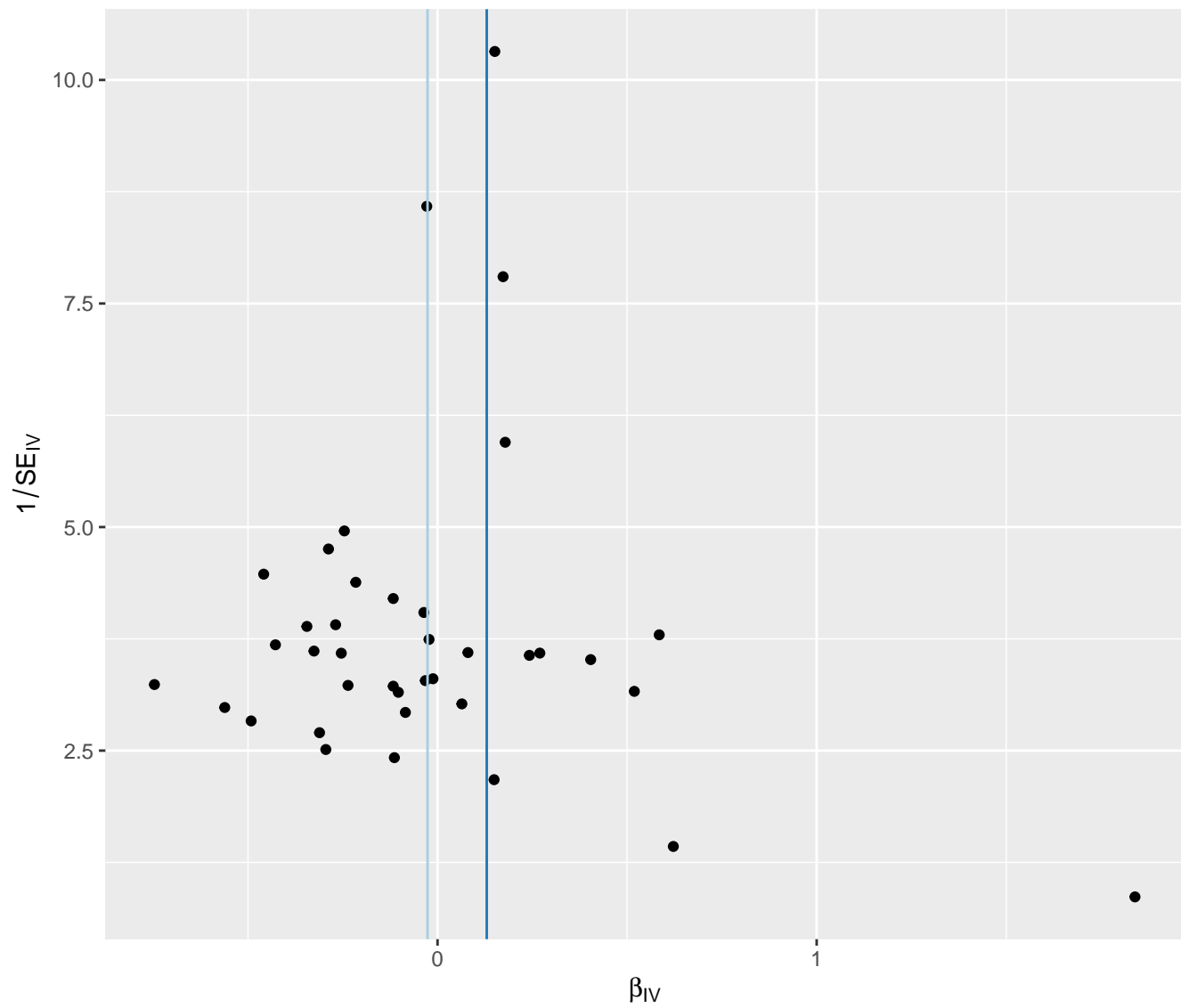

Supplement: Supplementary file 1 [file DataSheet_1.zip › supplementary material/CIM-OSTEOPOROSIS /GCST90274764.csv_funnel.pdf]

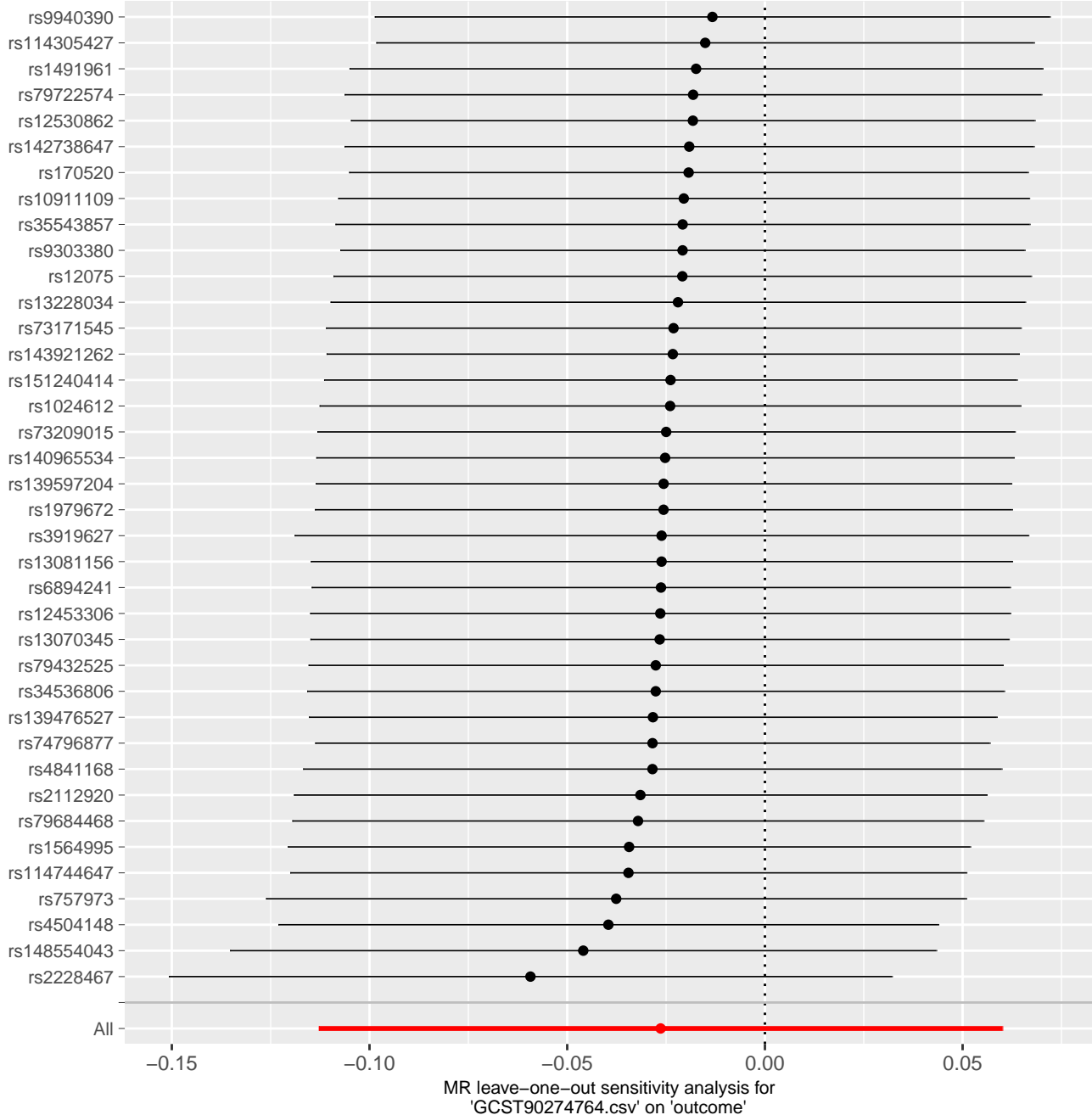

Supplement: Supplementary file 1 [file DataSheet_1.zip › supplementary material/CIM-OSTEOPOROSIS /GCST90274764.csv_leave_one_out.pdf]

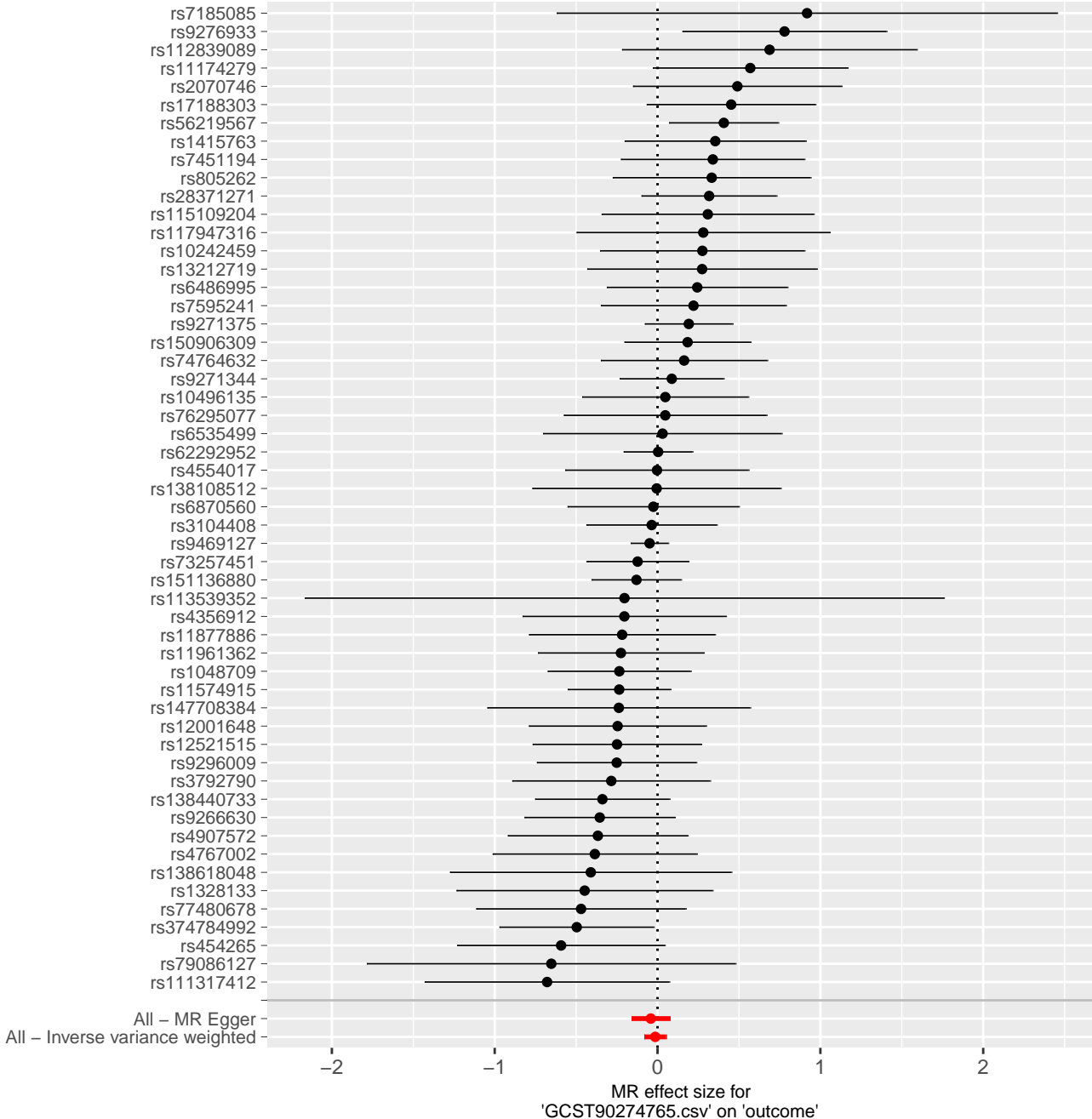

Supplement: Supplementary file 1 [file DataSheet_1.zip › supplementary material/CIM-OSTEOPOROSIS /GCST90274765.csv_forest.pdf]

# MR Method

- Inverse variance weighted
- MR Egger

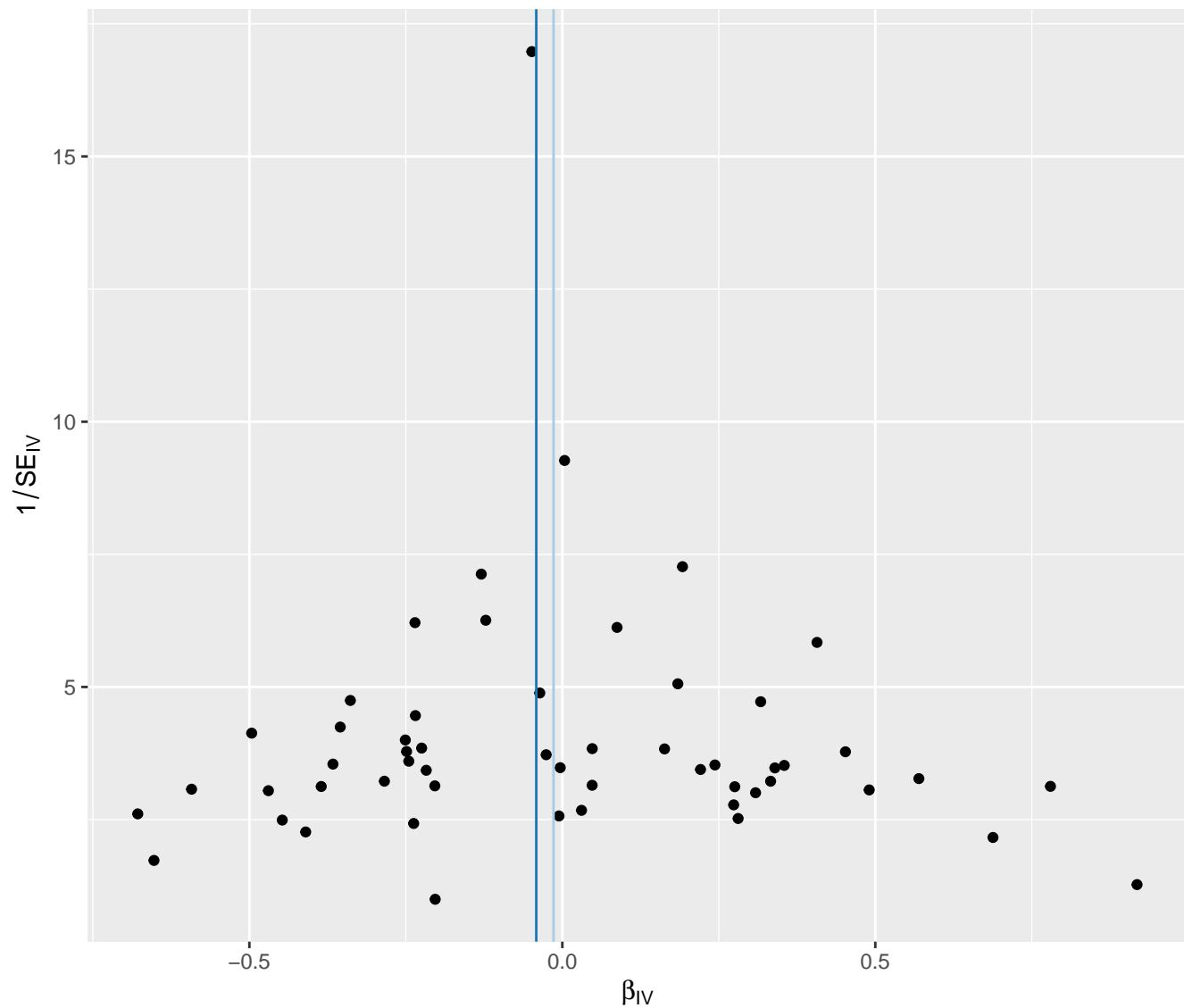

Supplement: Supplementary file 1 [file DataSheet_1.zip › supplementary material/CIM-OSTEOPOROSIS /GCST90274765.csv_funnel.pdf]

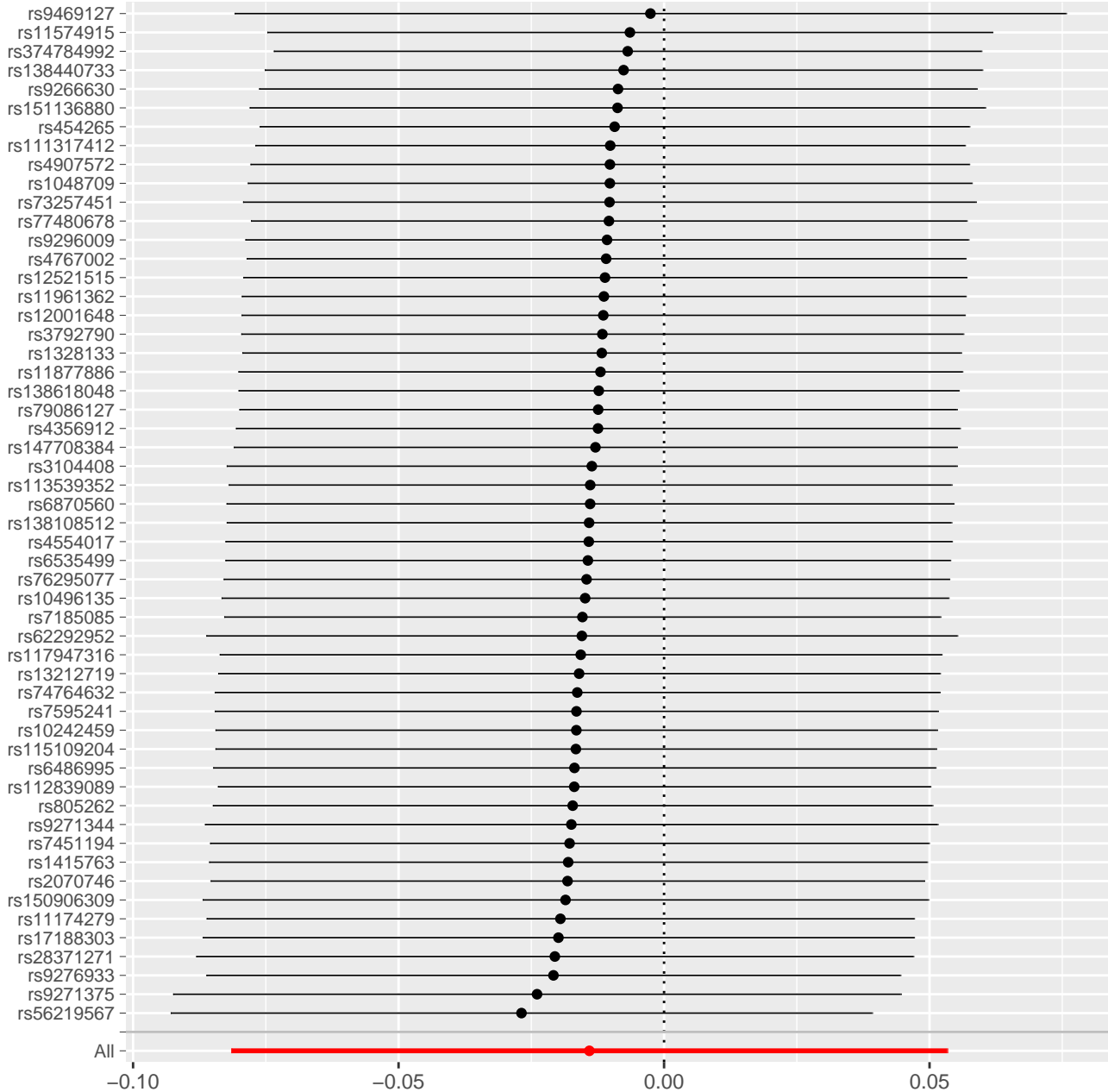

MR leave-one-out sensitivity analysis for  
'GCST90274765.csv' on 'outcome'

Supplement: Supplementary file 1 [file DataSheet_1.zip › supplementary material/CIM-OSTEOPOROSIS /GCST90274765.csv_leave_one_out.pdf]

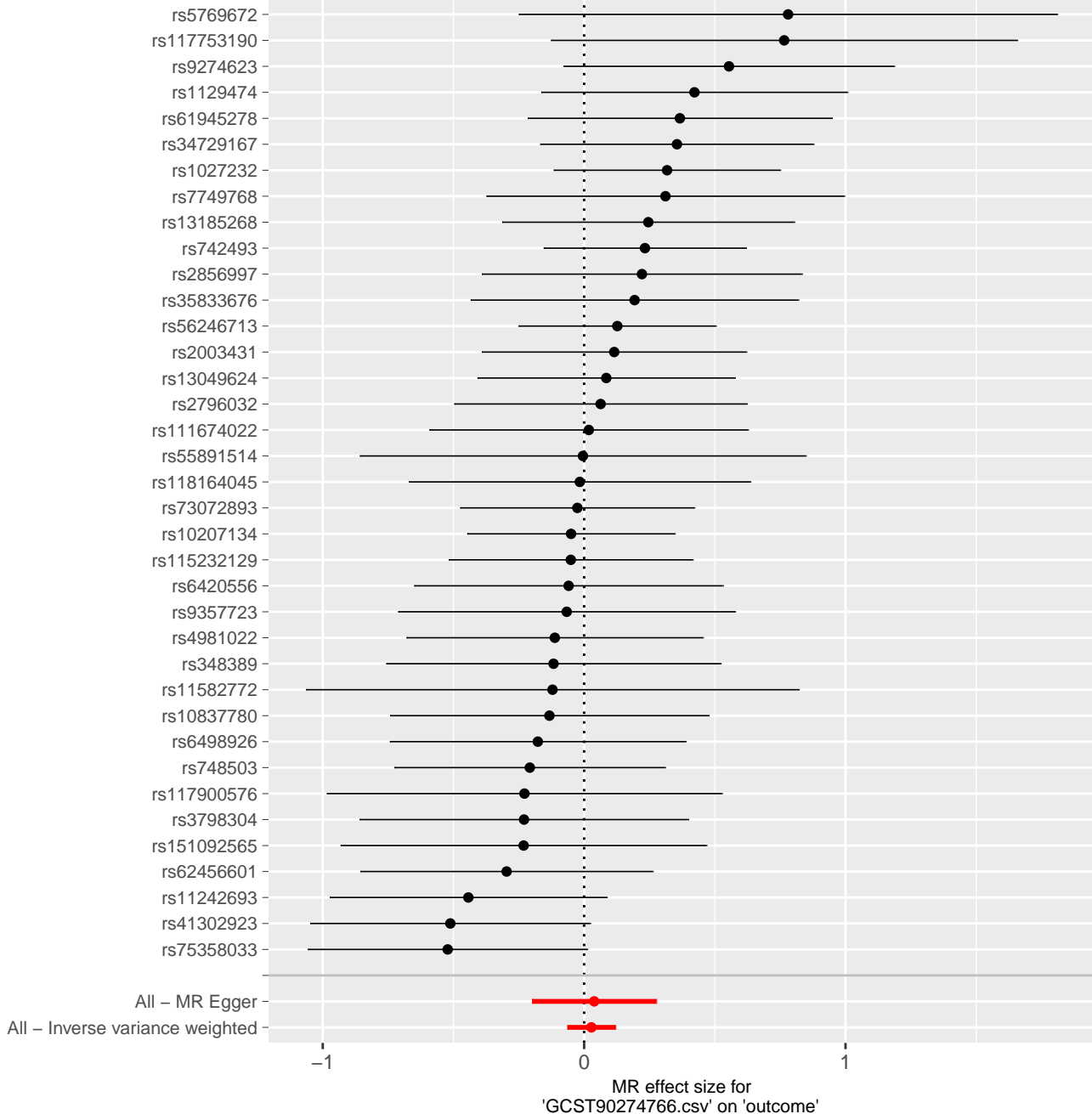

Supplement: Supplementary file 1 [file DataSheet_1.zip › supplementary material/CIM-OSTEOPOROSIS /GCST90274766.csv_forest.pdf]

# MR Method

- Inverse variance weighted
- MR Egger

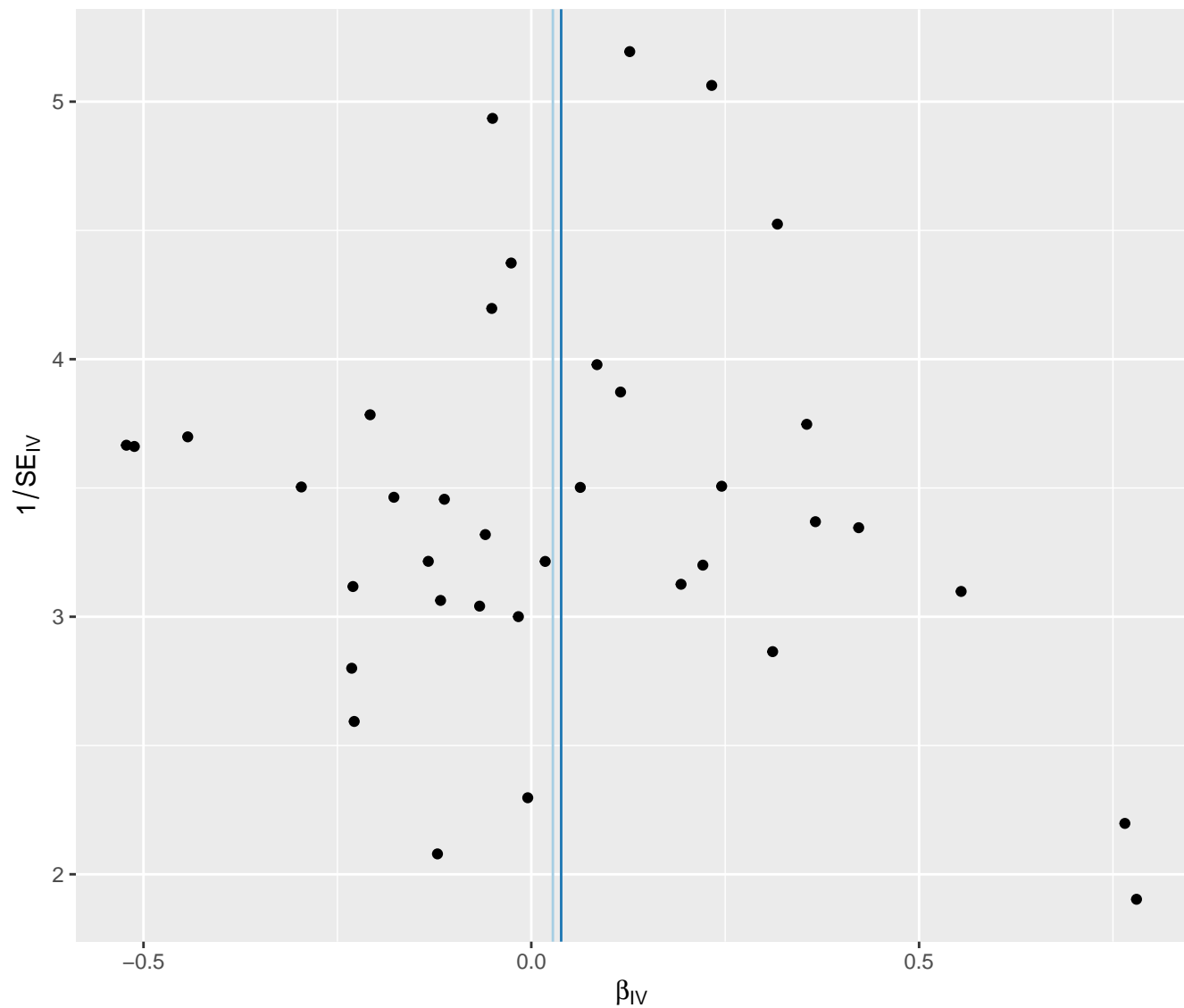

Supplement: Supplementary file 1 [file DataSheet_1.zip › supplementary material/CIM-OSTEOPOROSIS /GCST90274766.csv_funnel.pdf]

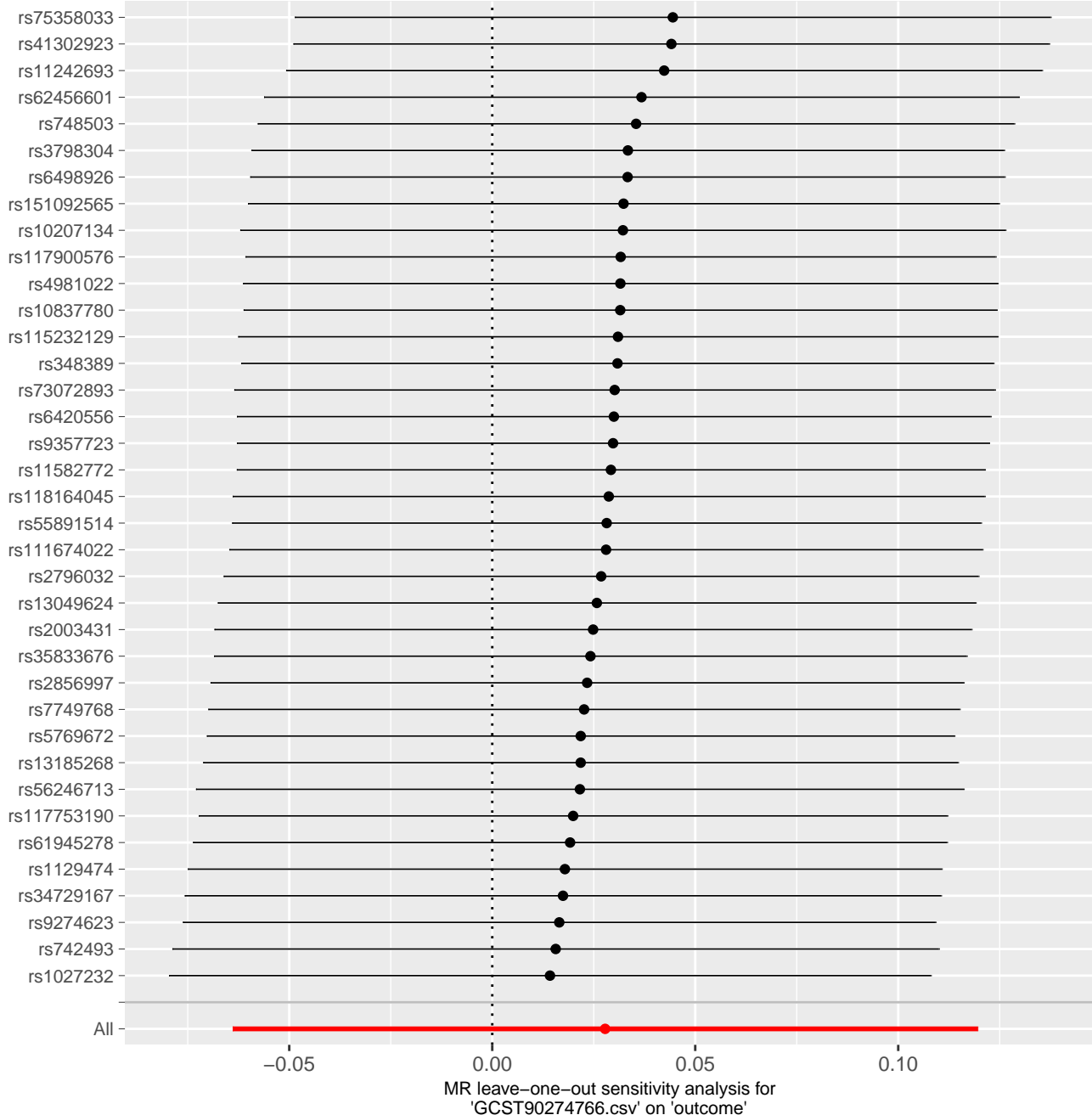

Supplement: Supplementary file 1 [file DataSheet_1.zip › supplementary material/CIM-OSTEOPOROSIS /GCST90274766.csv_leave_one_out.pdf]

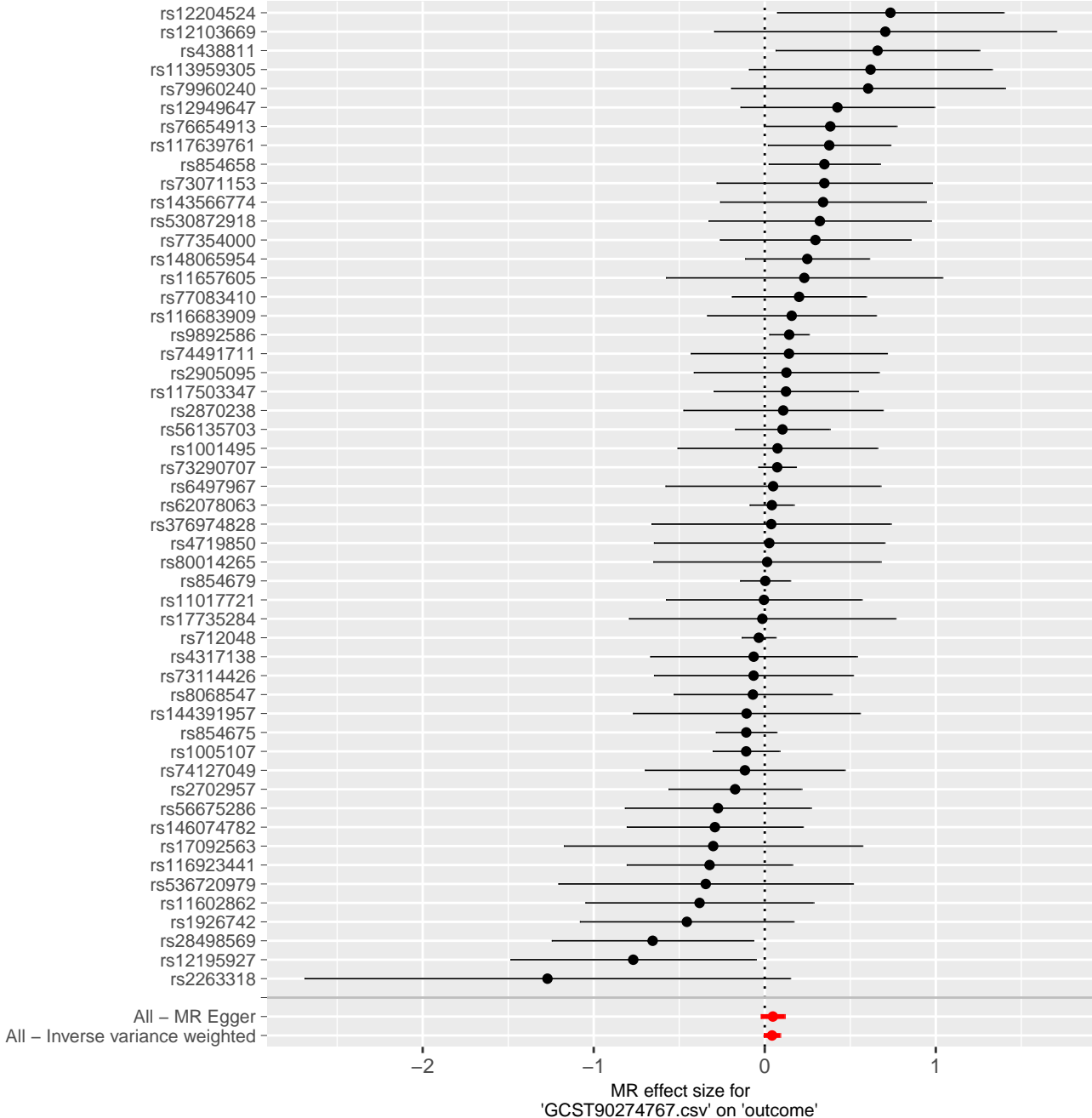

Supplement: Supplementary file 1 [file DataSheet_1.zip › supplementary material/CIM-OSTEOPOROSIS /GCST90274767.csv_forest.pdf]

# MR Method

- Inverse variance weighted
- MR Egger

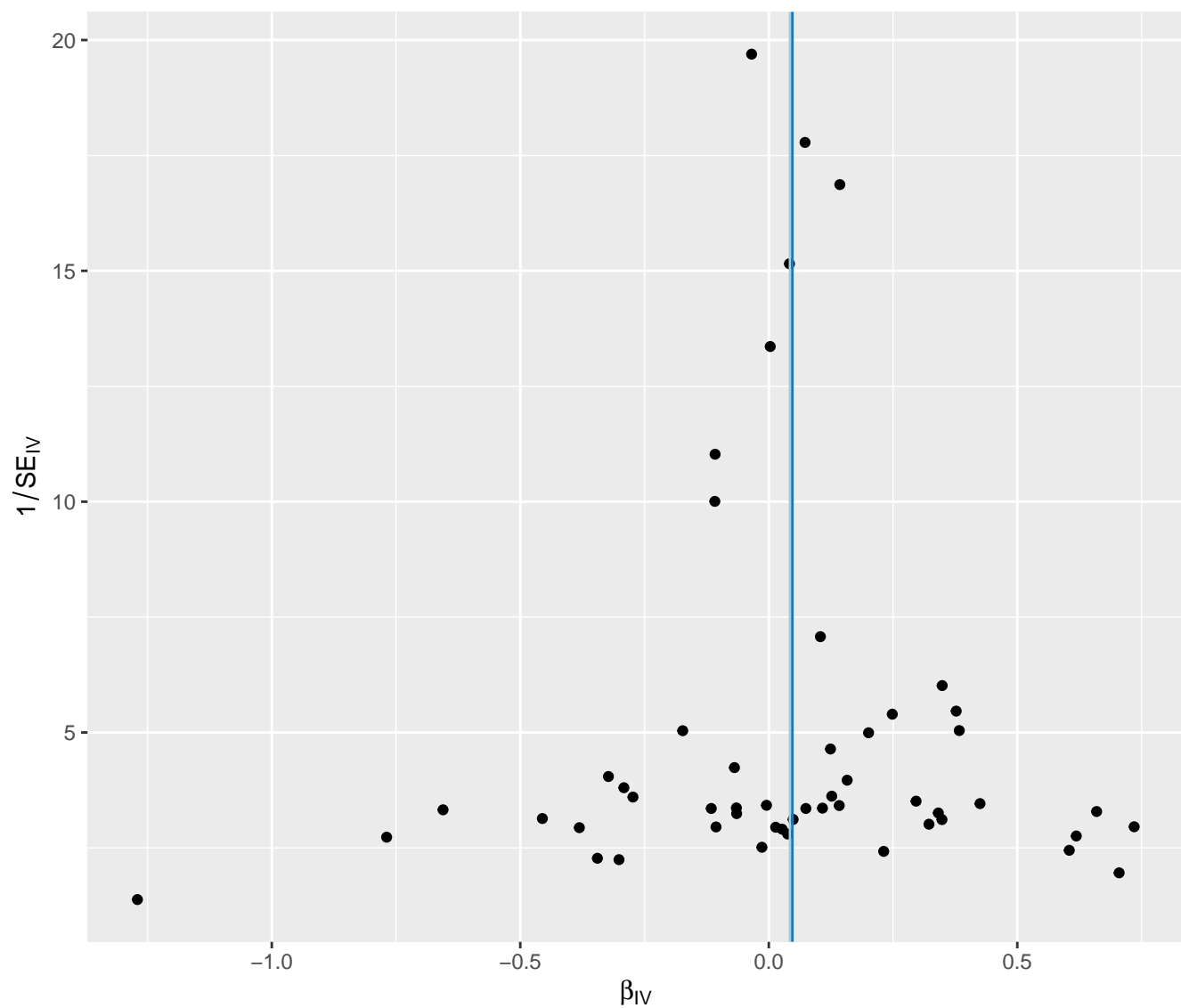

Supplement: Supplementary file 1 [file DataSheet_1.zip › supplementary material/CIM-OSTEOPOROSIS /GCST90274767.csv_funnel.pdf]

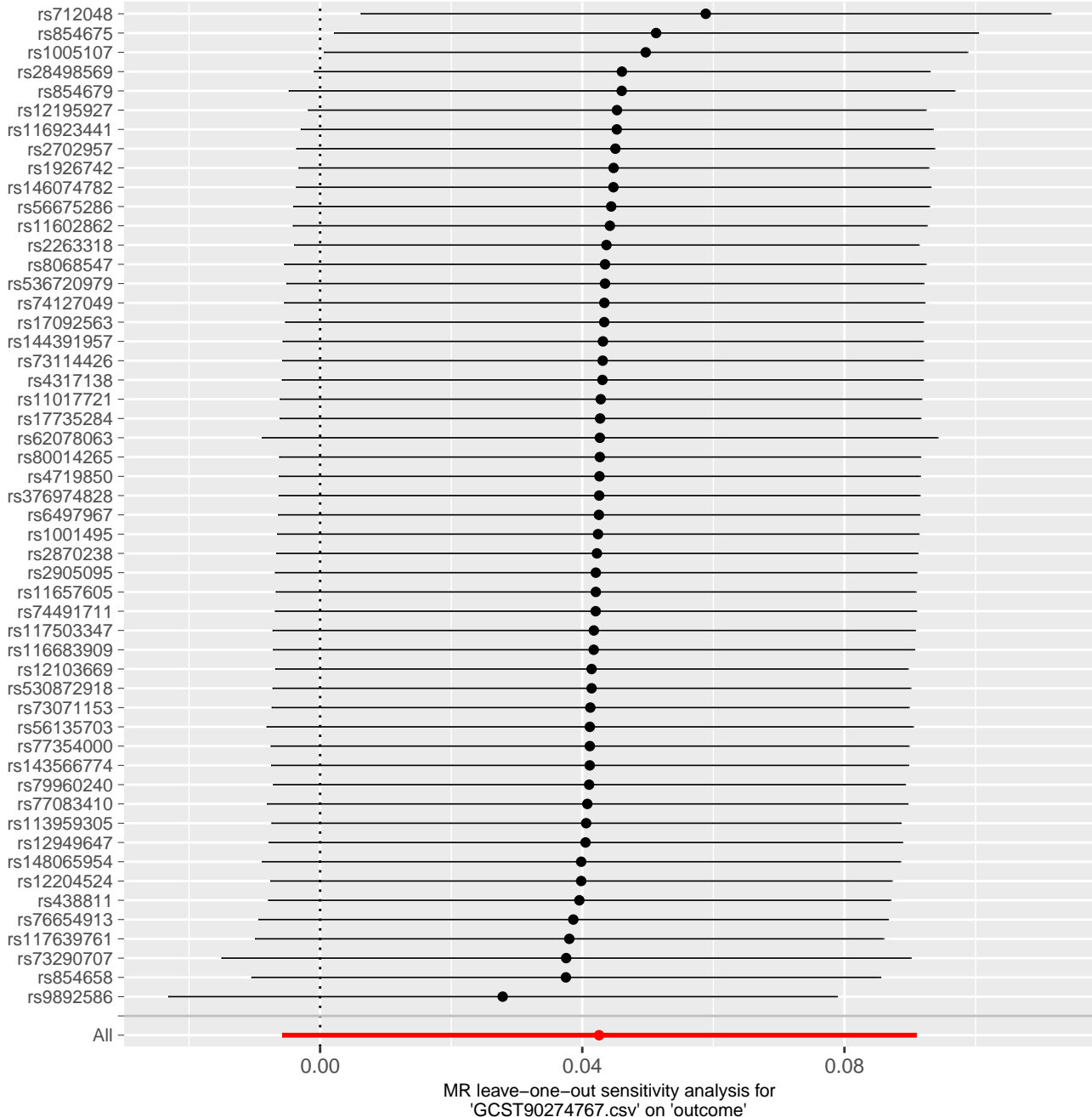

Supplement: Supplementary file 1 [file DataSheet_1.zip › supplementary material/CIM-OSTEOPOROSIS /GCST90274767.csv_leave_one_out.pdf]

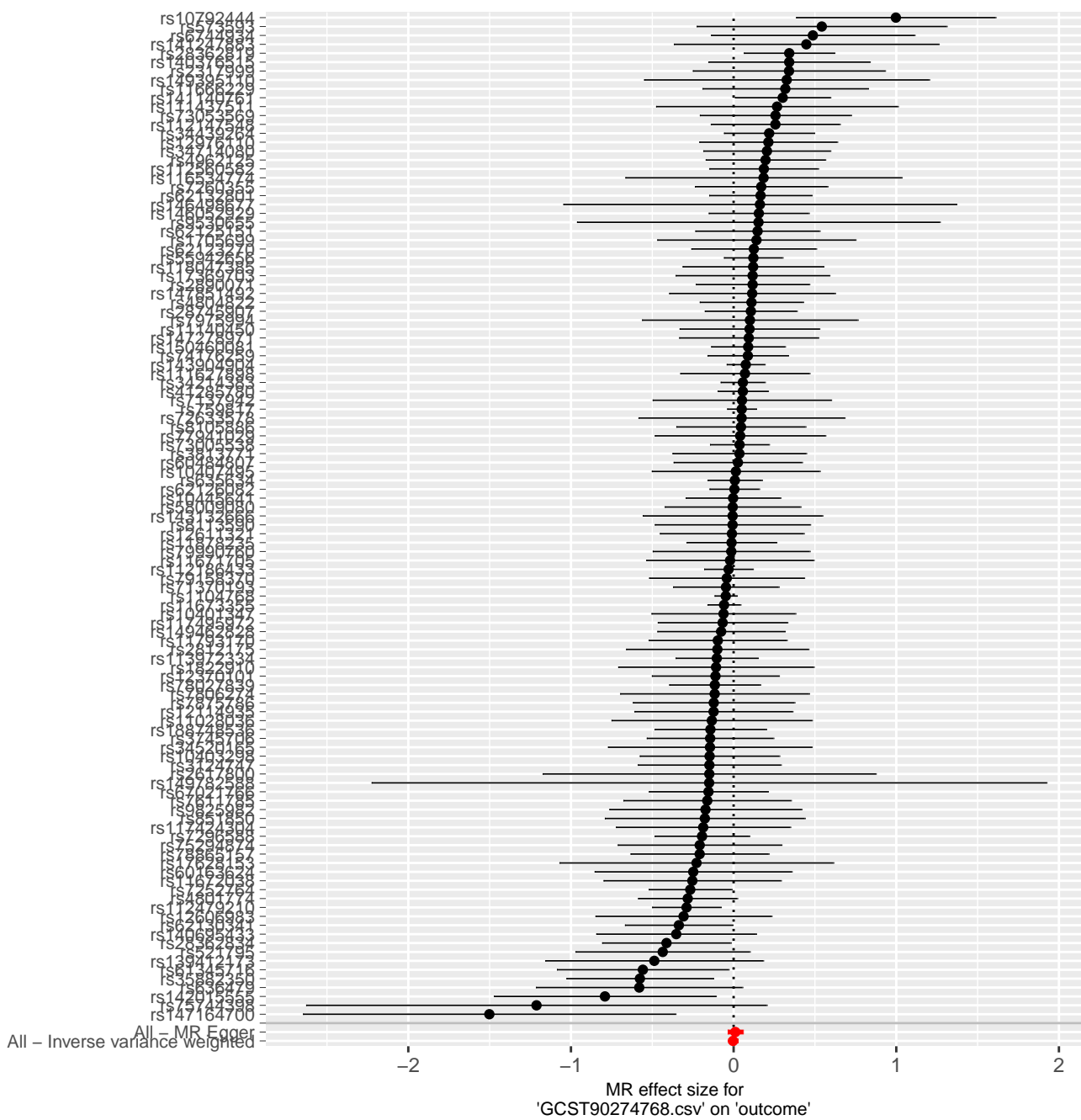

Supplement: Supplementary file 1 [file DataSheet_1.zip › supplementary material/CIM-OSTEOPOROSIS /GCST90274768.csv_forest.pdf]

# MR Method

- Inverse variance weighted
- MR Egger

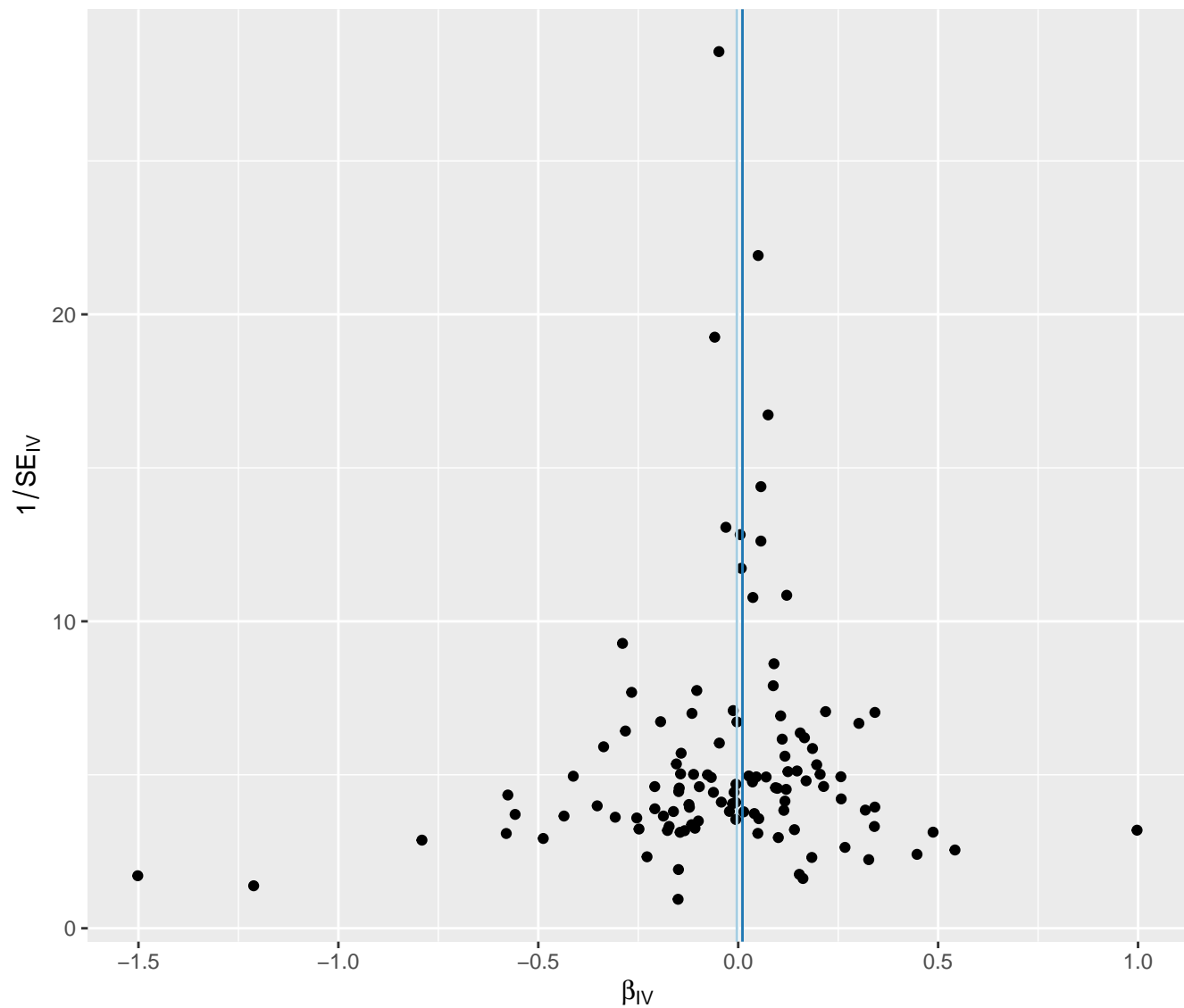

Supplement: Supplementary file 1 [file DataSheet_1.zip › supplementary material/CIM-OSTEOPOROSIS /GCST90274768.csv_funnel.pdf]

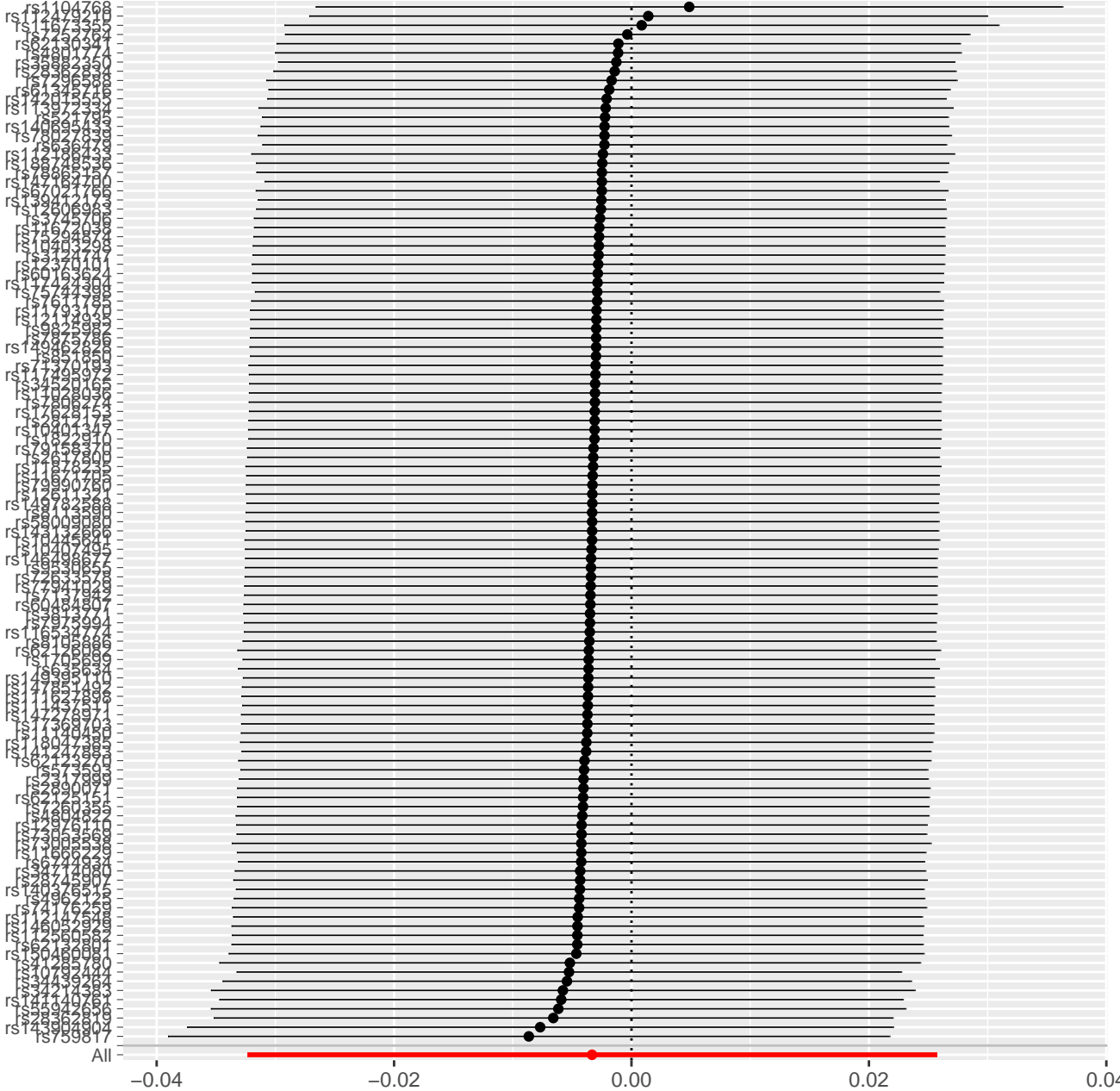

Supplement: Supplementary file 1 [file DataSheet_1.zip › supplementary material/CIM-OSTEOPOROSIS /GCST90274768.csv_leave_one_out.pdf]

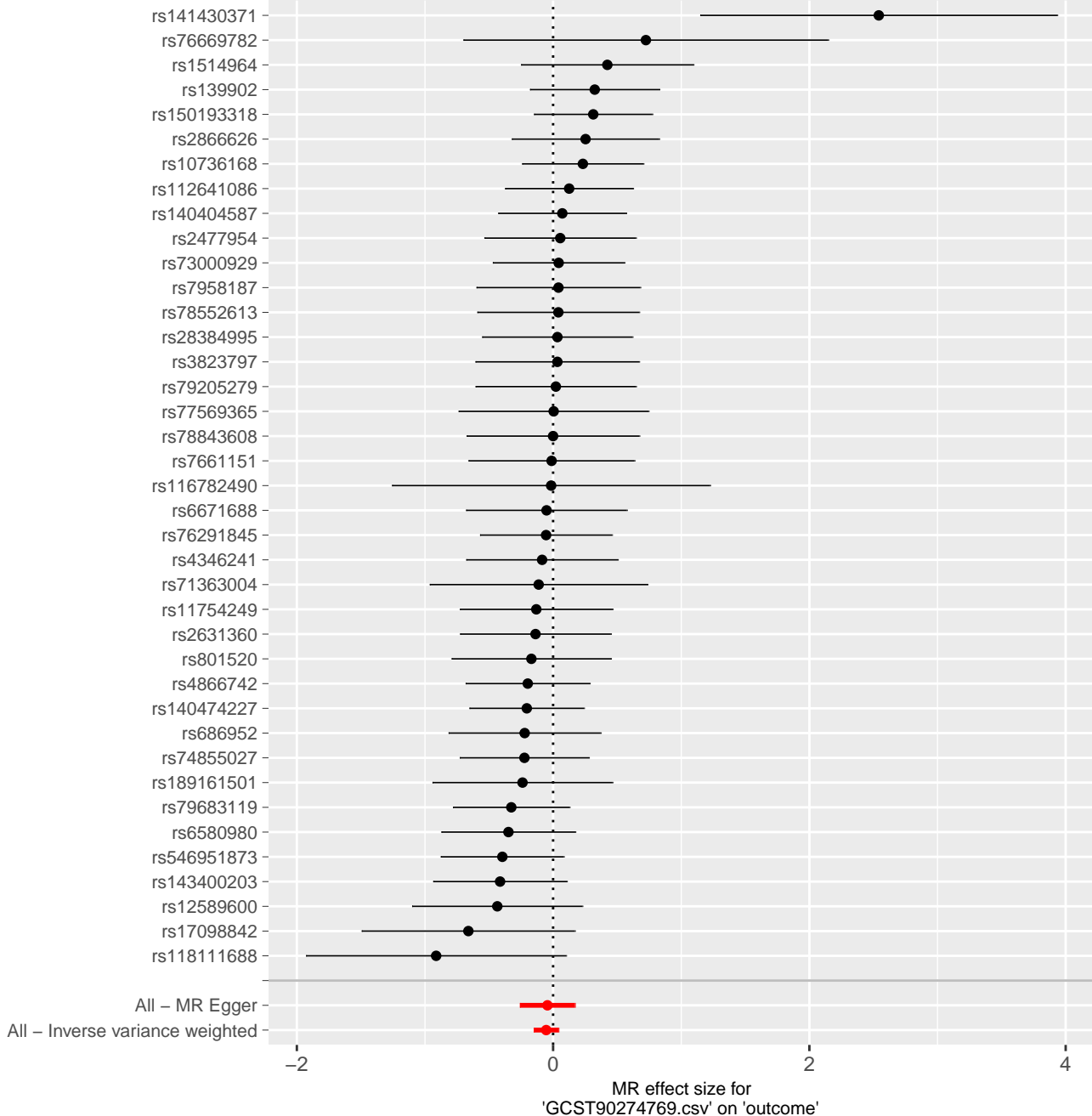

Supplement: Supplementary file 1 [file DataSheet_1.zip › supplementary material/CIM-OSTEOPOROSIS /GCST90274769.csv_forest.pdf]

# MR Method

- Inverse variance weighted
- MR Egger

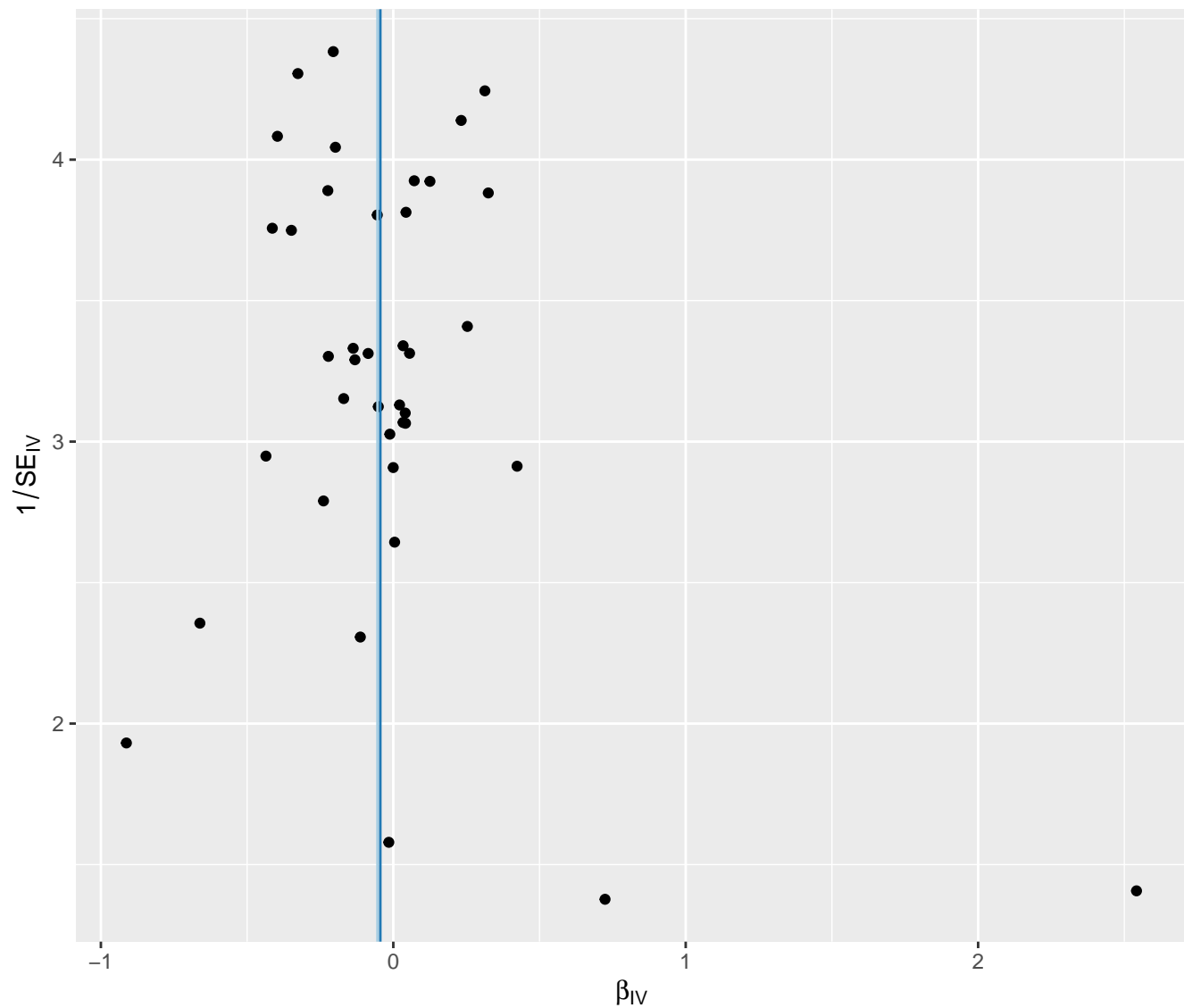

Supplement: Supplementary file 1 [file DataSheet_1.zip › supplementary material/CIM-OSTEOPOROSIS /GCST90274769.csv_funnel.pdf]

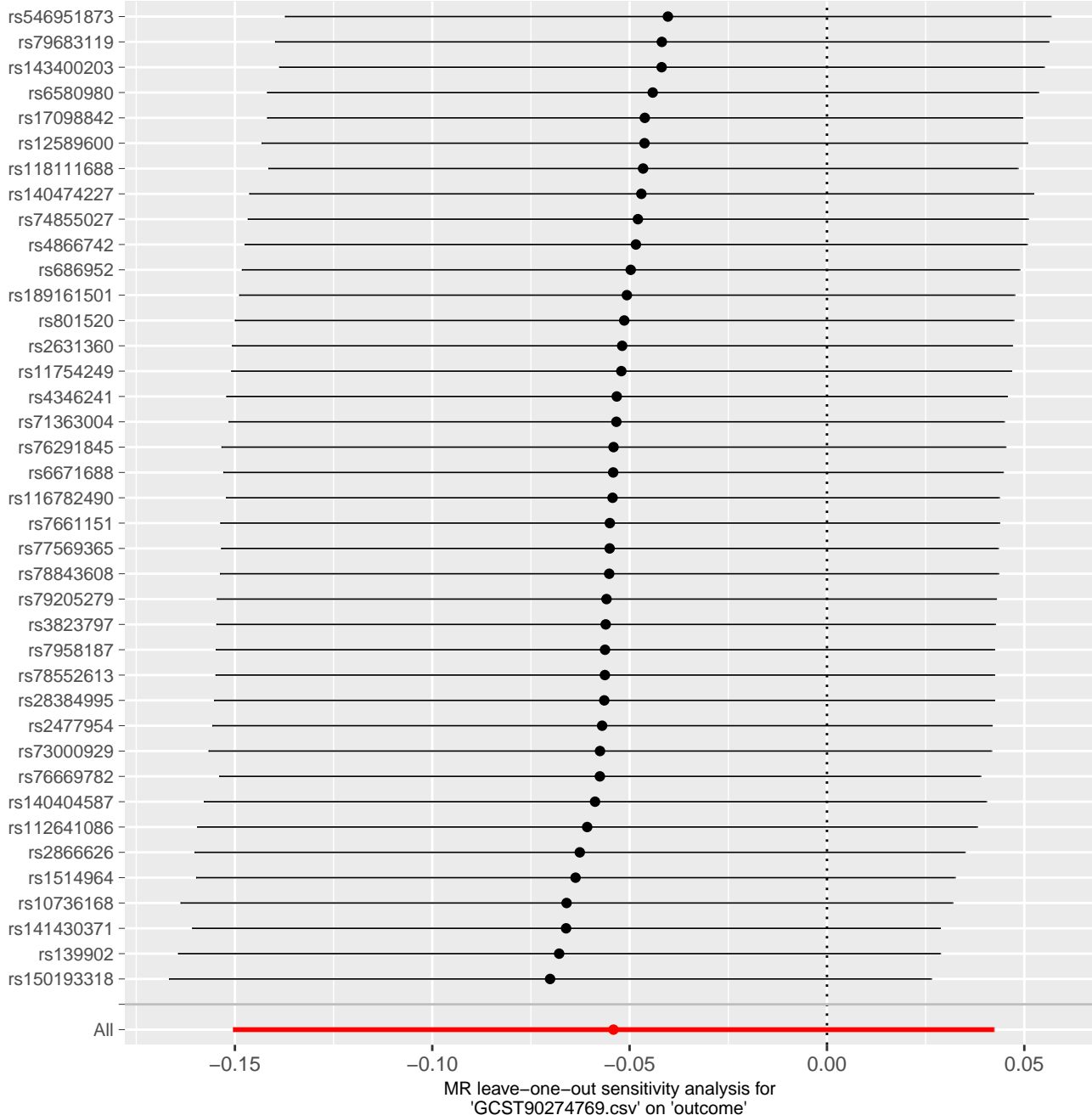

Supplement: Supplementary file 1 [file DataSheet_1.zip › supplementary material/CIM-OSTEOPOROSIS /GCST90274769.csv_leave_one_out.pdf]

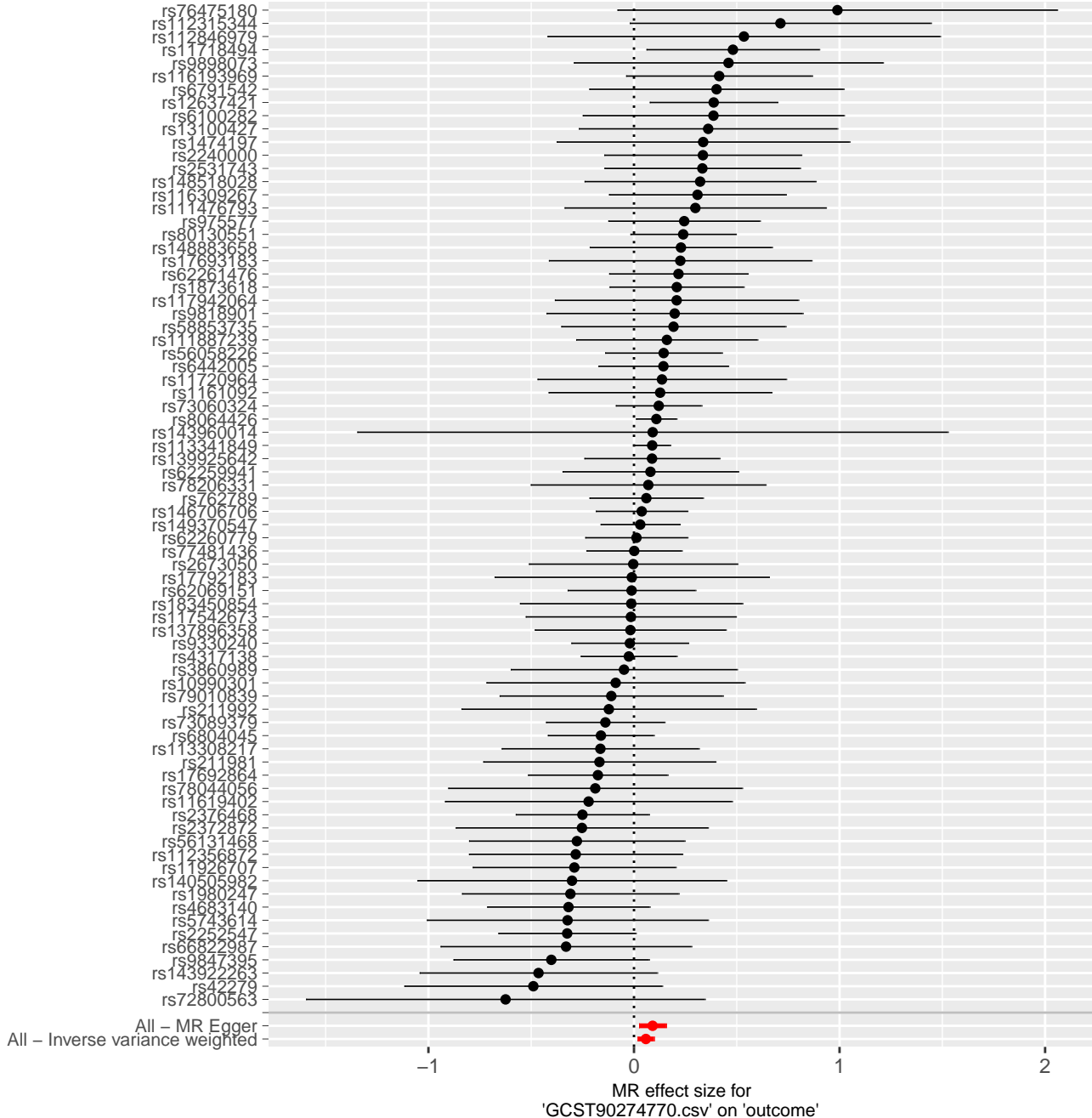

Supplement: Supplementary file 1 [file DataSheet_1.zip › supplementary material/CIM-OSTEOPOROSIS /GCST90274770.csv_forest.pdf]

# MR Method

- Inverse variance weighted
- MR Egger

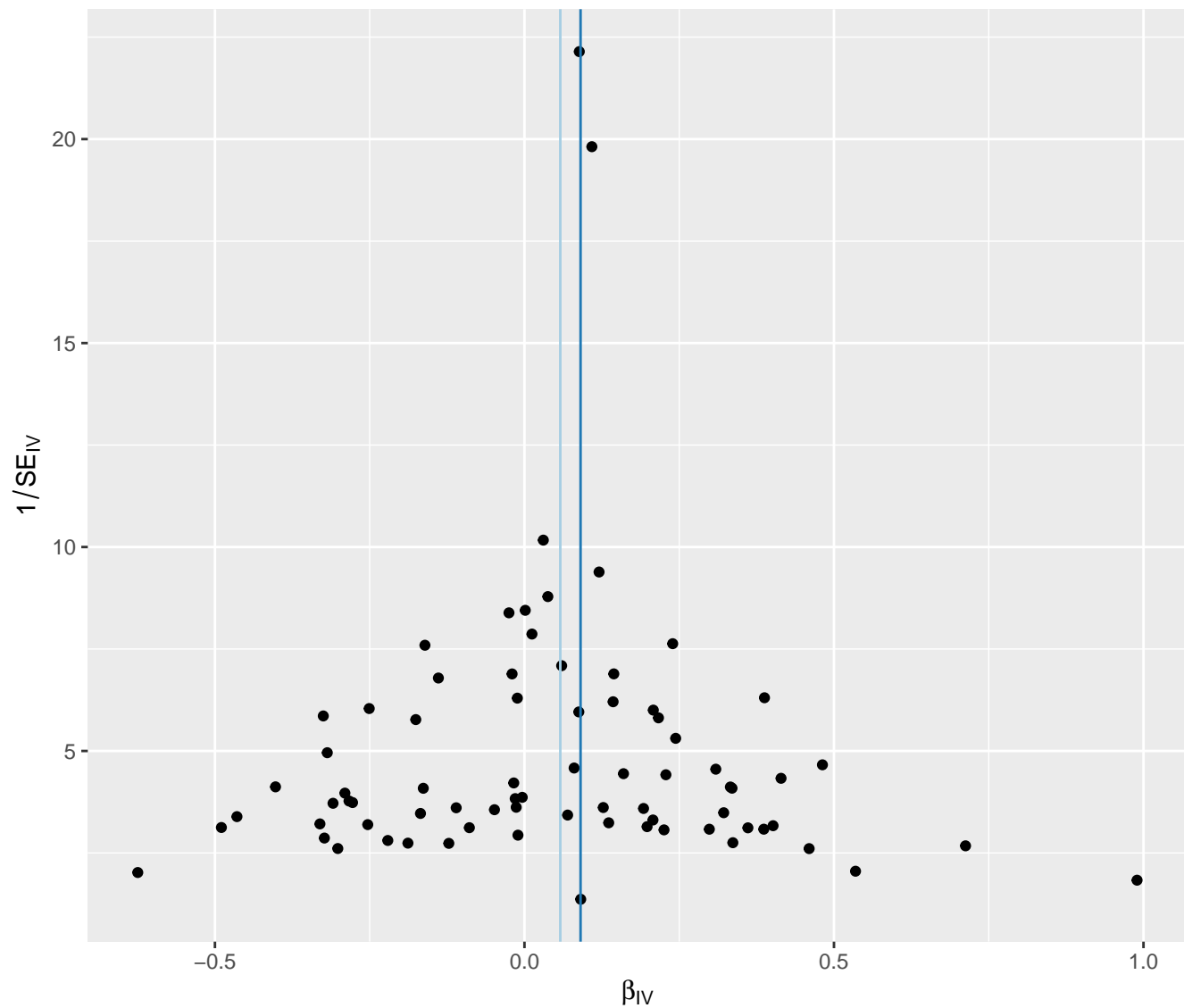

Supplement: Supplementary file 1 [file DataSheet_1.zip › supplementary material/CIM-OSTEOPOROSIS /GCST90274770.csv_funnel.pdf]

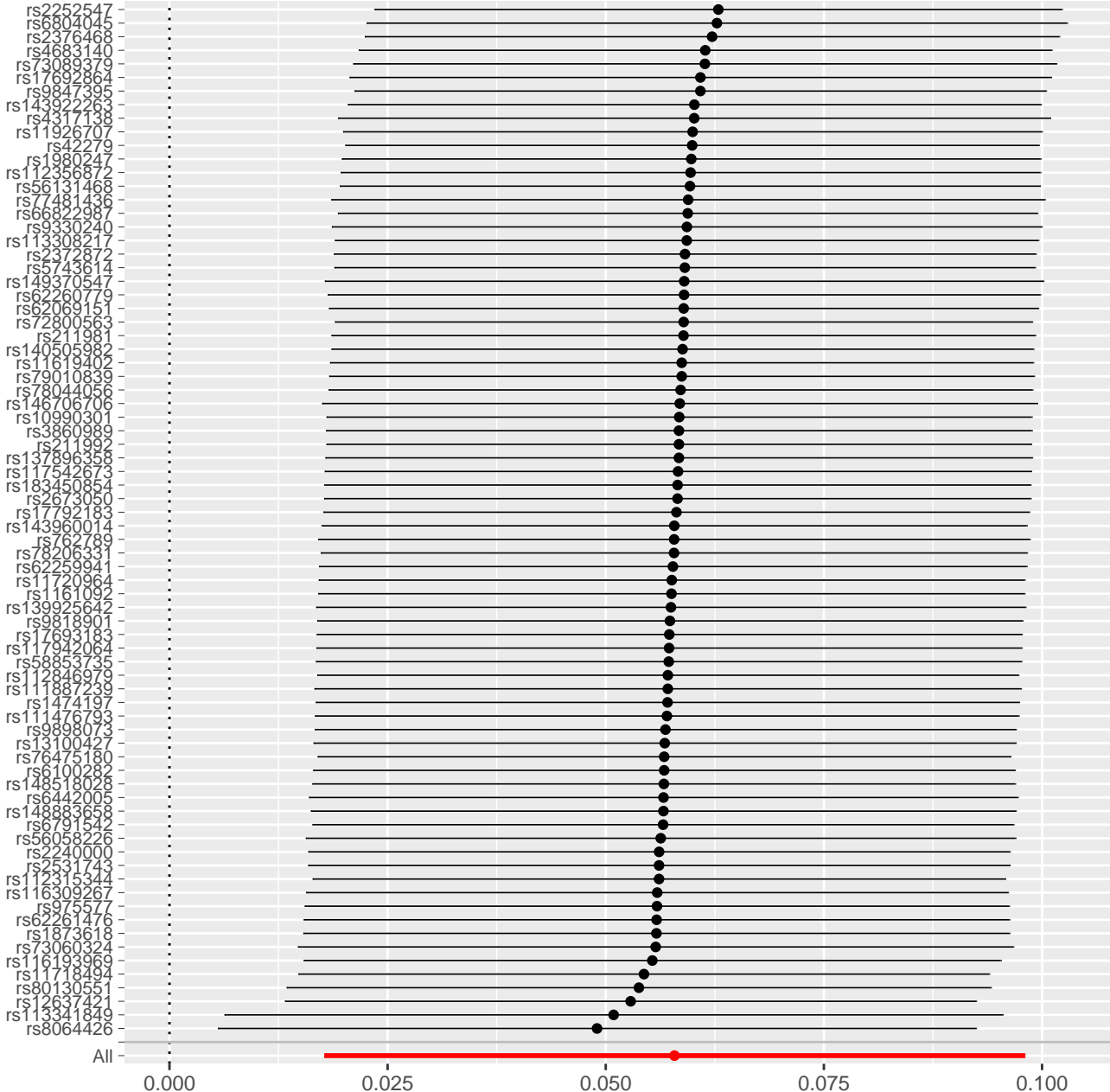

MR leave-one-out sensitivity analysis for  
'GCST90274770.csv' on 'outcome'

Supplement: Supplementary file 1 [file DataSheet_1.zip › supplementary material/CIM-OSTEOPOROSIS /GCST90274770.csv_leave_one_out.pdf]

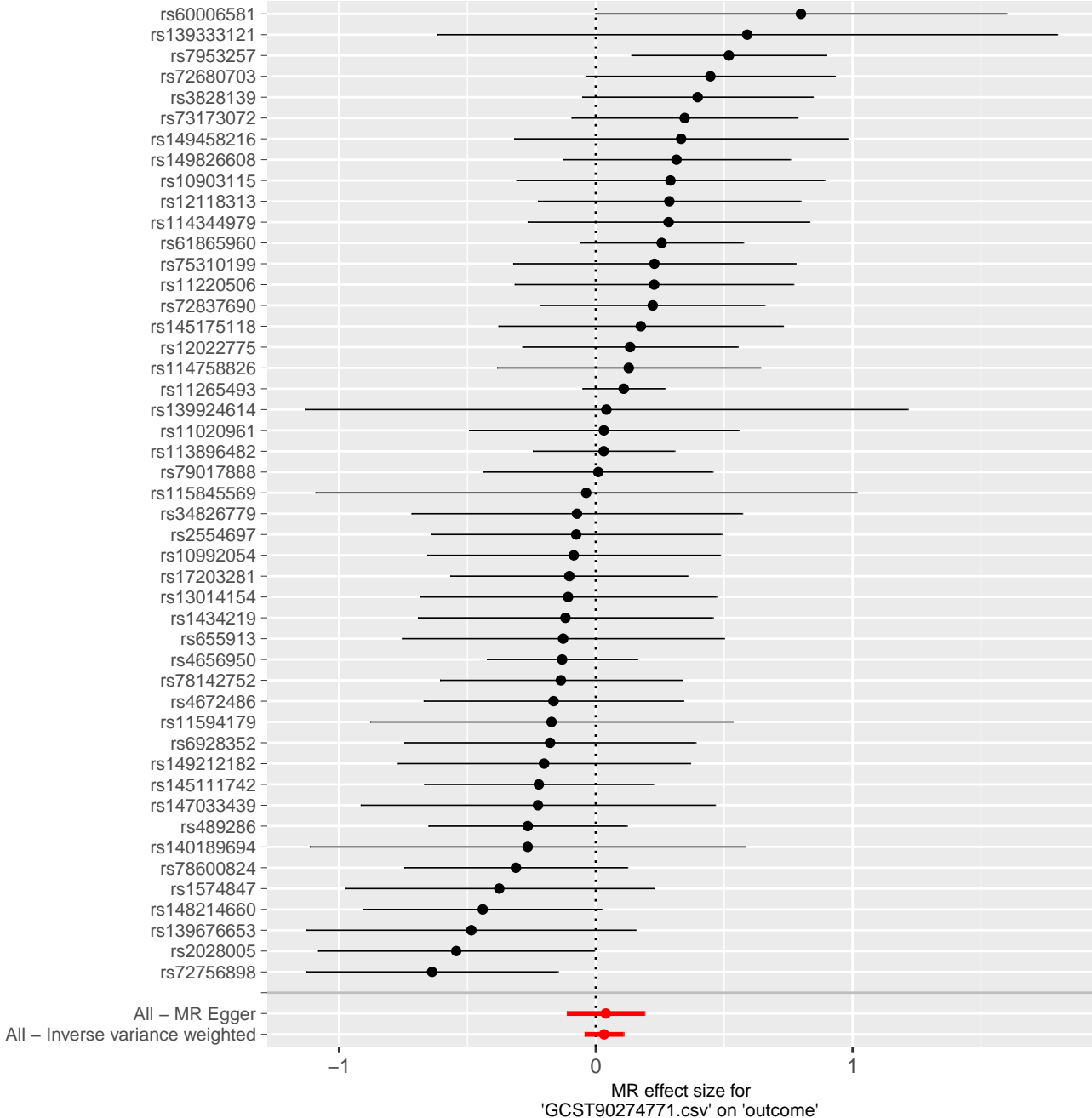

Supplement: Supplementary file 1 [file DataSheet_1.zip › supplementary material/CIM-OSTEOPOROSIS /GCST90274771.csv_forest.pdf]

# MR Method

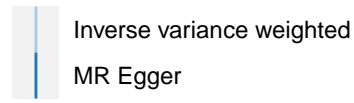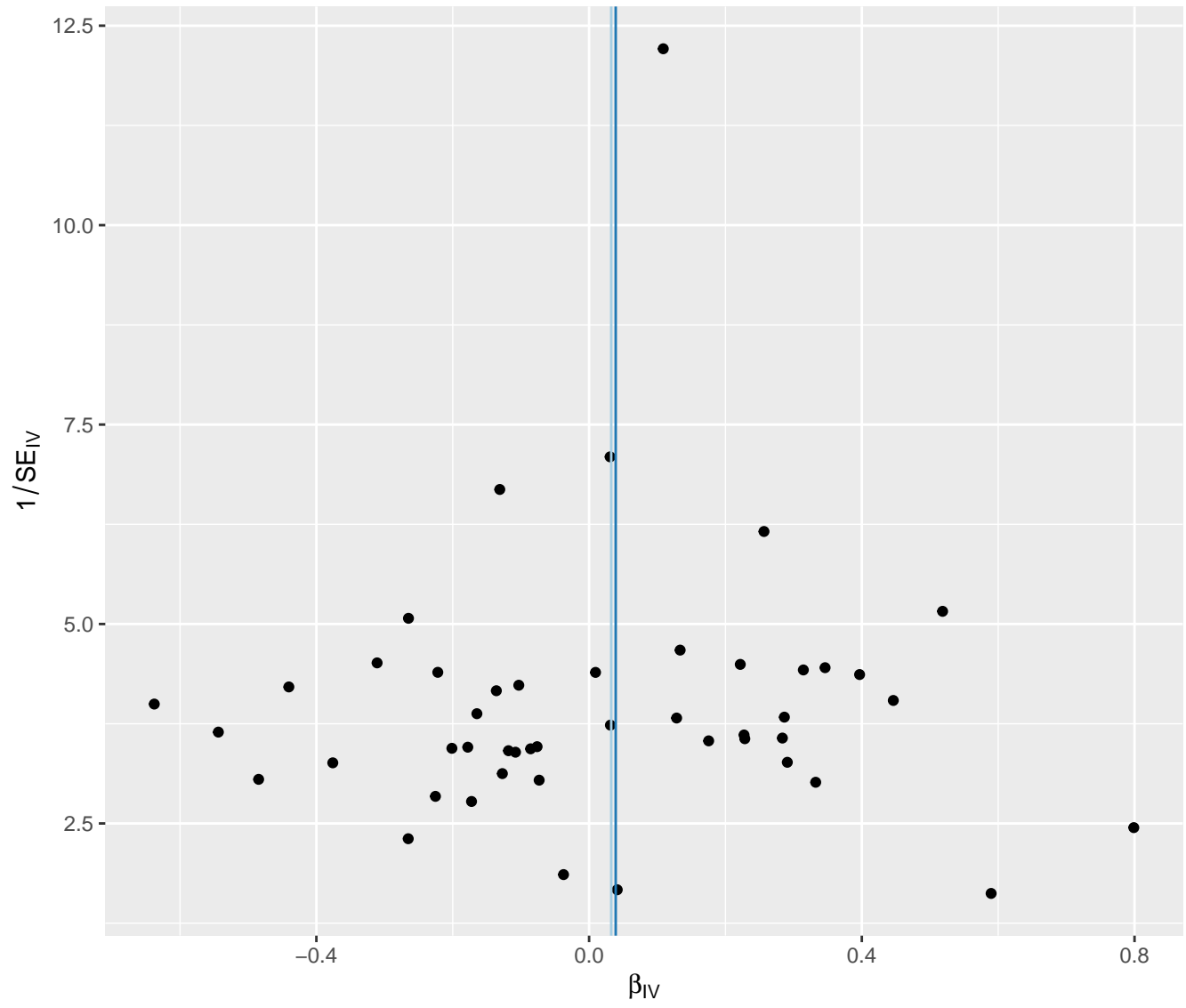

Supplement: Supplementary file 1 [file DataSheet_1.zip › supplementary material/CIM-OSTEOPOROSIS /GCST90274771.csv_funnel.pdf]

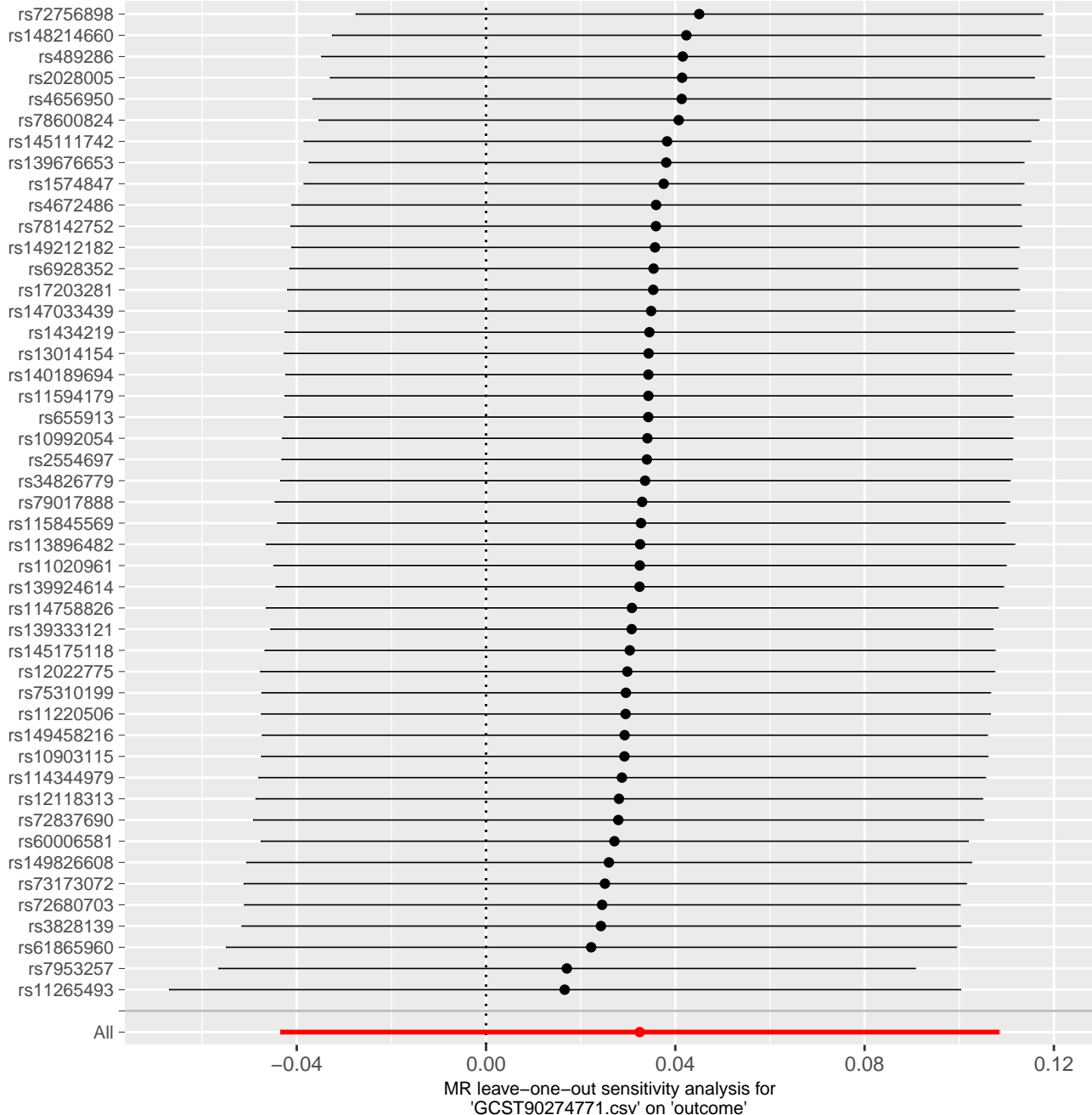

Supplement: Supplementary file 1 [file DataSheet_1.zip › supplementary material/CIM-OSTEOPOROSIS /GCST90274771.csv_leave_one_out.pdf]

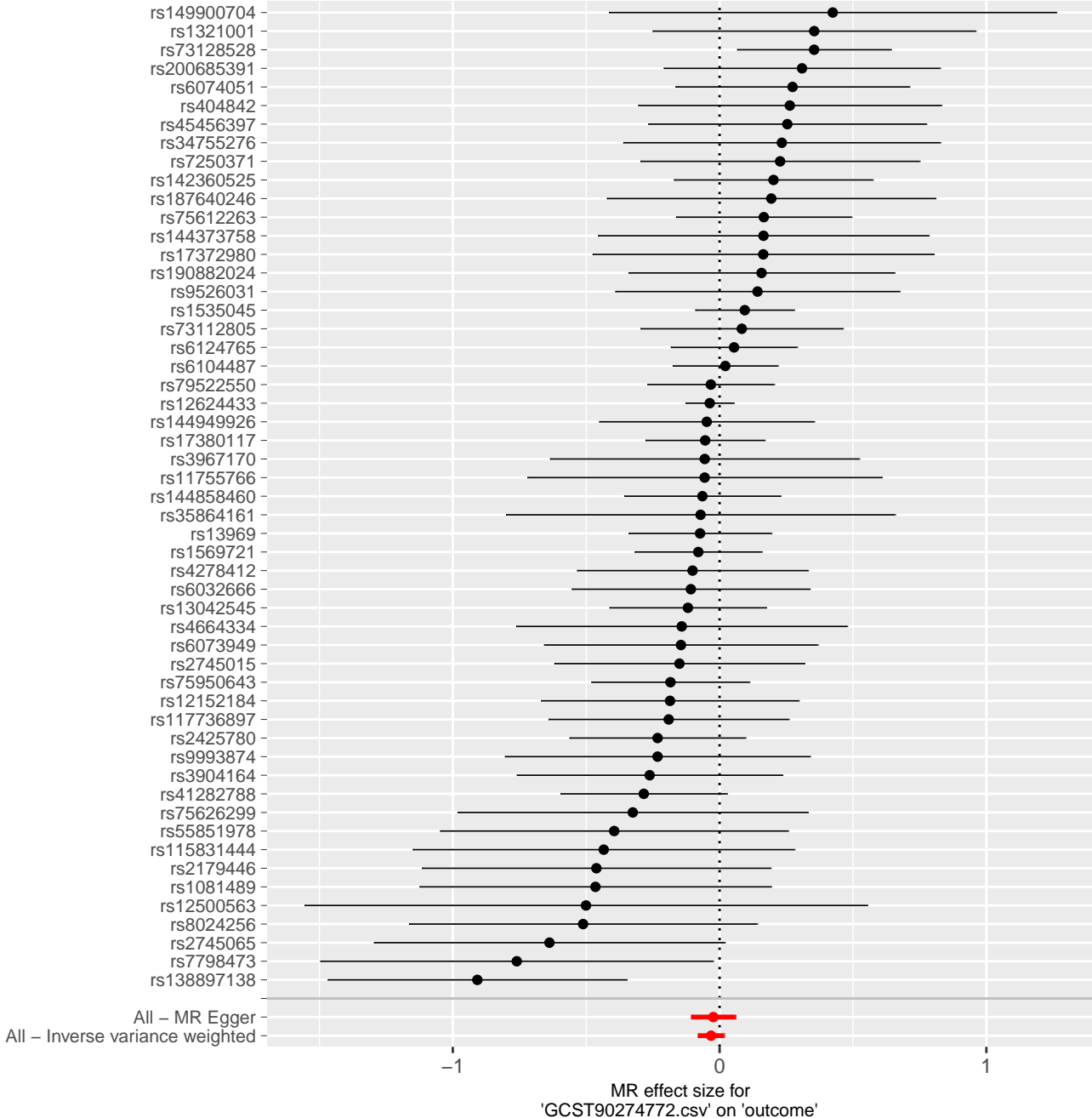

Supplement: Supplementary file 1 [file DataSheet_1.zip › supplementary material/CIM-OSTEOPOROSIS /GCST90274772.csv_forest.pdf]

# MR Method

- Inverse variance weighted
- MR Egger

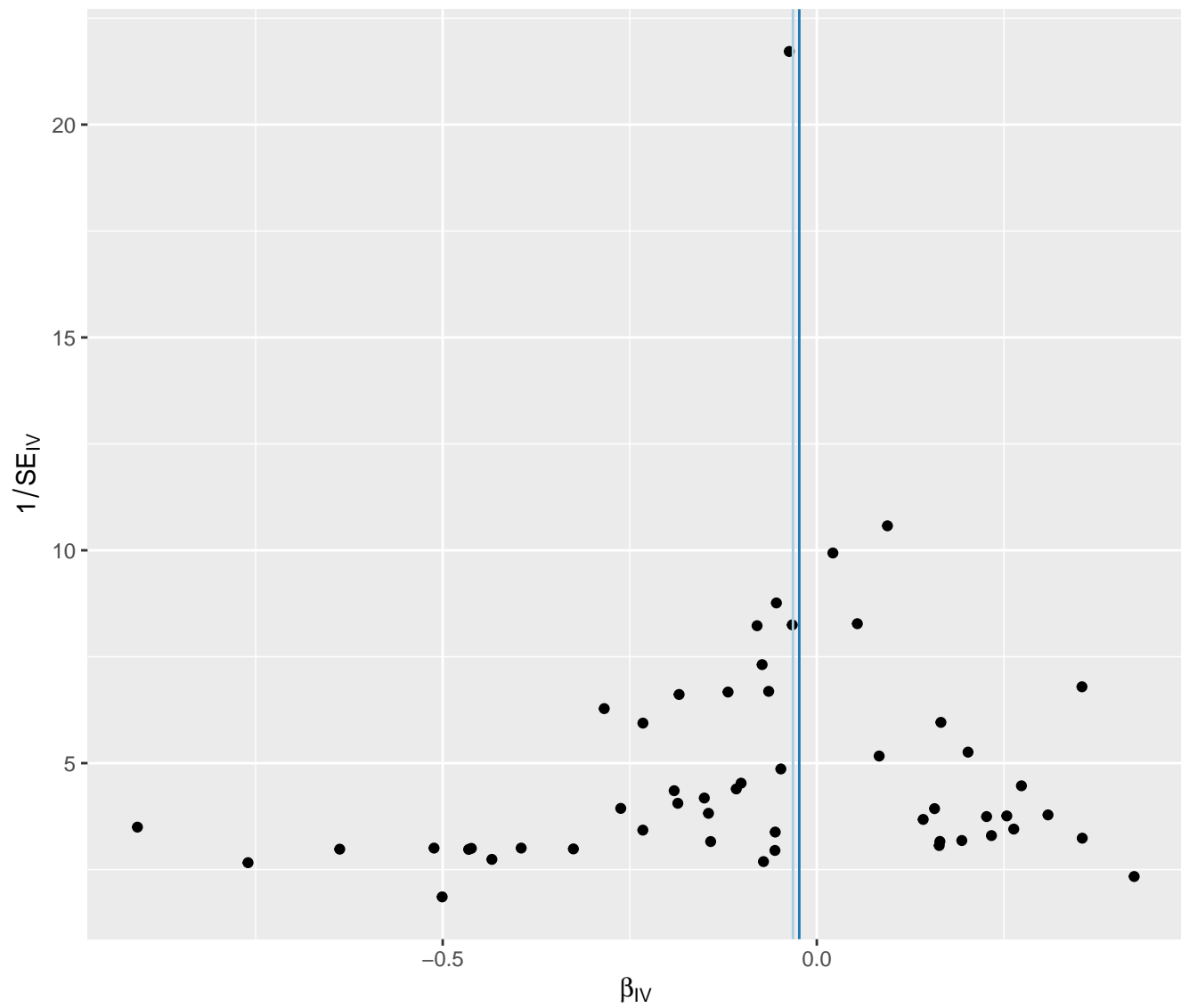

Supplement: Supplementary file 1 [file DataSheet_1.zip › supplementary material/CIM-OSTEOPOROSIS /GCST90274772.csv_funnel.pdf]

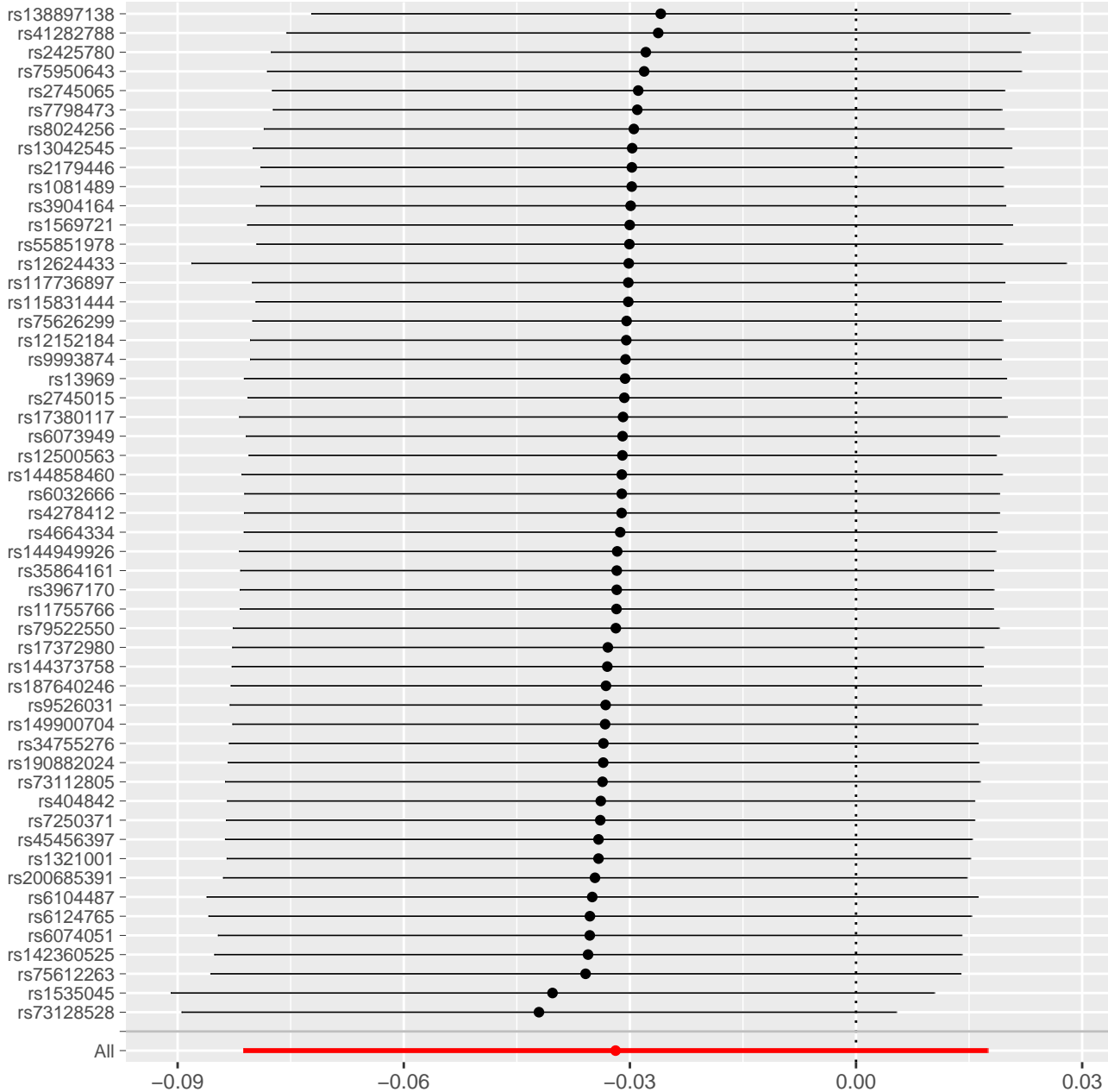

Supplement: Supplementary file 1 [file DataSheet_1.zip › supplementary material/CIM-OSTEOPOROSIS /GCST90274772.csv_leave_one_out.pdf]

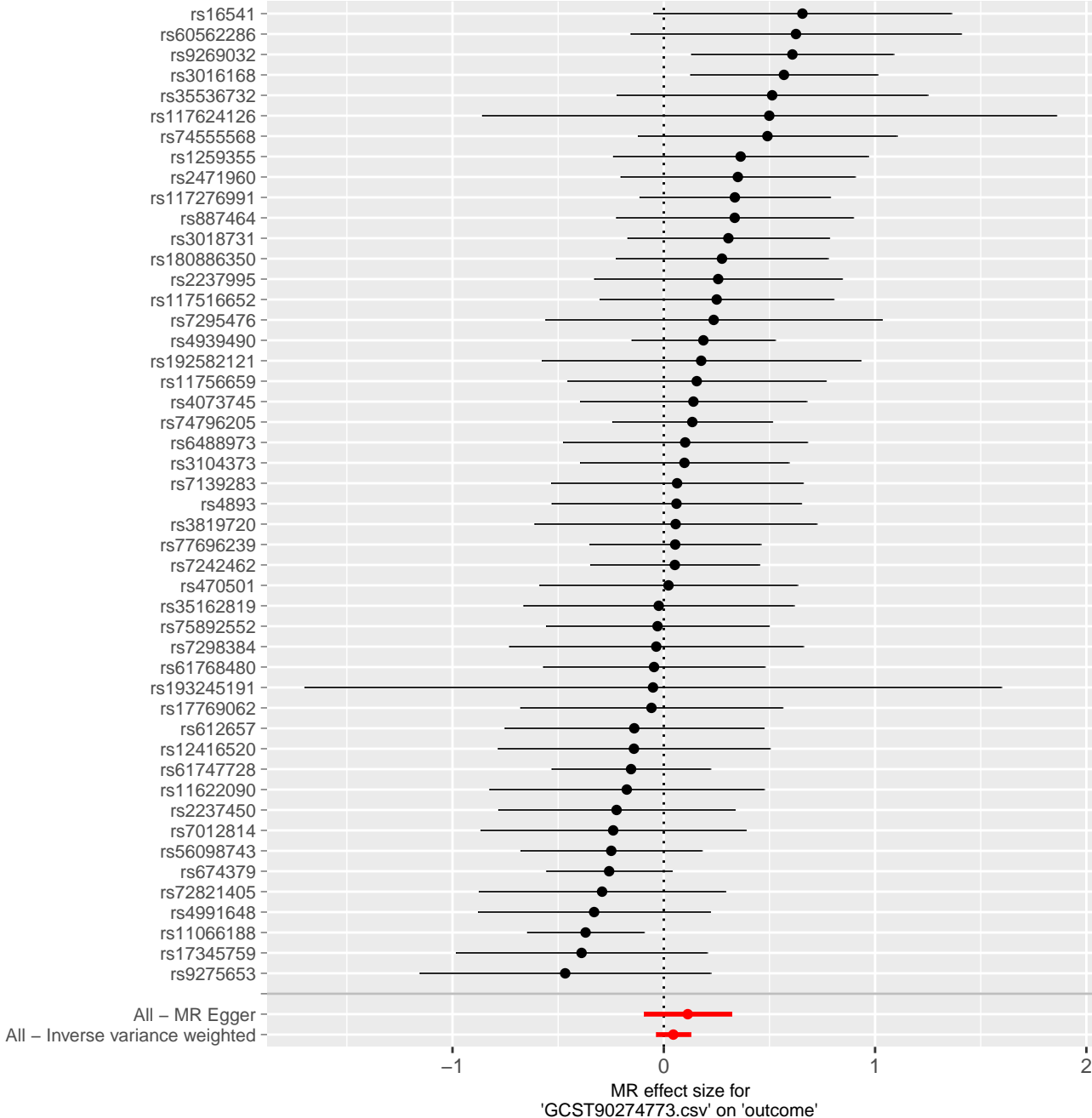

Supplement: Supplementary file 1 [file DataSheet_1.zip › supplementary material/CIM-OSTEOPOROSIS /GCST90274773.csv_forest.pdf]

# MR Method

- Inverse variance weighted
- MR Egger

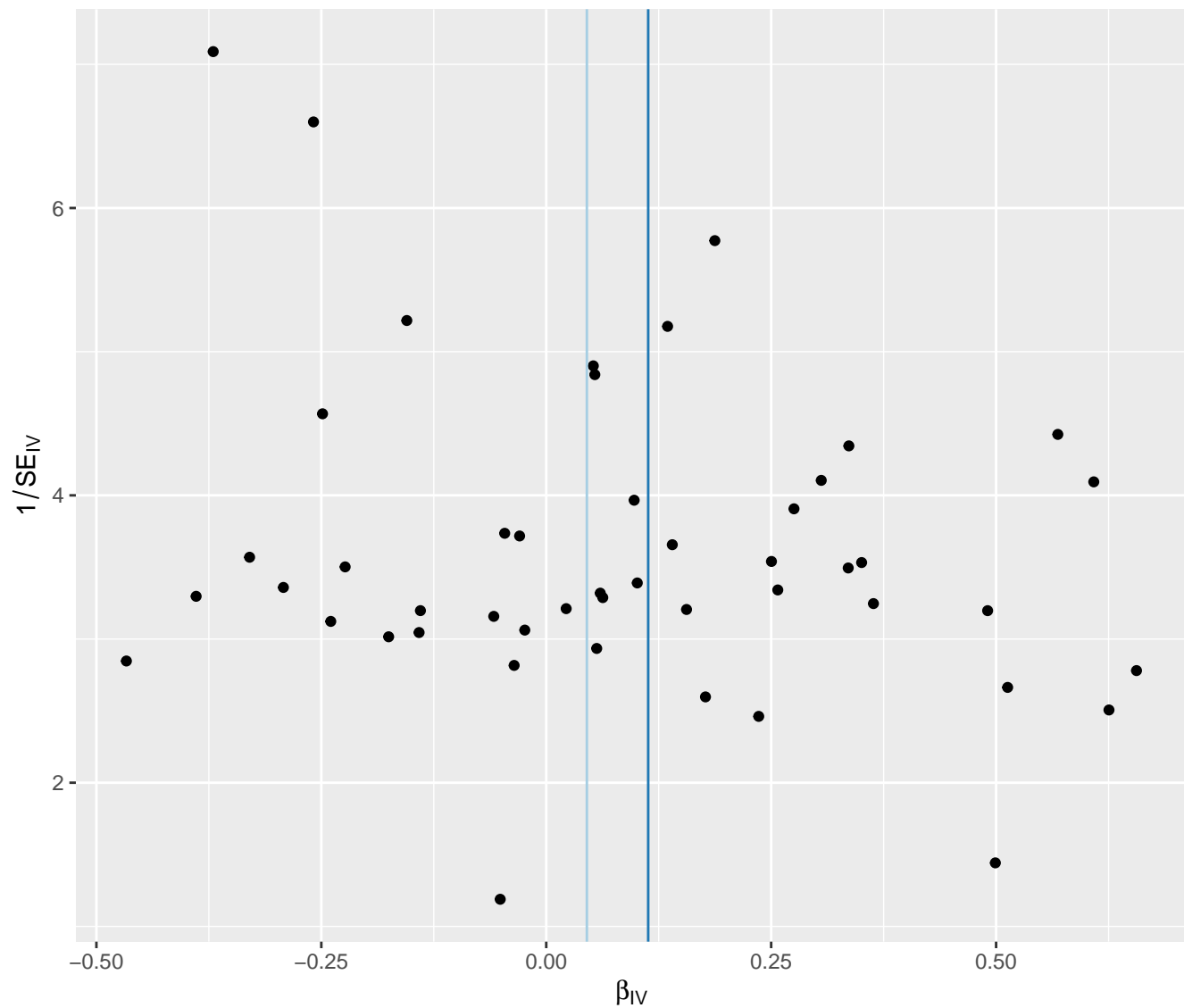

Supplement: Supplementary file 1 [file DataSheet_1.zip › supplementary material/CIM-OSTEOPOROSIS /GCST90274773.csv_funnel.pdf]

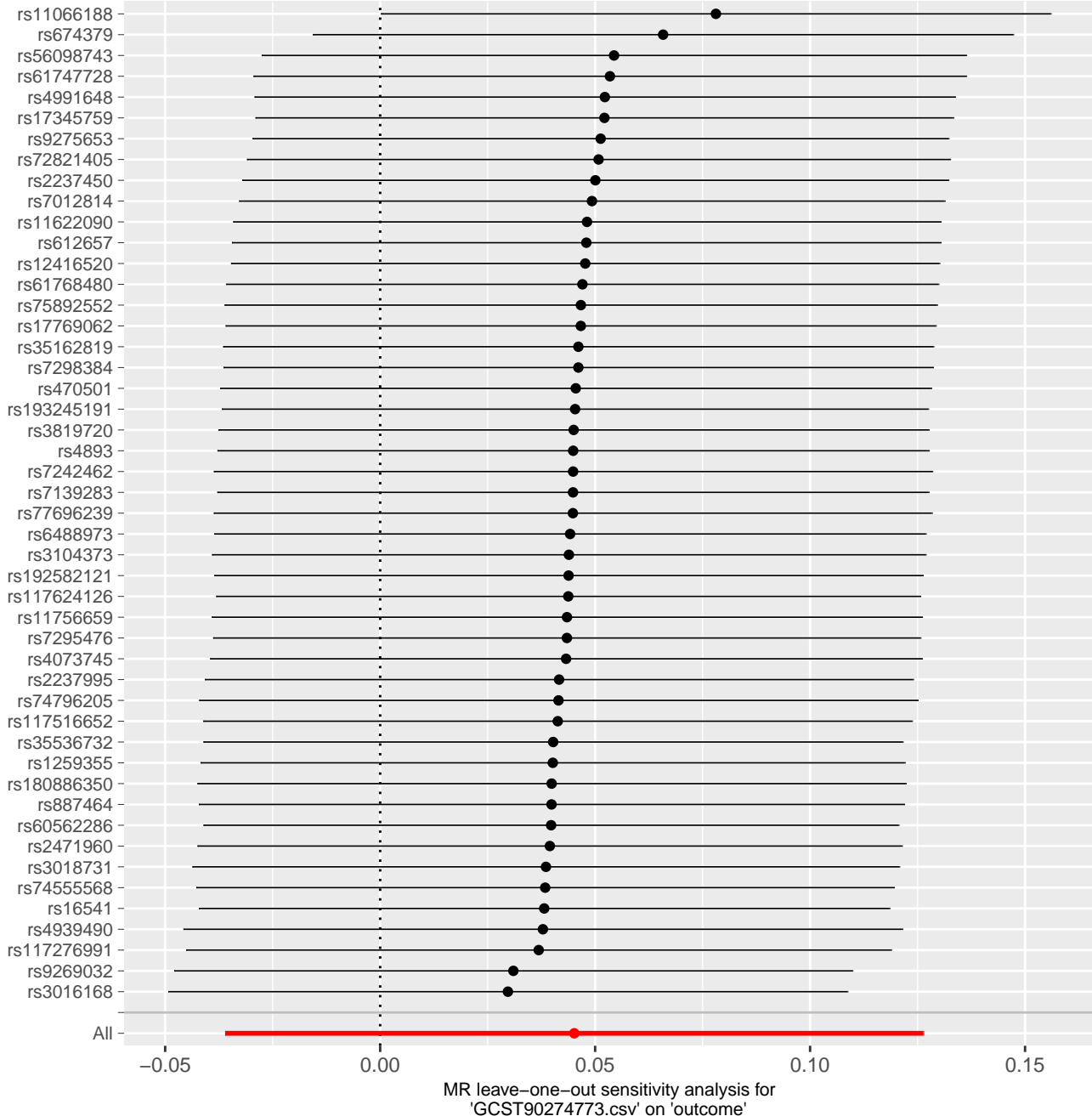

Supplement: Supplementary file 1 [file DataSheet_1.zip › supplementary material/CIM-OSTEOPOROSIS /GCST90274773.csv_leave_one_out.pdf]

All – MR Egger  
All – Inverse variance weighted

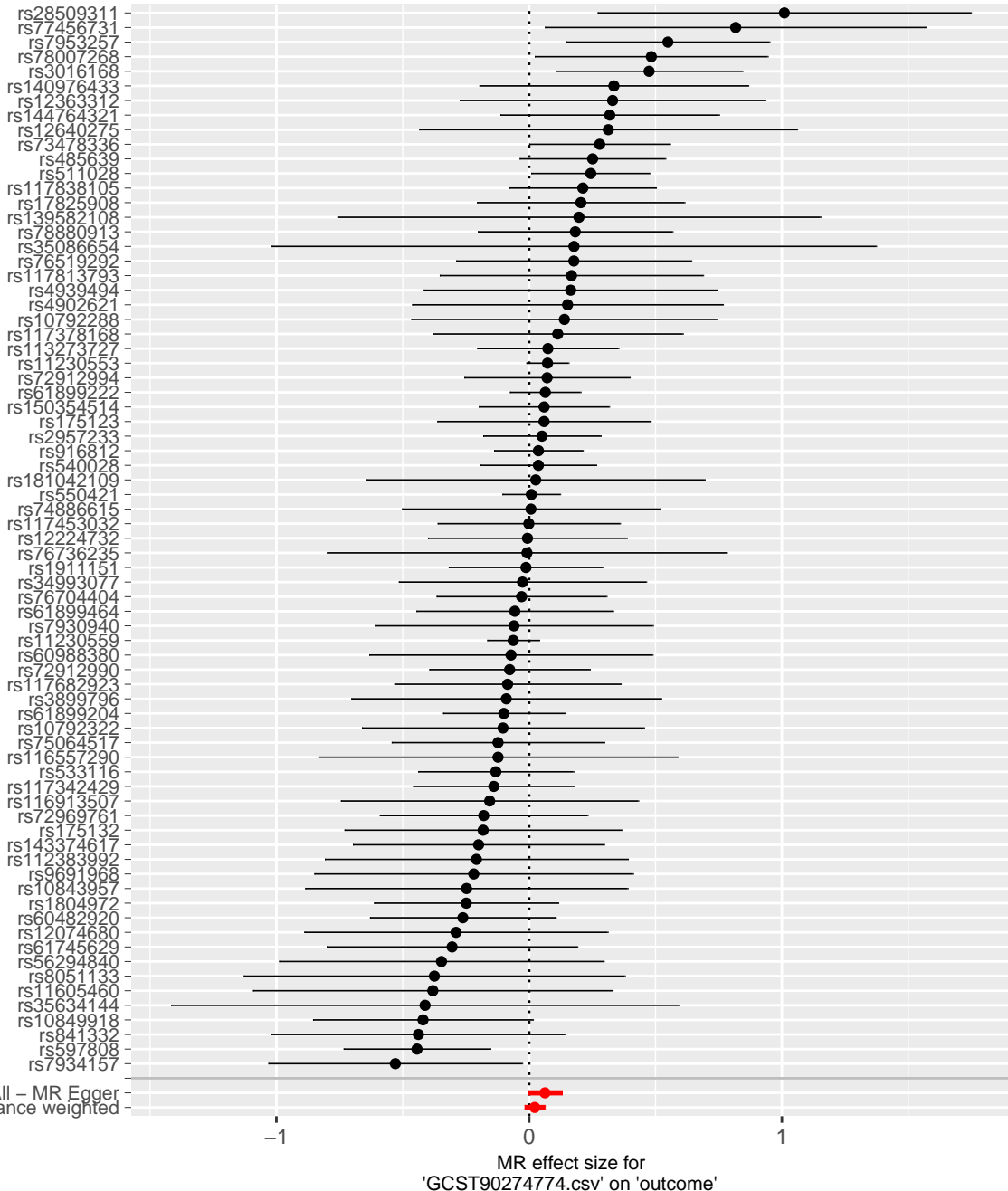

Supplement: Supplementary file 1 [file DataSheet_1.zip › supplementary material/CIM-OSTEOPOROSIS /GCST90274774.csv_forest.pdf]

# MR Method

- Inverse variance weighted
- MR Egger

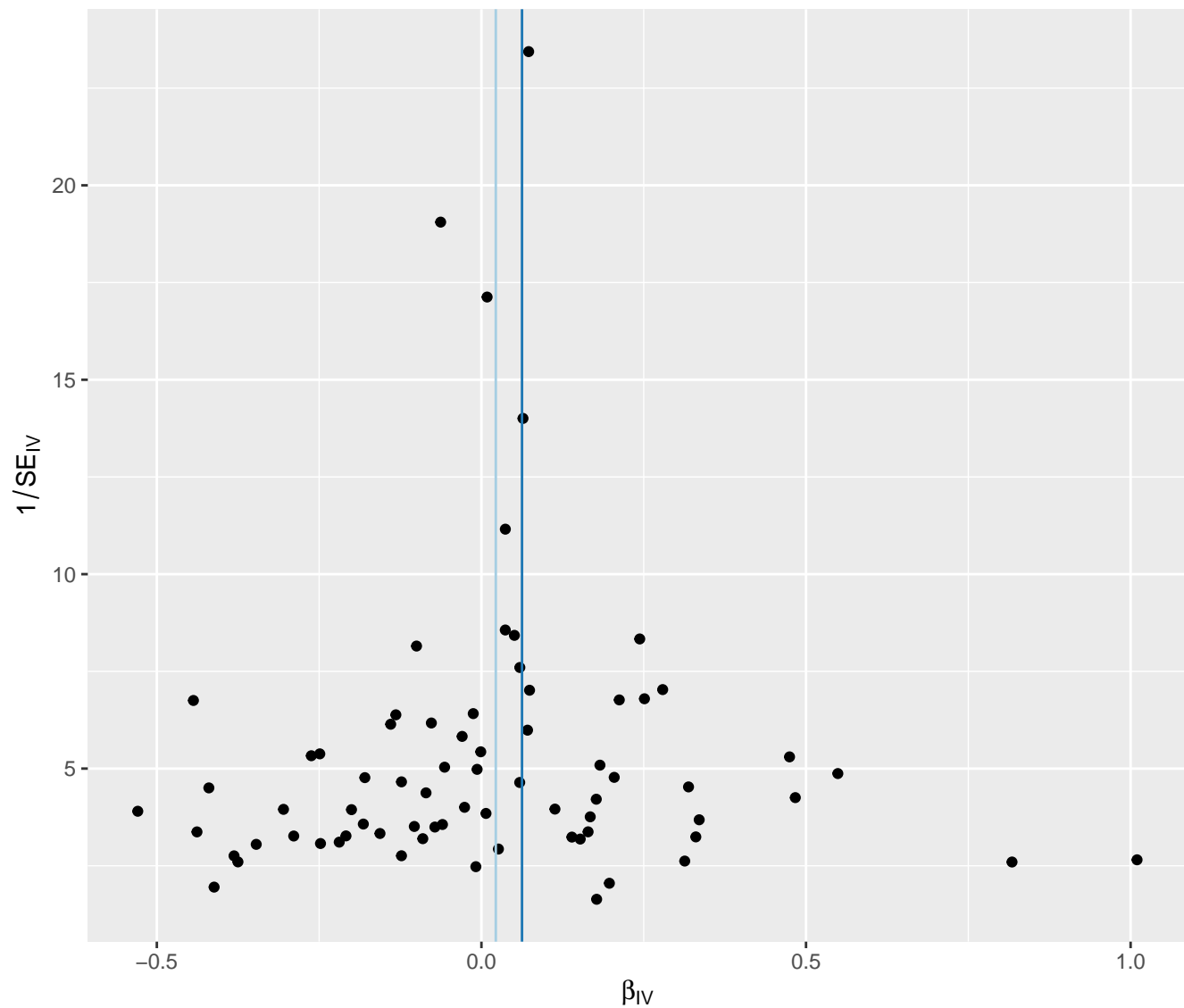

Supplement: Supplementary file 1 [file DataSheet_1.zip › supplementary material/CIM-OSTEOPOROSIS /GCST90274774.csv_funnel.pdf]

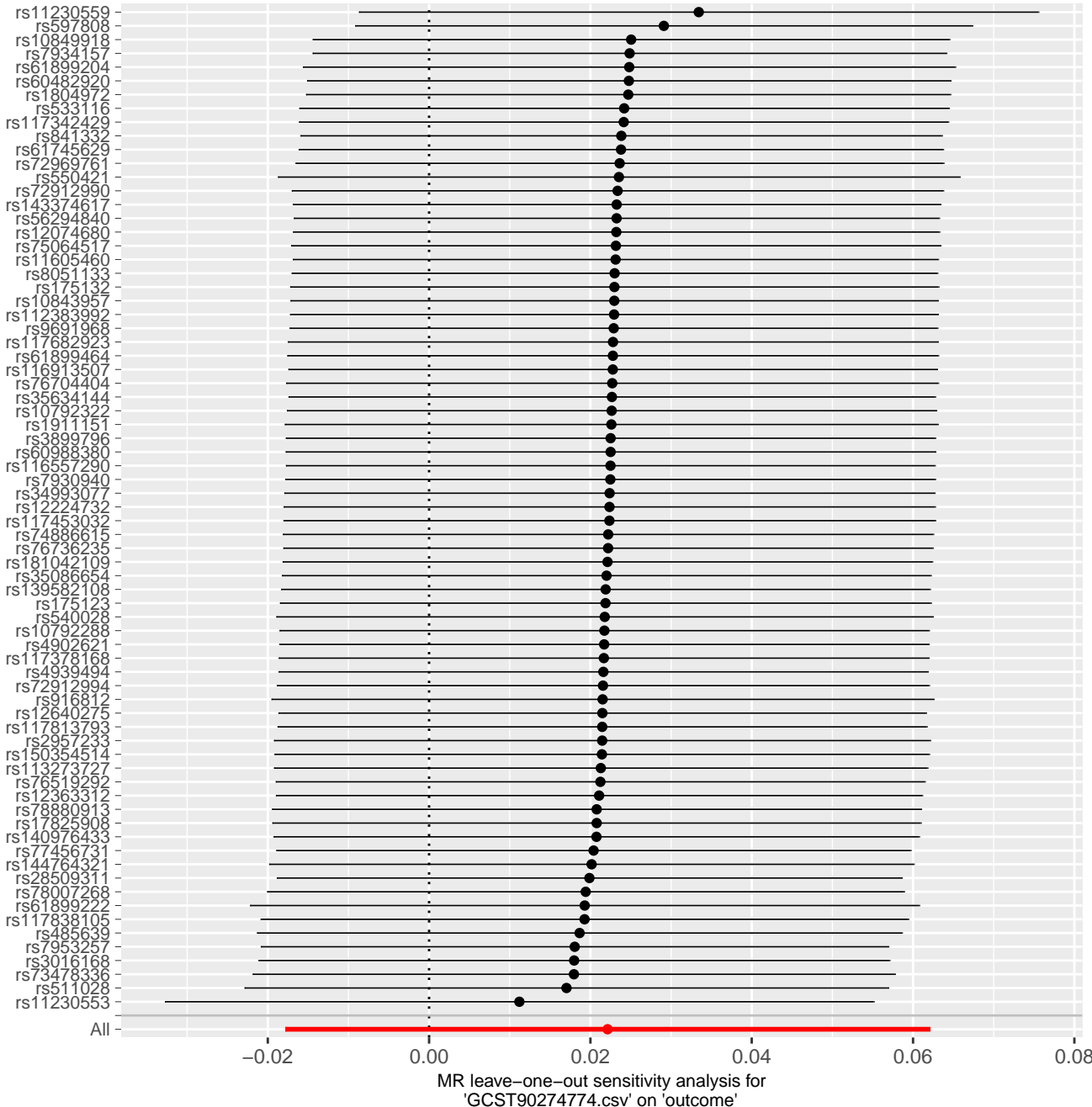

Supplement: Supplementary file 1 [file DataSheet_1.zip › supplementary material/CIM-OSTEOPOROSIS /GCST90274774.csv_leave_one_out.pdf]

# MR Test

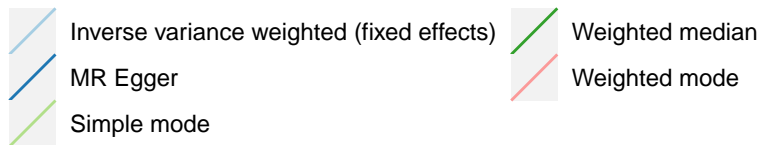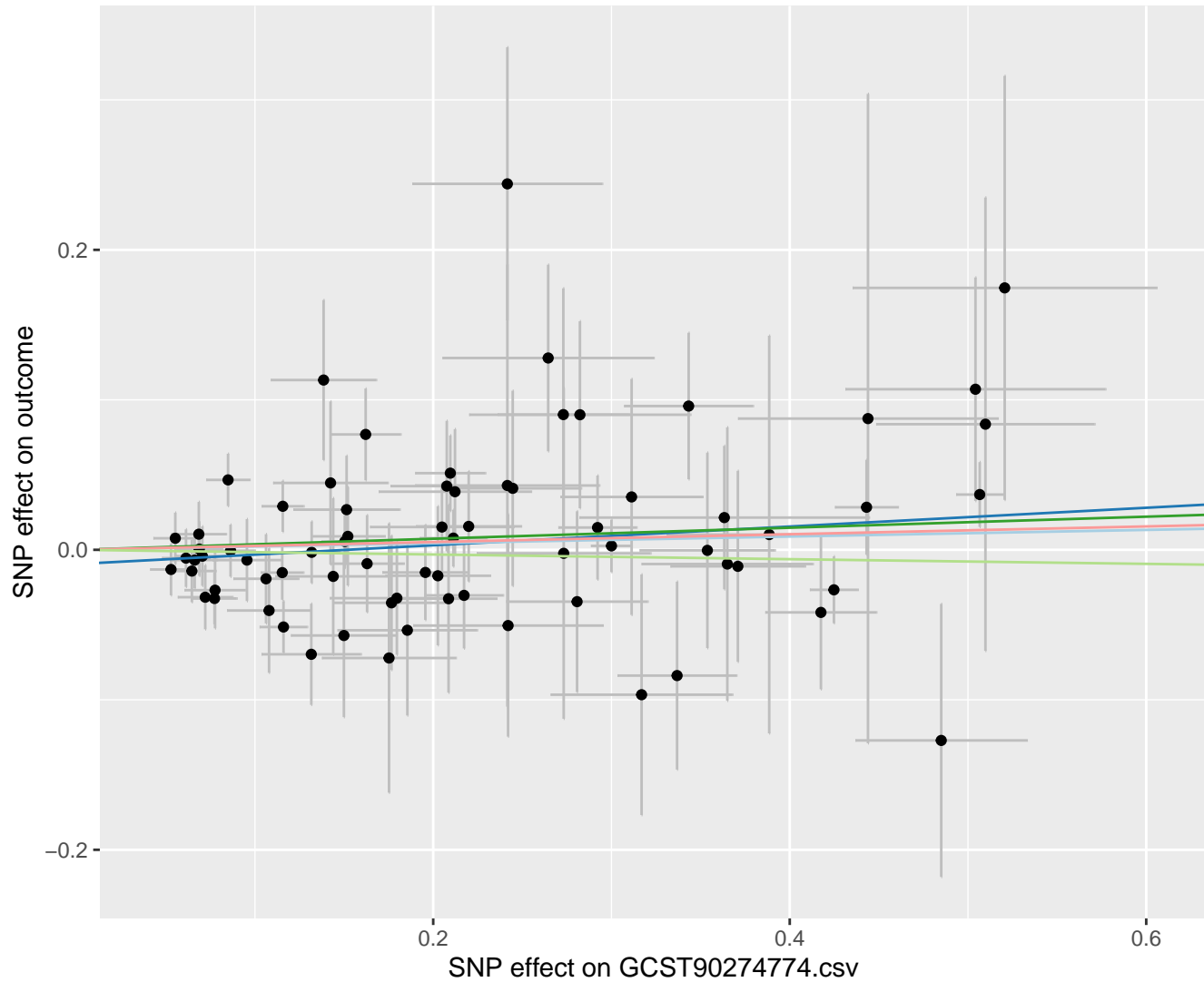

Supplement: Supplementary file 1 [file DataSheet_1.zip › supplementary material/CIM-OSTEOPOROSIS /GCST90274774.csv_scatter.pdf]

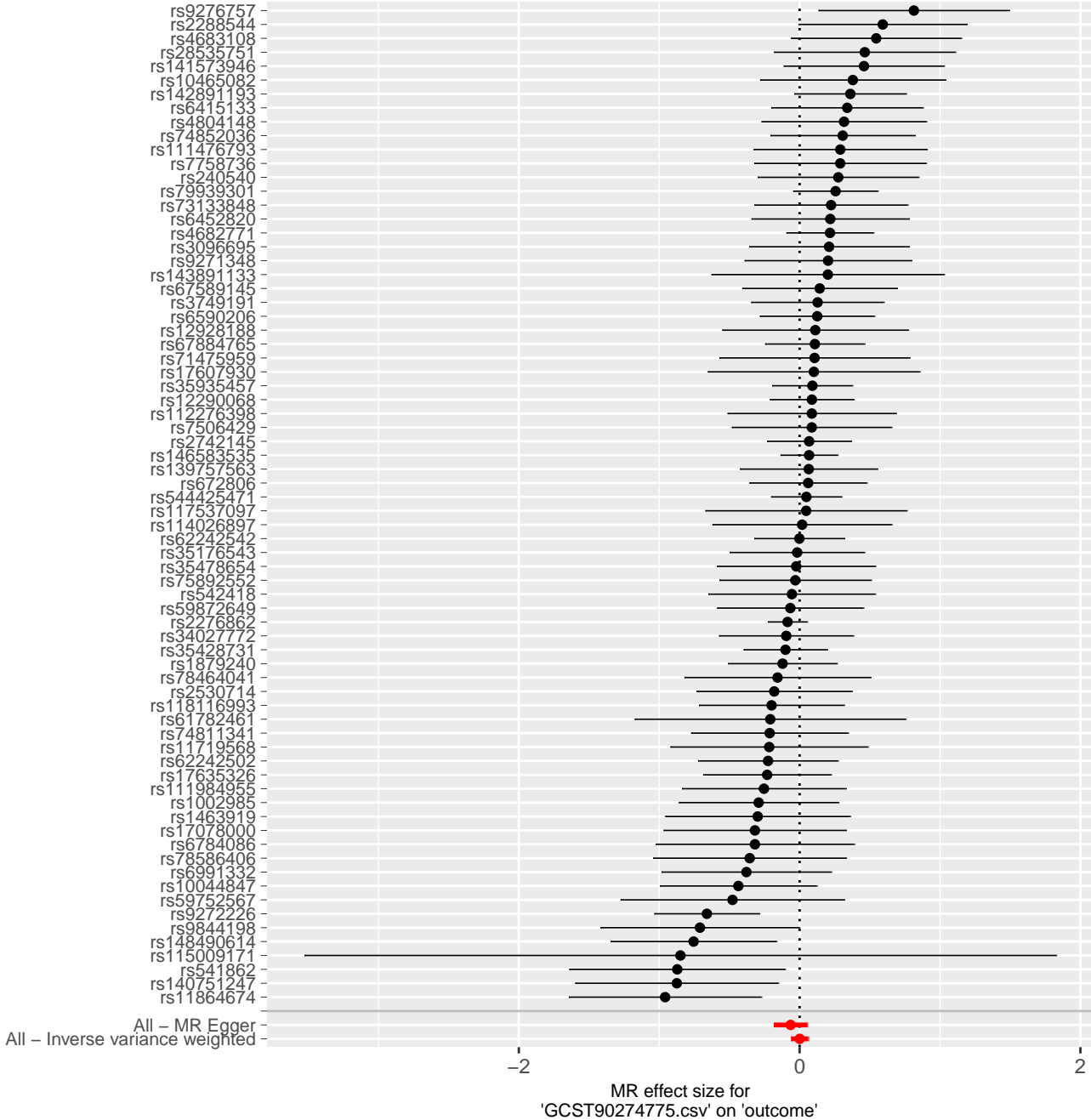

Supplement: Supplementary file 1 [file DataSheet_1.zip › supplementary material/CIM-OSTEOPOROSIS /GCST90274775.csv_forest.pdf]

# MR Method

- Inverse variance weighted
- MR Egger

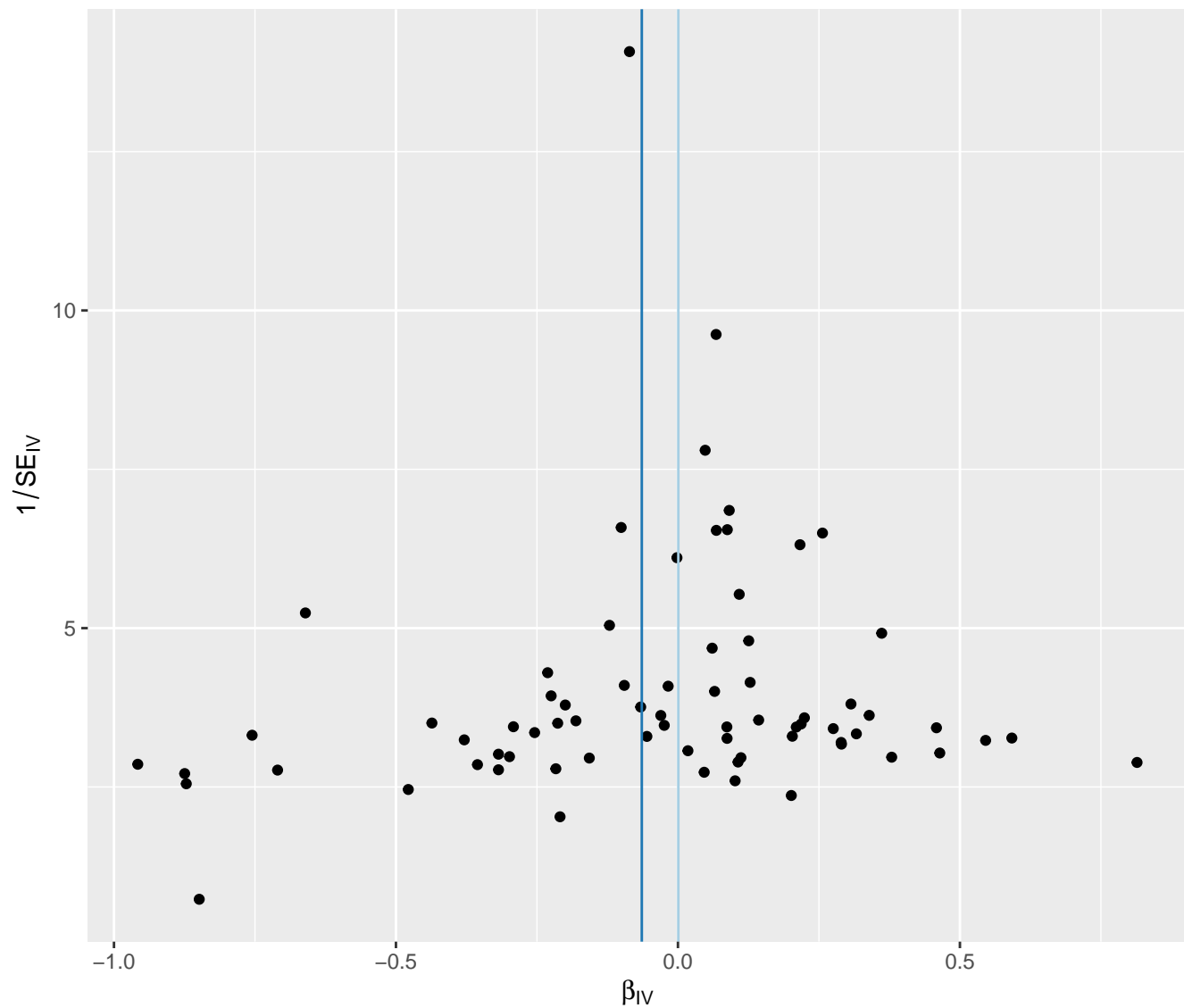

Supplement: Supplementary file 1 [file DataSheet_1.zip › supplementary material/CIM-OSTEOPOROSIS /GCST90274775.csv_funnel.pdf]

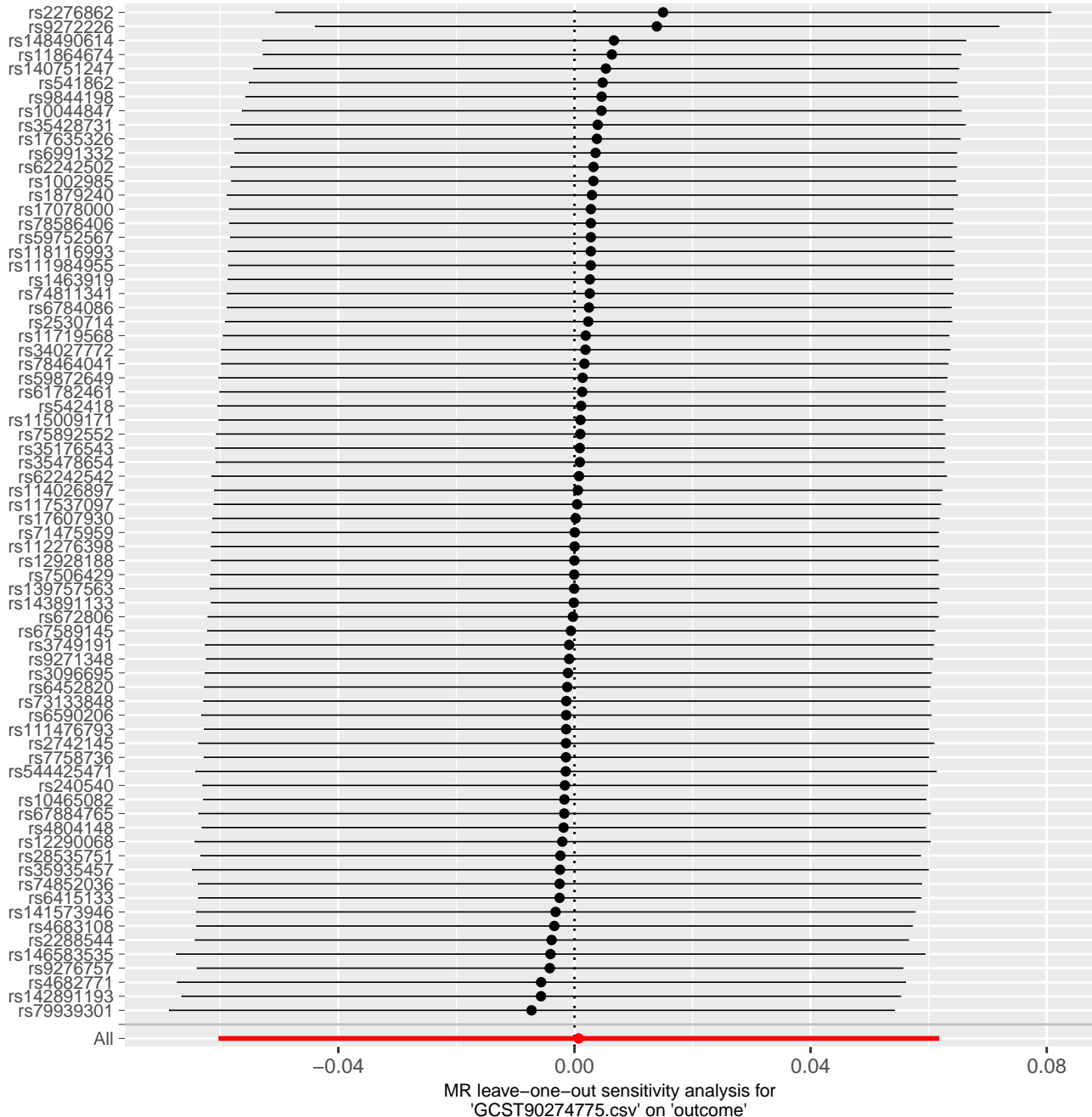

Supplement: Supplementary file 1 [file DataSheet_1.zip › supplementary material/CIM-OSTEOPOROSIS /GCST90274775.csv_leave_one_out.pdf]

# MR Test

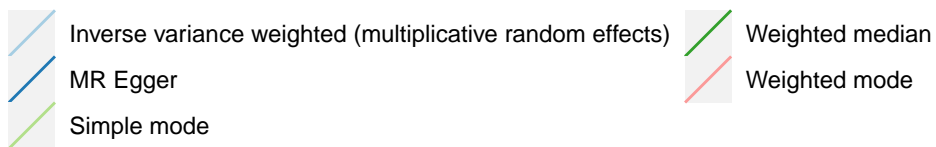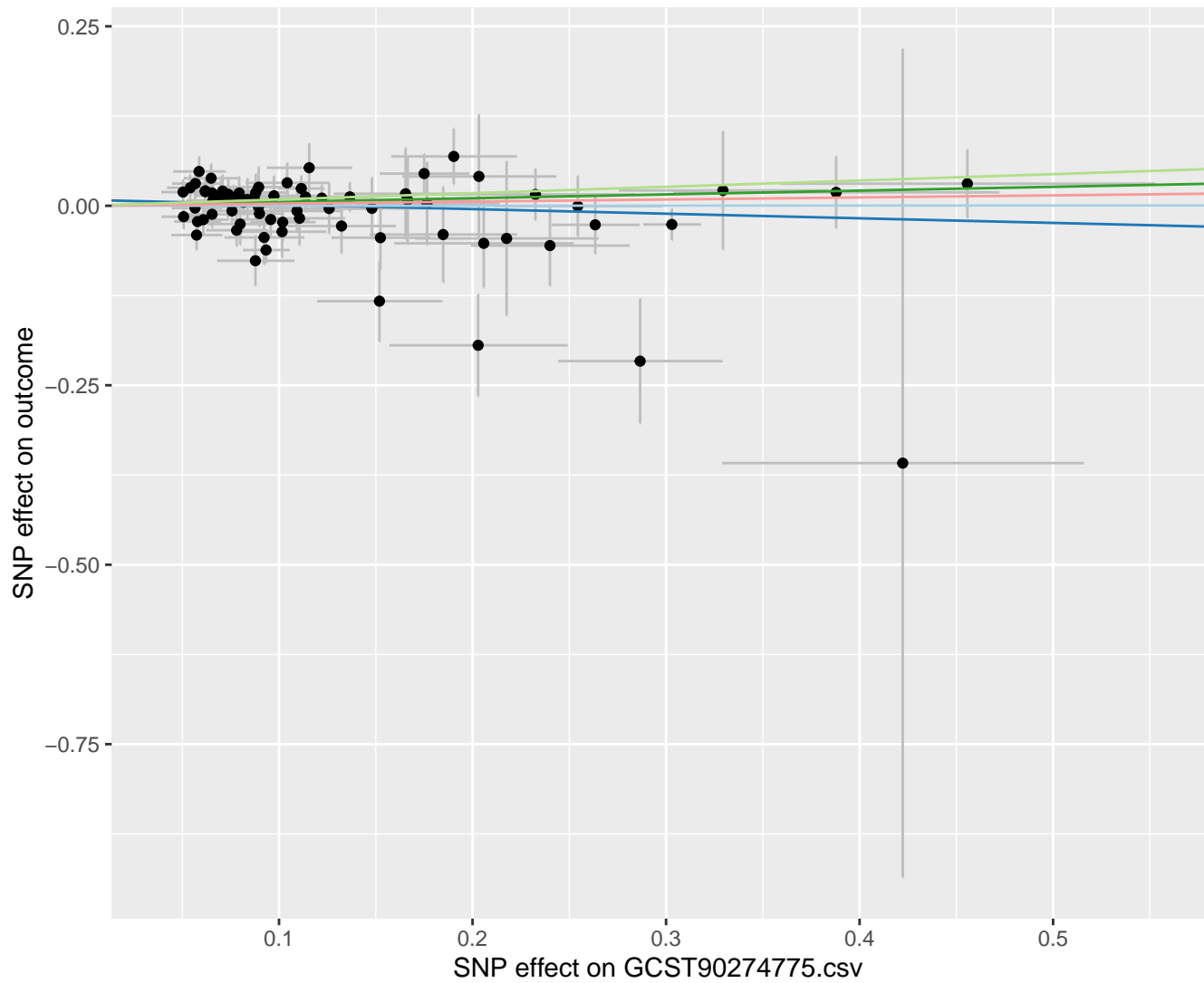

Supplement: Supplementary file 1 [file DataSheet_1.zip › supplementary material/CIM-OSTEOPOROSIS /GCST90274775.csv_scatter.pdf]

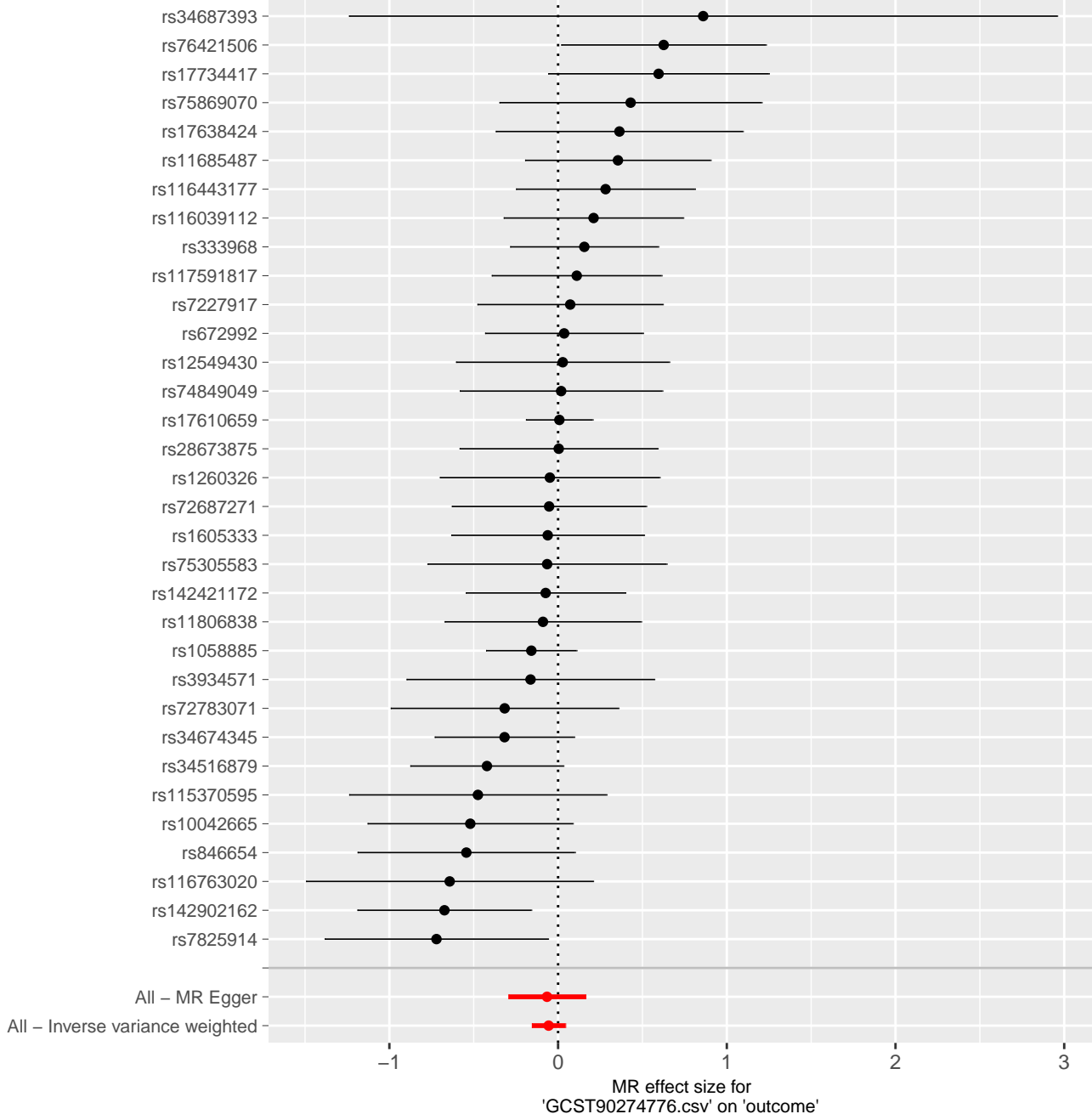

Supplement: Supplementary file 1 [file DataSheet_1.zip › supplementary material/CIM-OSTEOPOROSIS /GCST90274776.csv_forest.pdf]

# MR Method

- Inverse variance weighted
- MR Egger

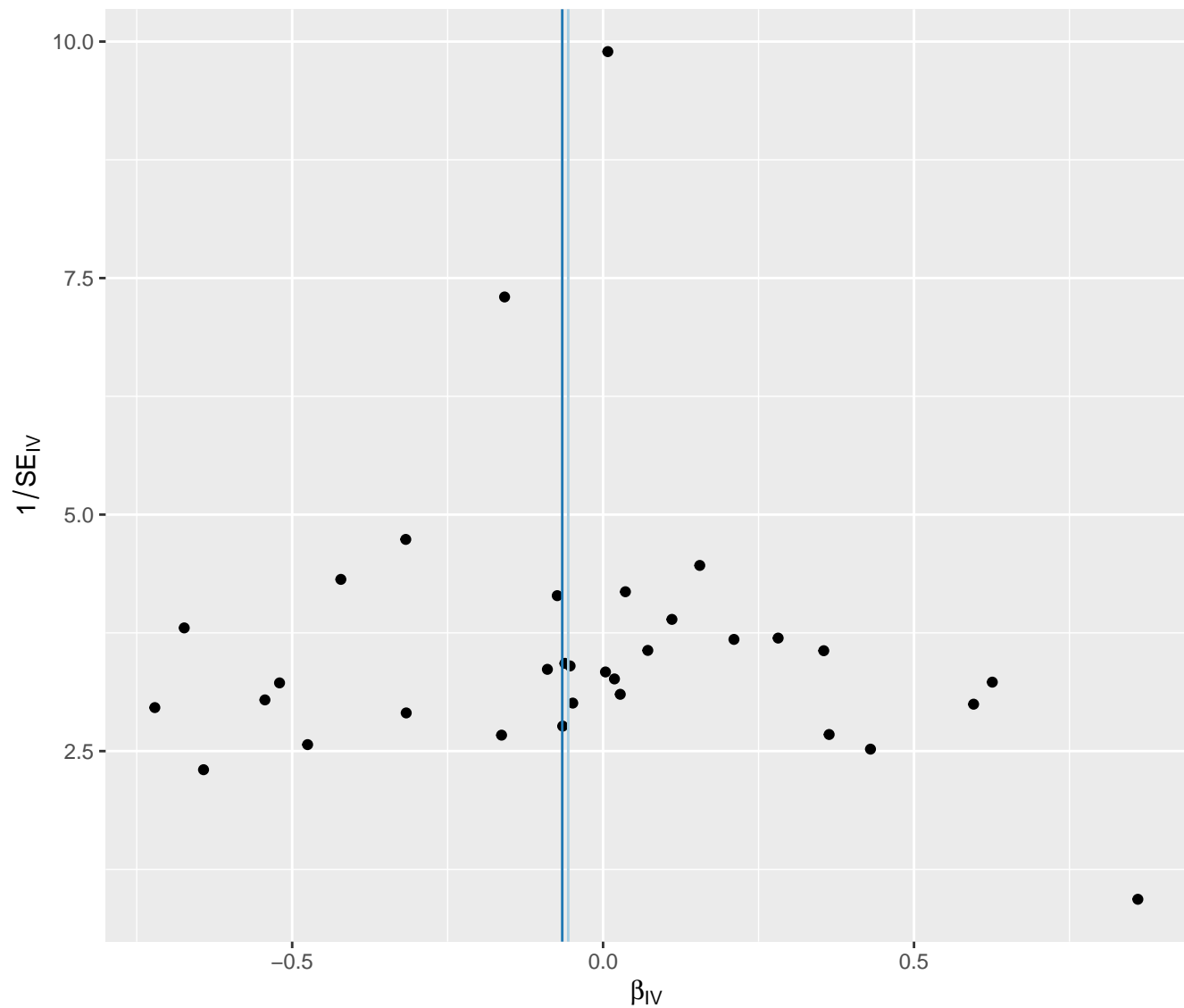

Supplement: Supplementary file 1 [file DataSheet_1.zip › supplementary material/CIM-OSTEOPOROSIS /GCST90274776.csv_funnel.pdf]

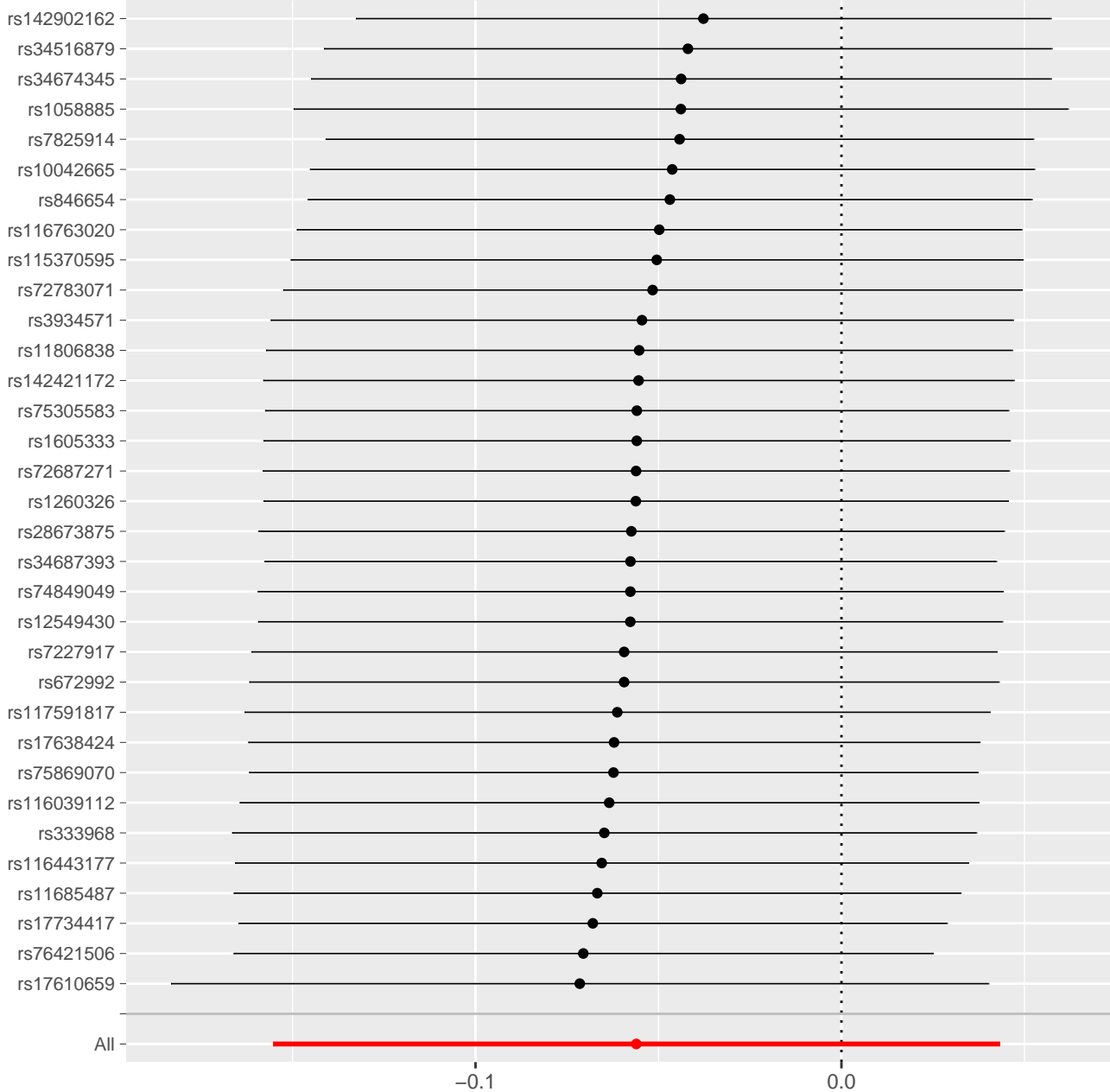

MR leave-one-out sensitivity analysis for  
'GCST90274776.csv' on 'outcome'

Supplement: Supplementary file 1 [file DataSheet_1.zip › supplementary material/CIM-OSTEOPOROSIS /GCST90274776.csv_leave_one_out.pdf]

All – Inverse variance weighted

All – MR Egger

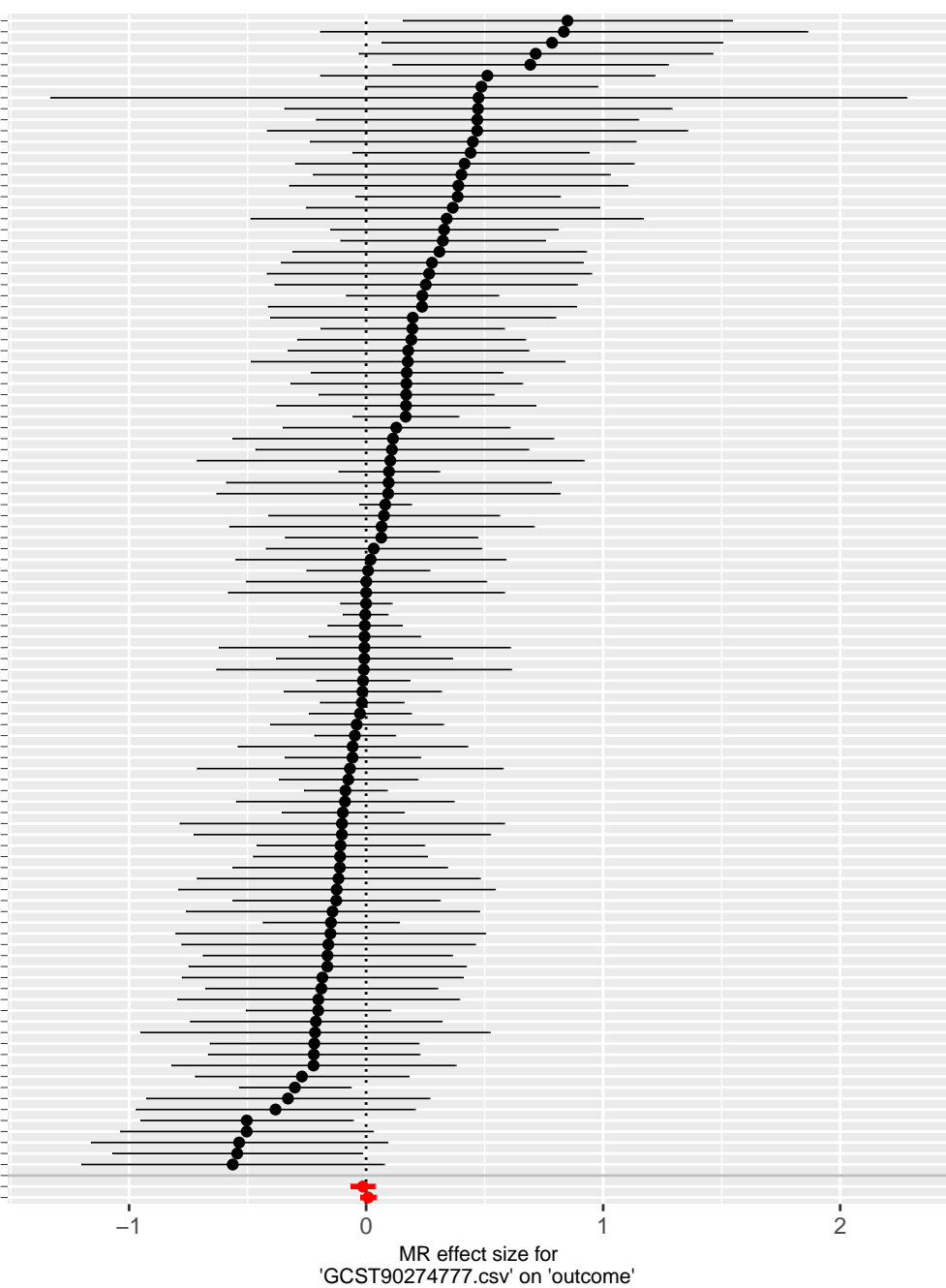

Supplement: Supplementary file 1 [file DataSheet_1.zip › supplementary material/CIM-OSTEOPOROSIS /GCST90274777.csv_forest.pdf]

# MR Method

- Inverse variance weighted
- MR Egger

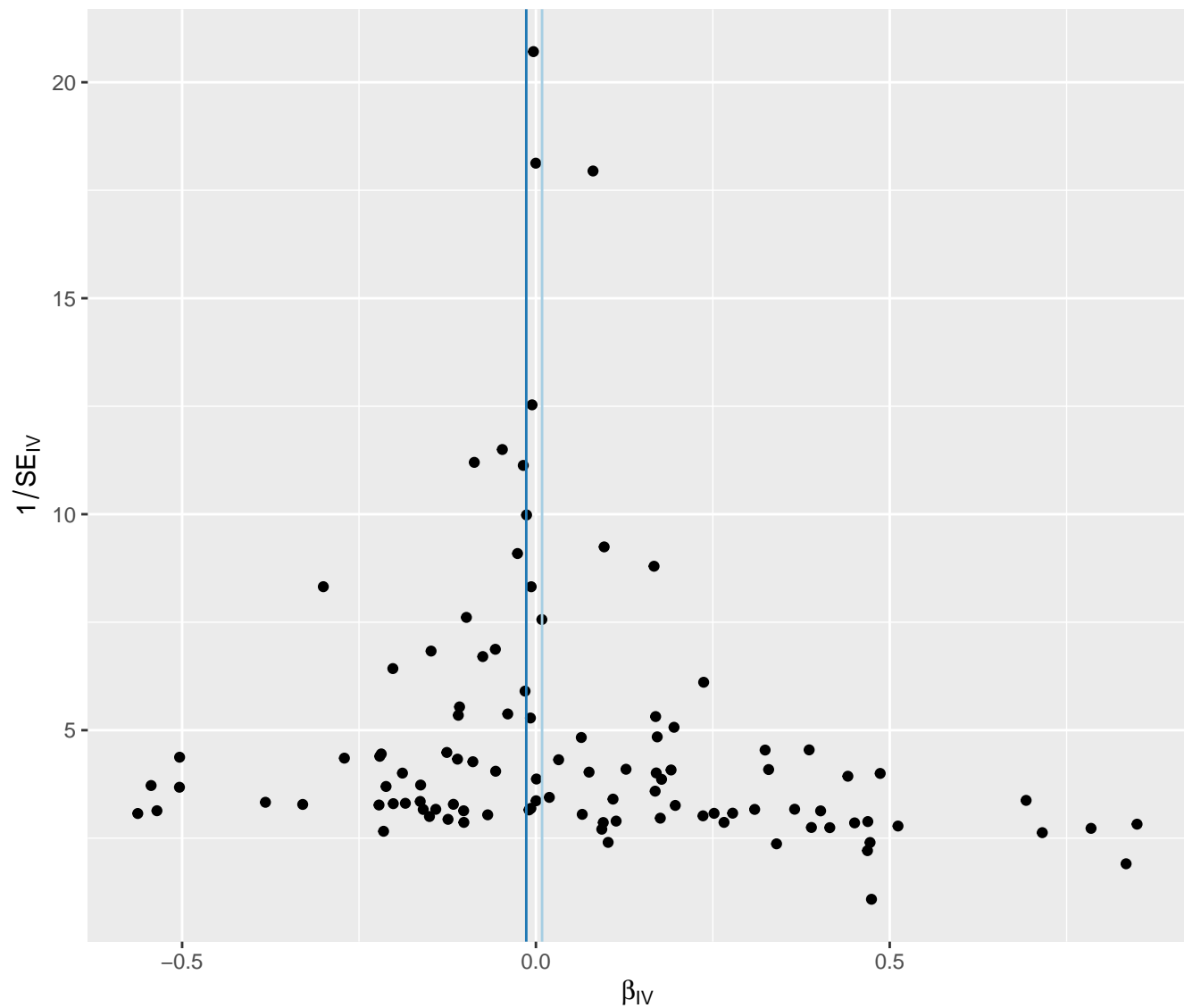

Supplement: Supplementary file 1 [file DataSheet_1.zip › supplementary material/CIM-OSTEOPOROSIS /GCST90274777.csv_funnel.pdf]

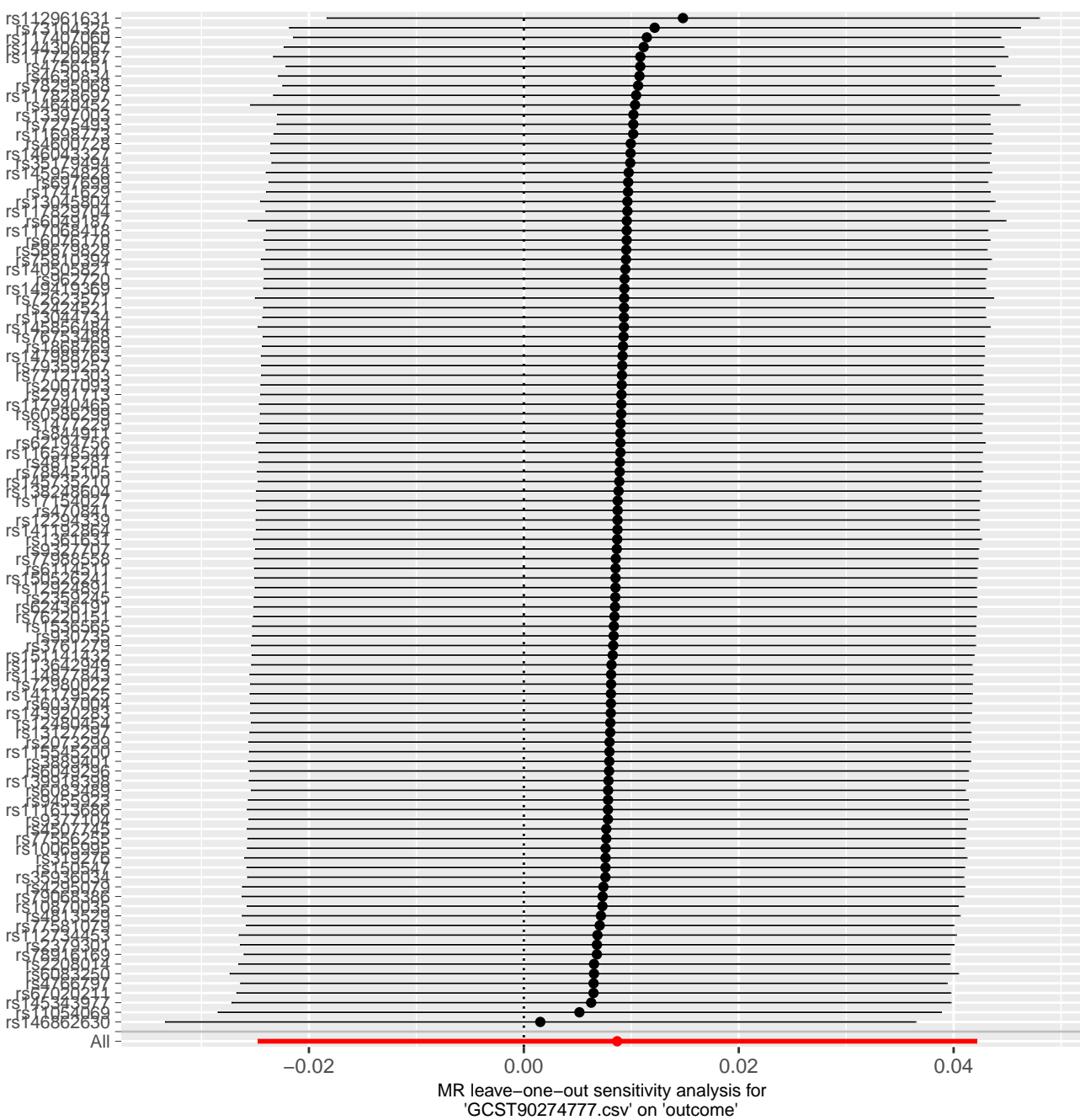

Supplement: Supplementary file 1 [file DataSheet_1.zip › supplementary material/CIM-OSTEOPOROSIS /GCST90274777.csv_leave_one_out.pdf]

# MR Test

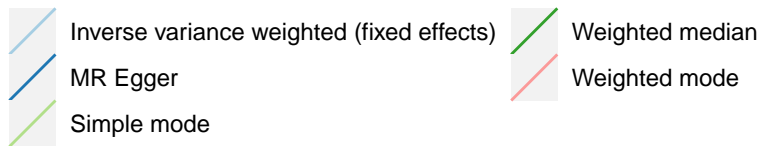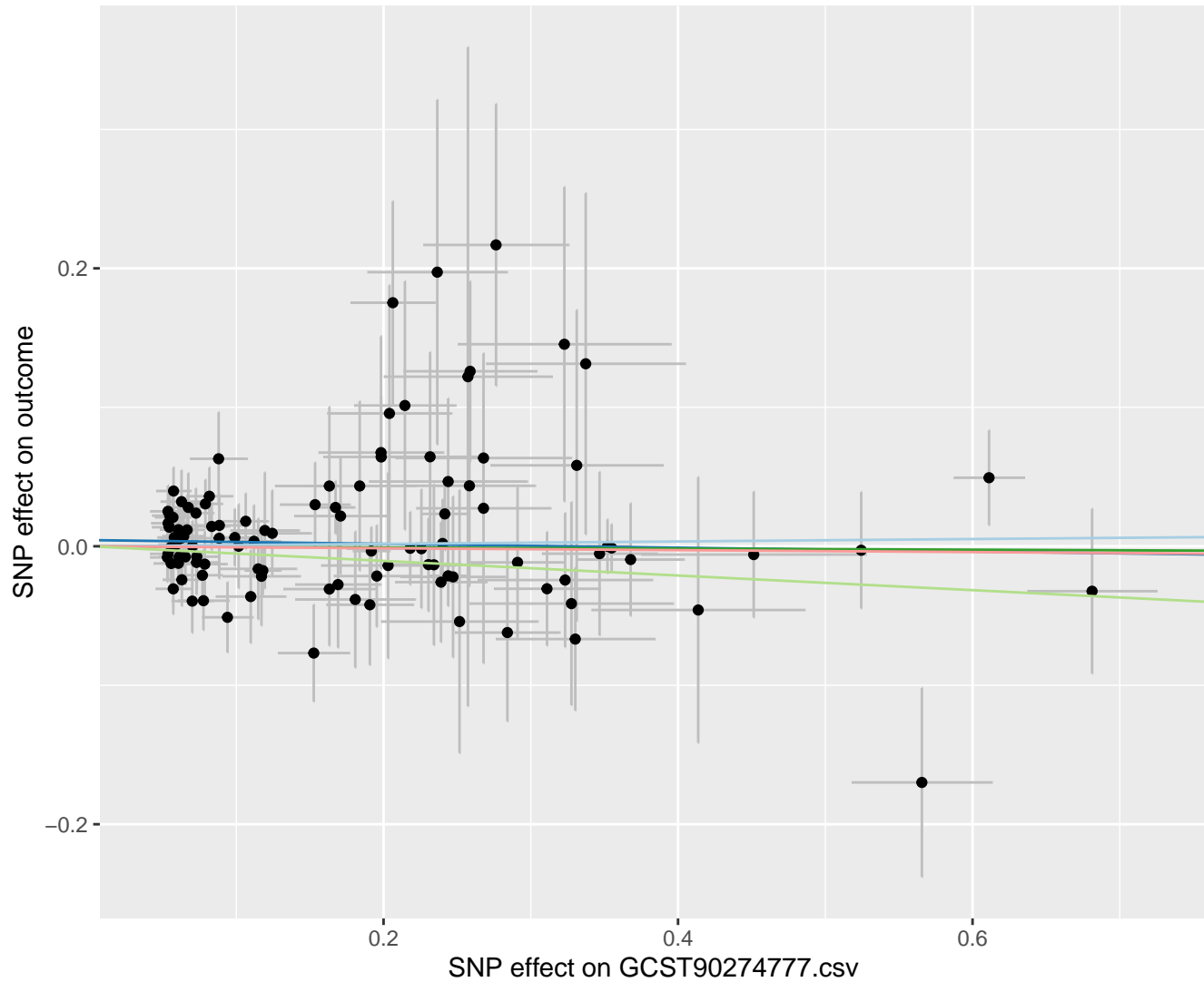

Supplement: Supplementary file 1 [file DataSheet_1.zip › supplementary material/CIM-OSTEOPOROSIS /GCST90274777.csv_scatter.pdf]

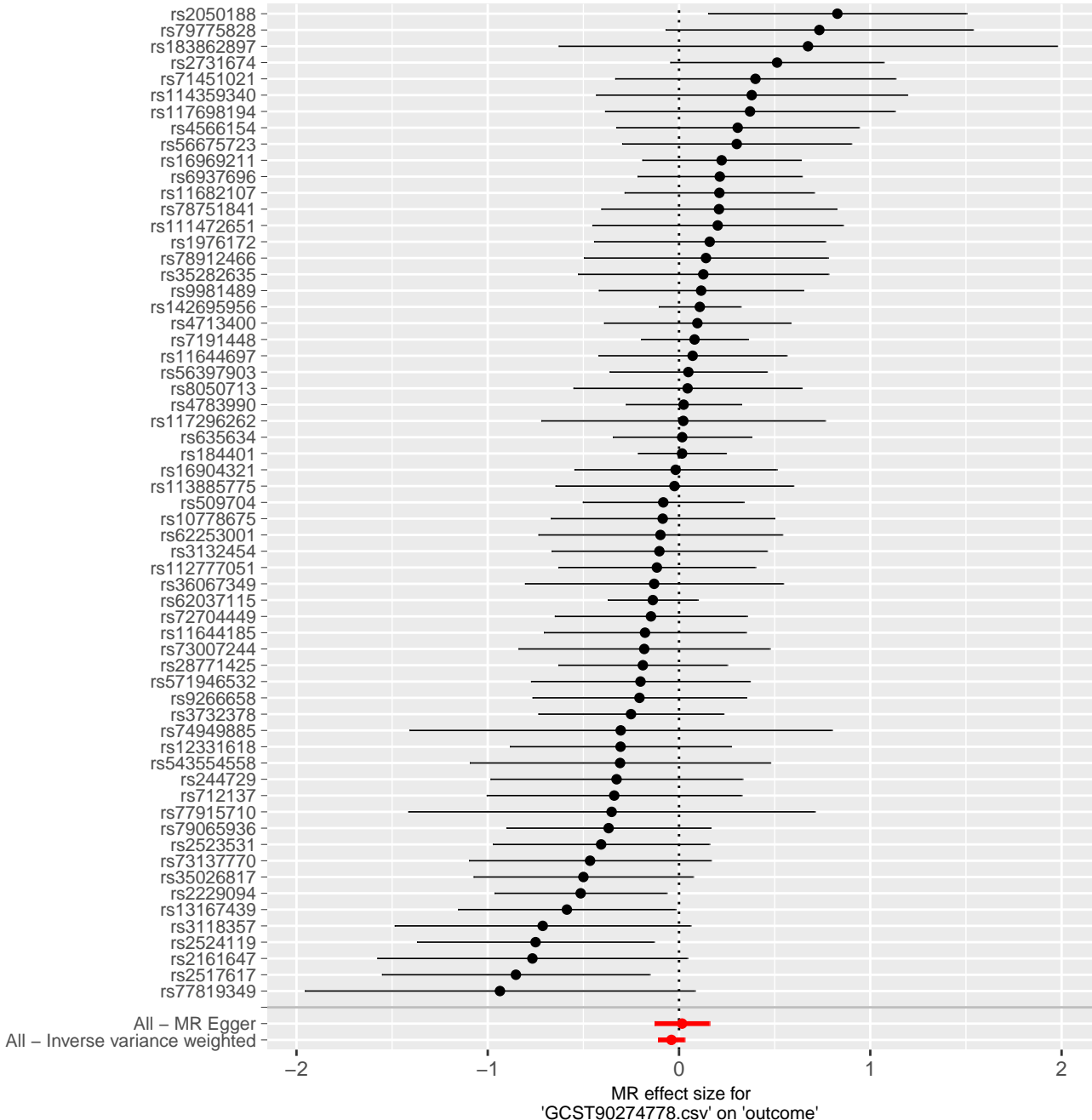

Supplement: Supplementary file 1 [file DataSheet_1.zip › supplementary material/CIM-OSTEOPOROSIS /GCST90274778.csv_forest.pdf]

# MR Method

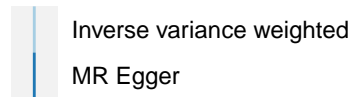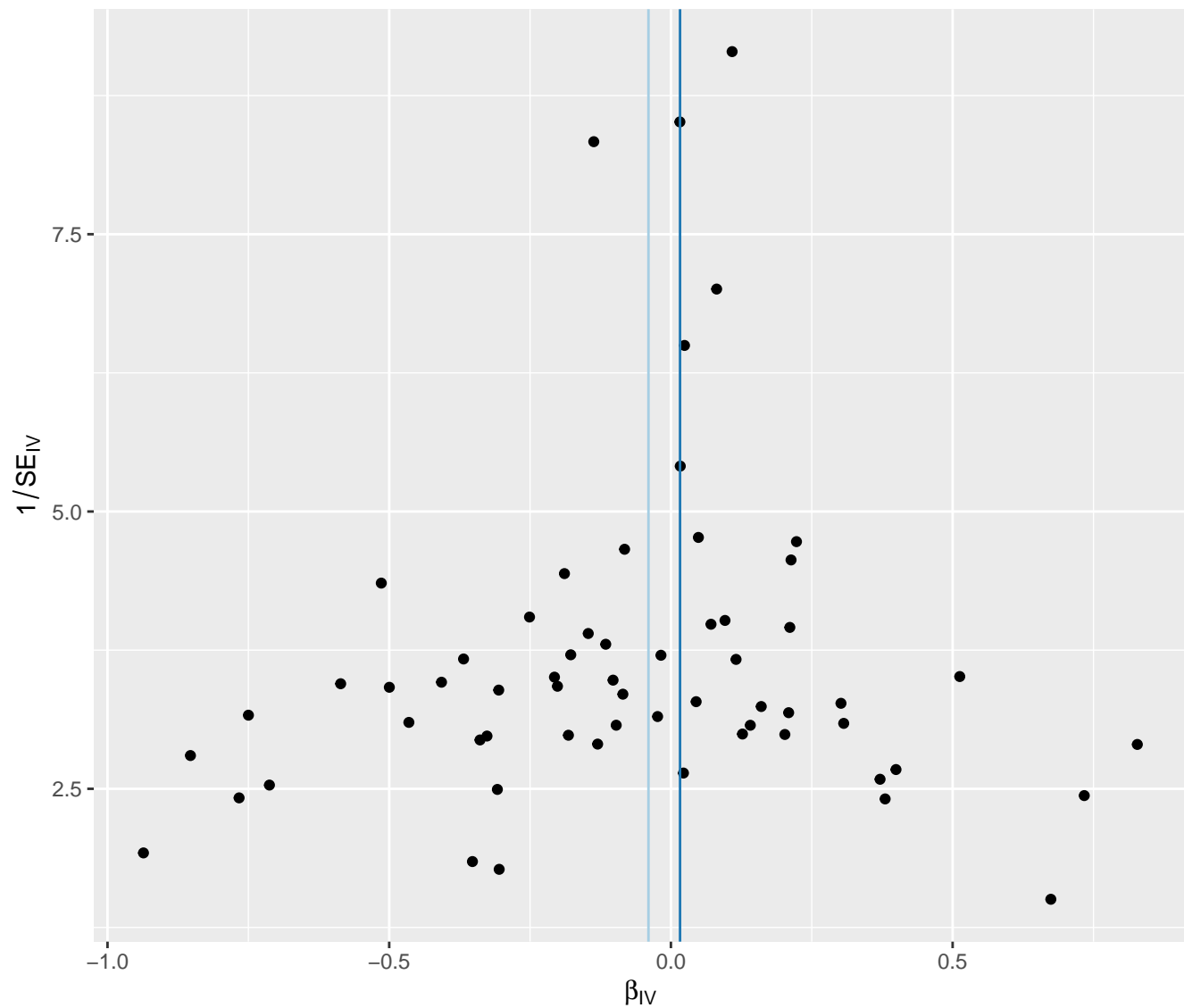

Supplement: Supplementary file 1 [file DataSheet_1.zip › supplementary material/CIM-OSTEOPOROSIS /GCST90274778.csv_funnel.pdf]

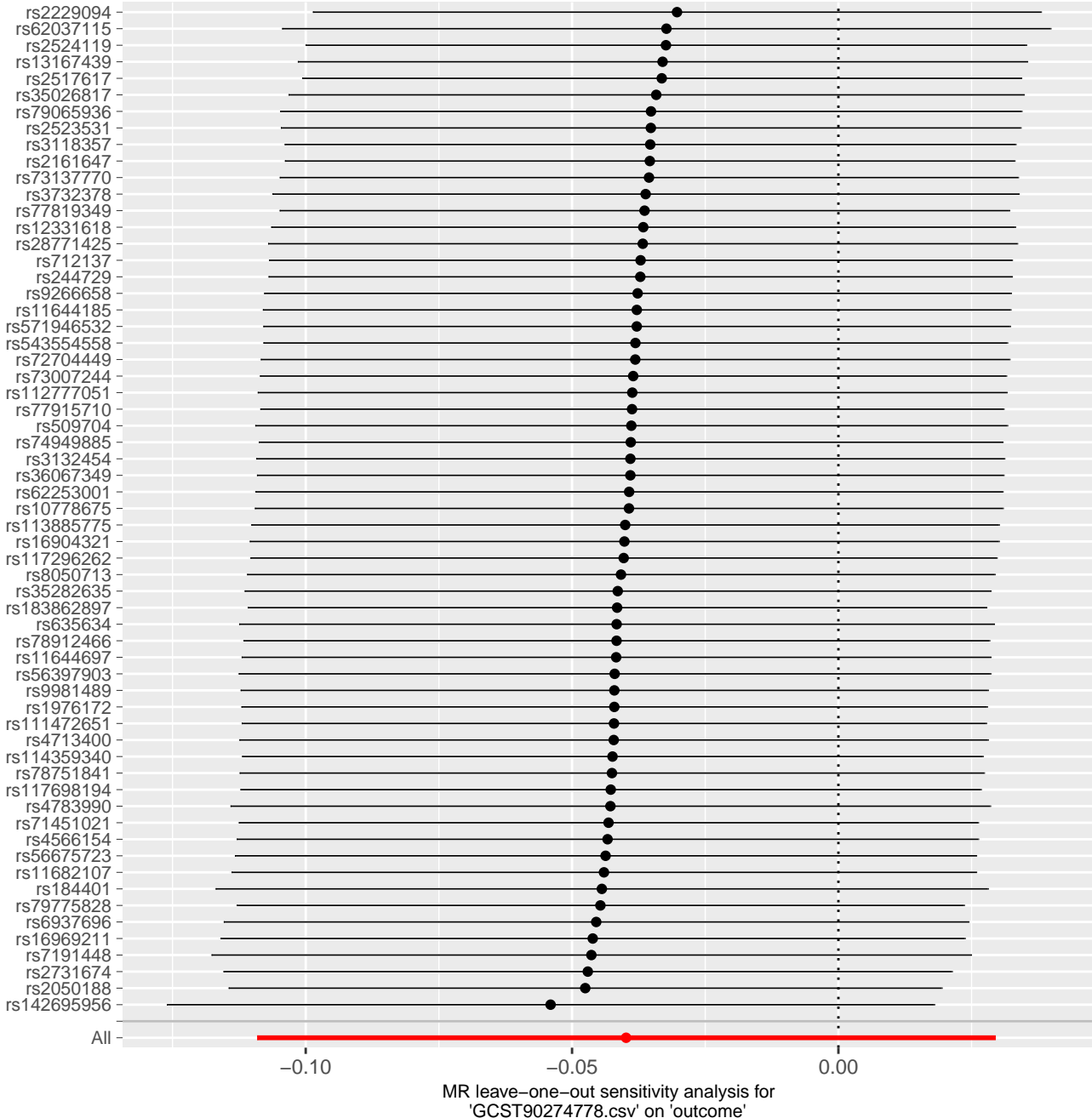

Supplement: Supplementary file 1 [file DataSheet_1.zip › supplementary material/CIM-OSTEOPOROSIS /GCST90274778.csv_leave_one_out.pdf]

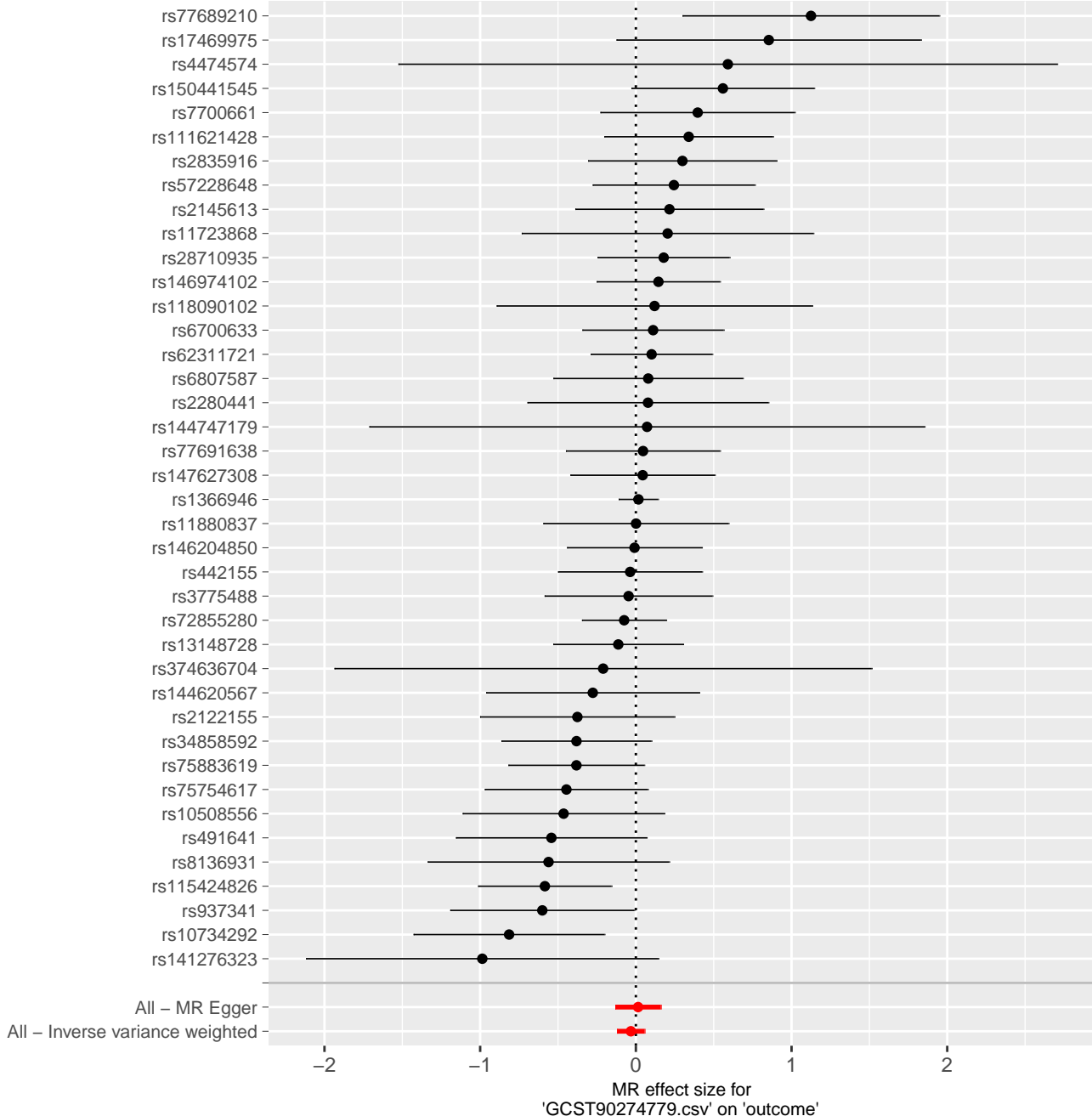

Supplement: Supplementary file 1 [file DataSheet_1.zip › supplementary material/CIM-OSTEOPOROSIS /GCST90274779.csv_forest.pdf]

# MR Method

- Inverse variance weighted
- MR Egger

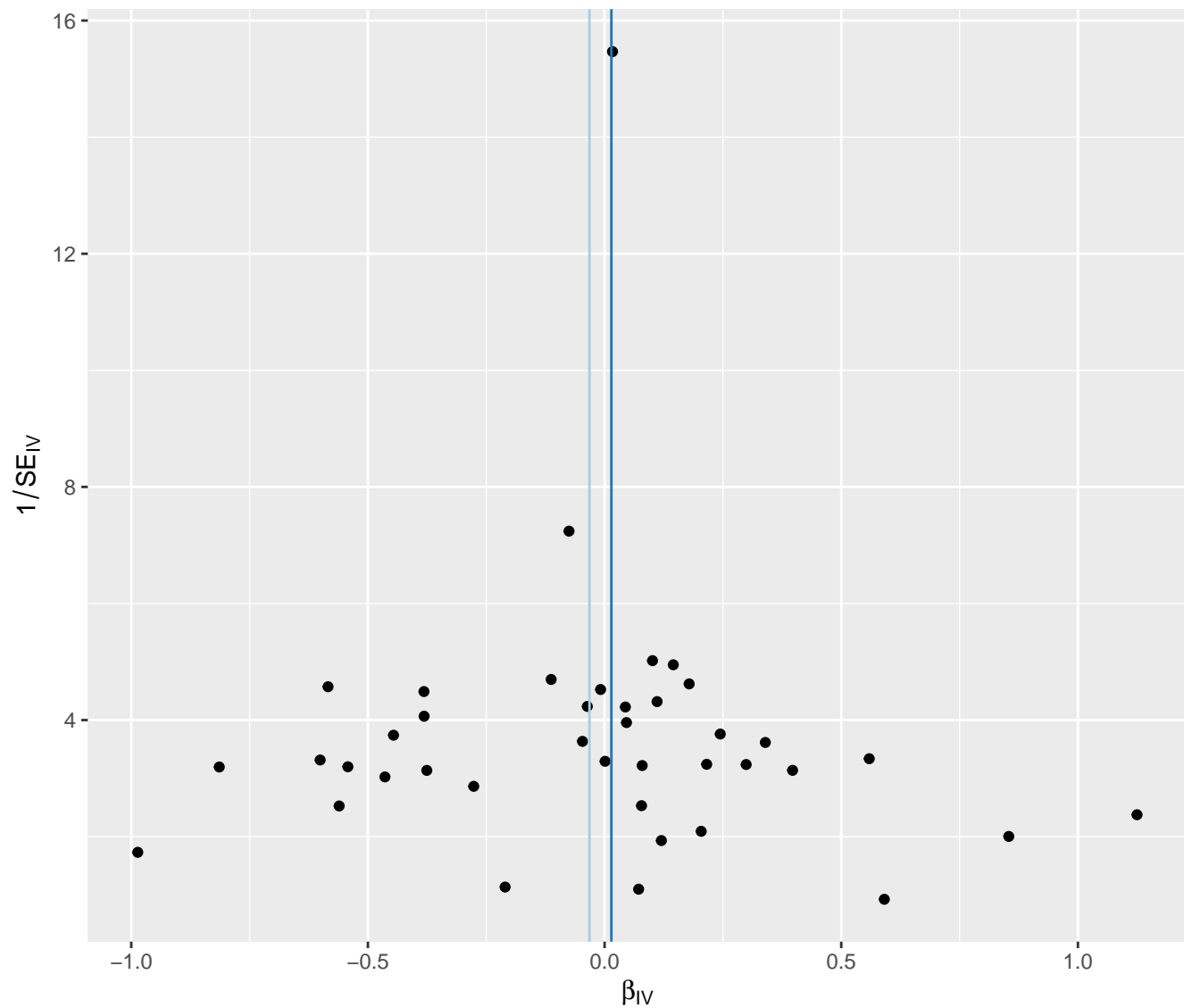

Supplement: Supplementary file 1 [file DataSheet_1.zip › supplementary material/CIM-OSTEOPOROSIS /GCST90274779.csv_funnel.pdf]

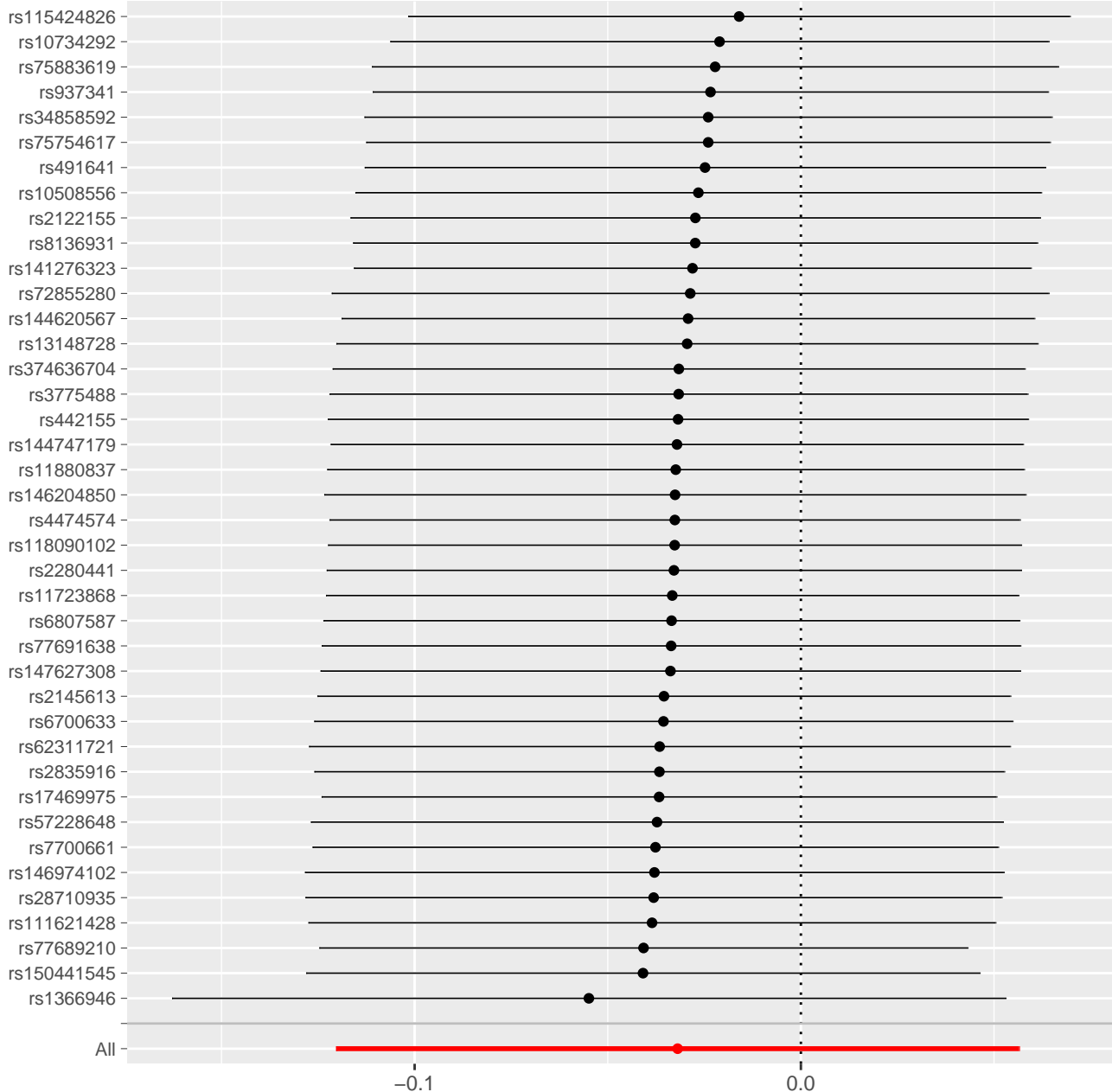

MR leave-one-out sensitivity analysis for  
'GCST90274779.csv' on 'outcome'

Supplement: Supplementary file 1 [file DataSheet_1.zip › supplementary material/CIM-OSTEOPOROSIS /GCST90274779.csv_leave_one_out.pdf]

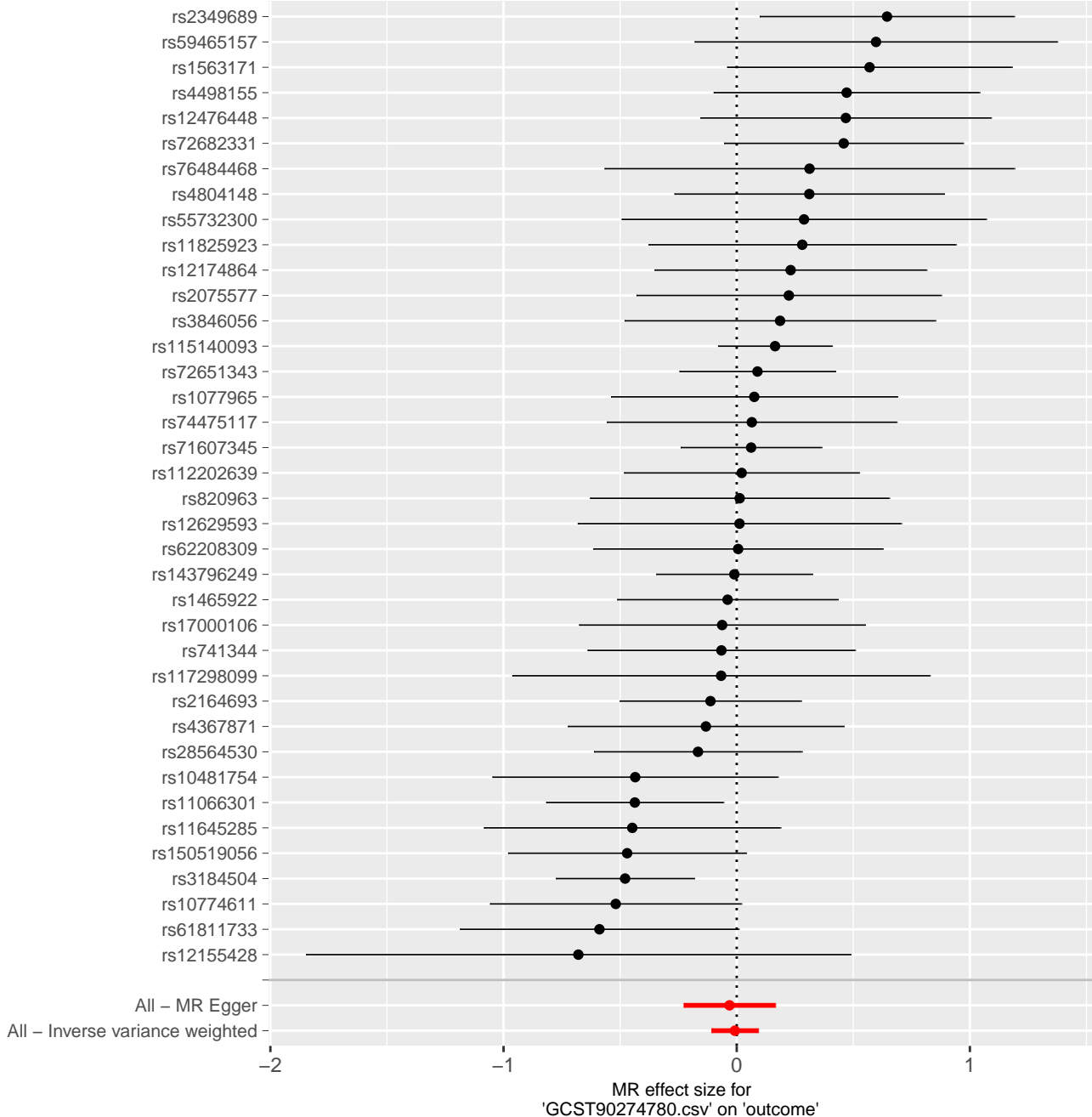

Supplement: Supplementary file 1 [file DataSheet_1.zip › supplementary material/CIM-OSTEOPOROSIS /GCST90274780.csv_forest.pdf]

# MR Method

- Inverse variance weighted
- MR Egger

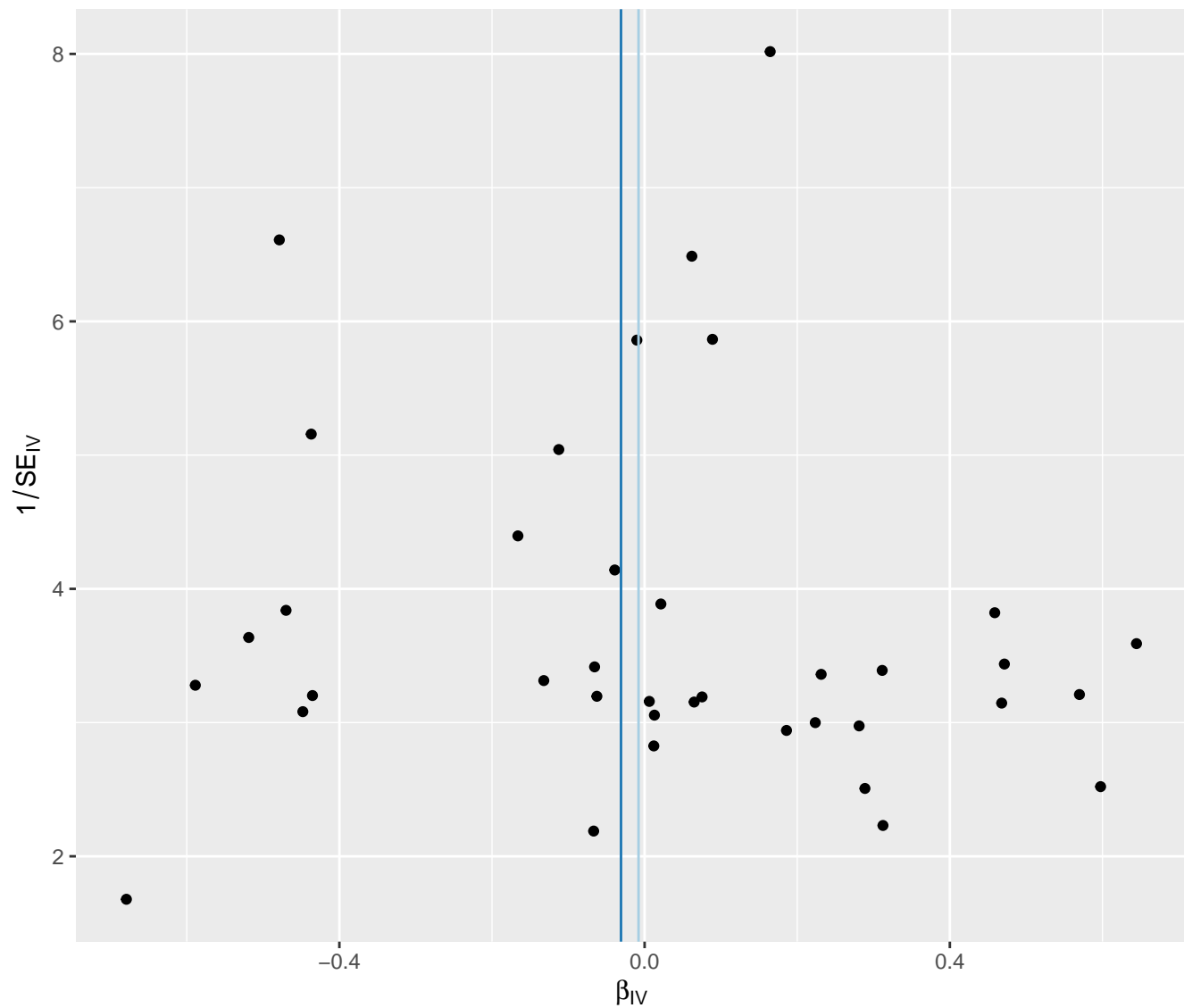

Supplement: Supplementary file 1 [file DataSheet_1.zip › supplementary material/CIM-OSTEOPOROSIS /GCST90274780.csv_funnel.pdf]

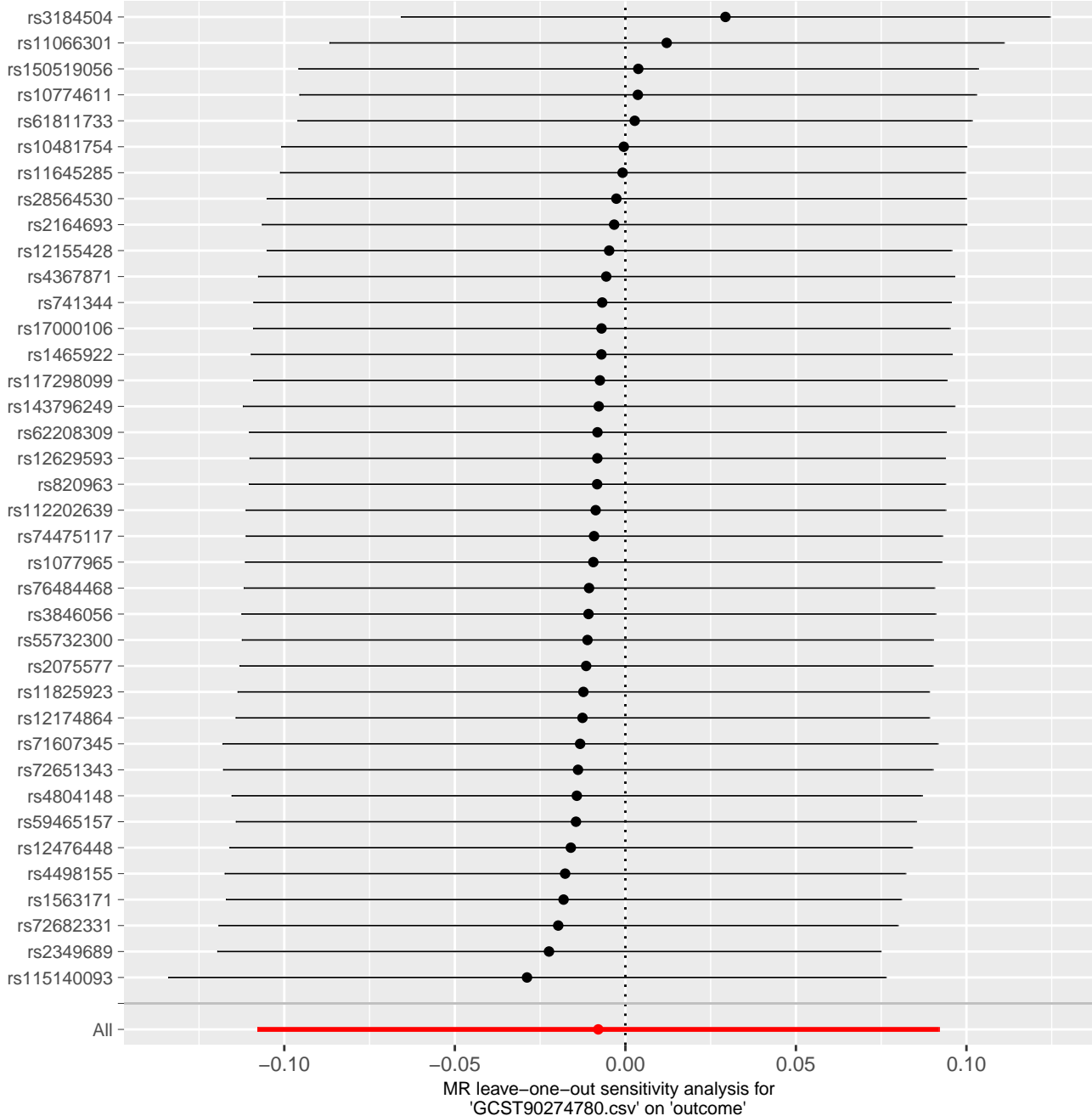

Supplement: Supplementary file 1 [file DataSheet_1.zip › supplementary material/CIM-OSTEOPOROSIS /GCST90274780.csv_leave_one_out.pdf]

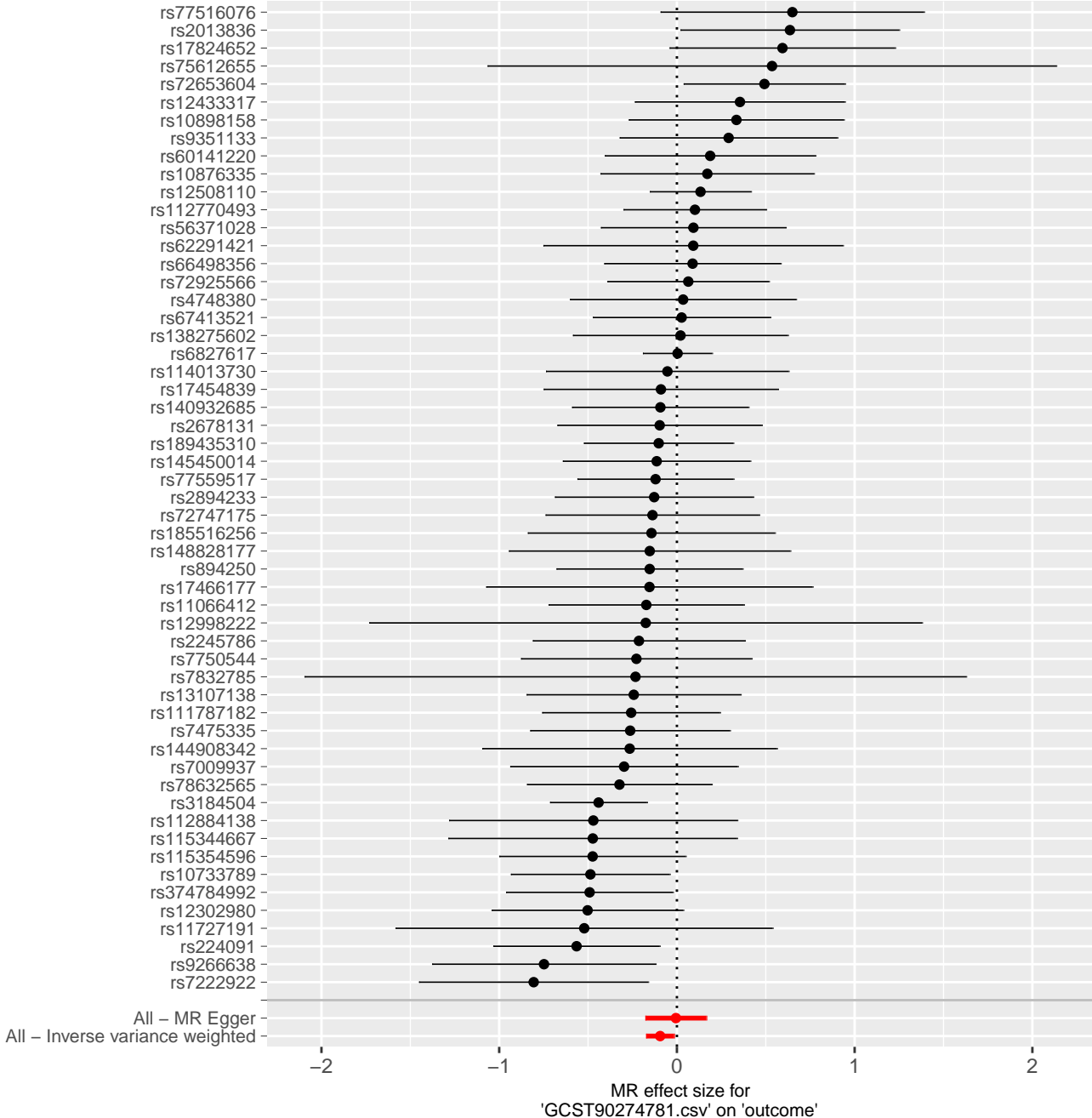

Supplement: Supplementary file 1 [file DataSheet_1.zip › supplementary material/CIM-OSTEOPOROSIS /GCST90274781.csv_forest.pdf]

# MR Method

- Inverse variance weighted
- MR Egger

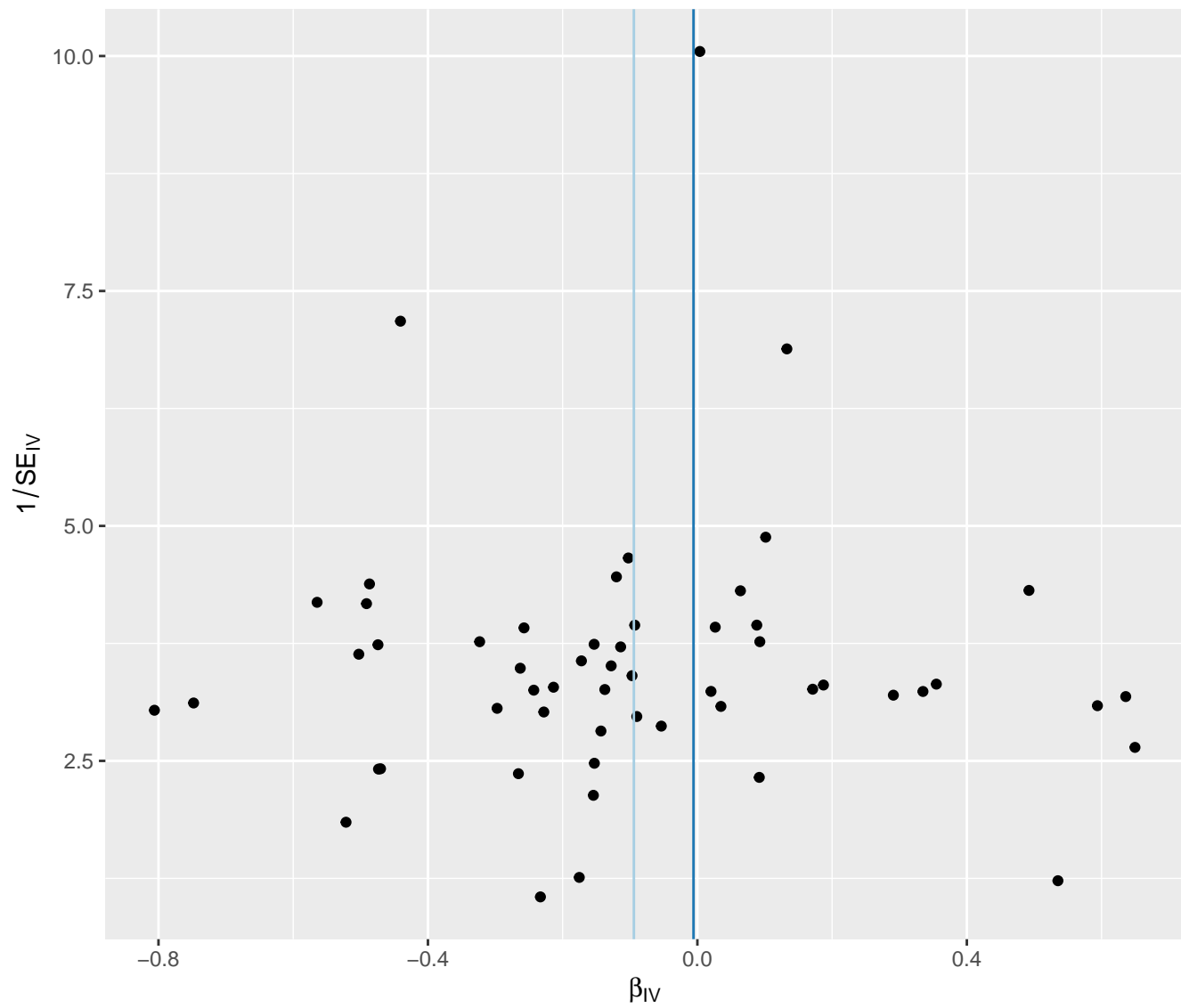

Supplement: Supplementary file 1 [file DataSheet_1.zip › supplementary material/CIM-OSTEOPOROSIS /GCST90274781.csv_funnel.pdf]

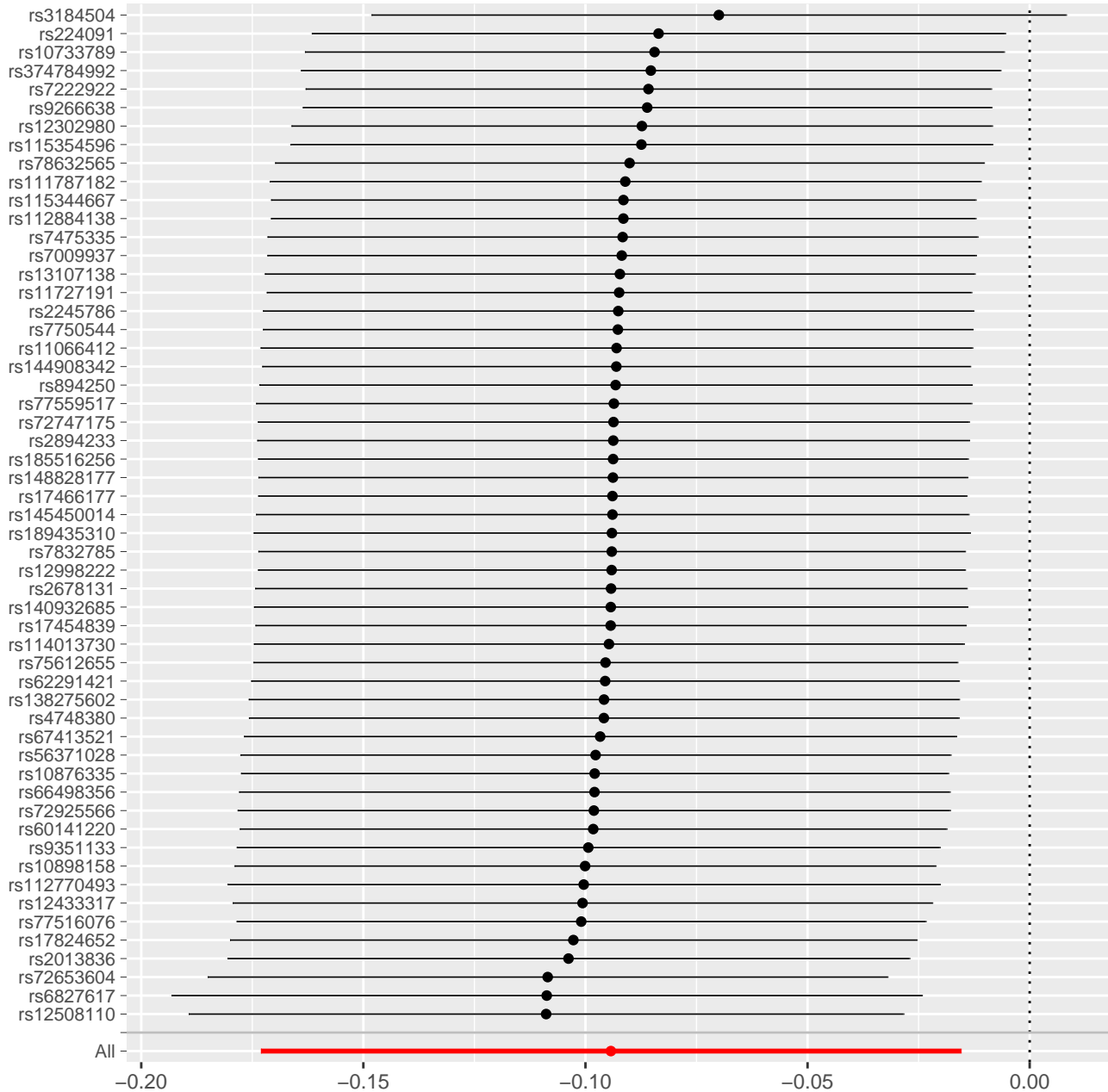

Supplement: Supplementary file 1 [file DataSheet_1.zip › supplementary material/CIM-OSTEOPOROSIS /GCST90274781.csv_leave_one_out.pdf]
